# Light-Induced Rotation of a Molecular Motor in the Confined Space of a Metal–Organic Nanocage

**DOI:** 10.1021/jacs.5c16349

**Published:** 2026-01-26

**Authors:** Carles Fuertes-Espinosa, Marco Ovalle, Yohan Gisbert, Clara Sabrià, Valentina Iannace, Josep M. Luis, Ferran Feixas, Alexander Ryabchun, Xavi Ribas, Ben L. Feringa

**Affiliations:** † Institut de Química Computacional i Catàlisi (IQCC) and Departament de Química, 16738 Universitat de Girona, Campus Montilivi, 17003 Girona, Catalonia, Spain; ‡ Stratingh Institute for Chemistry, 3647 University of Groningen, 9747AG Groningen, The Netherlands

## Abstract

Molecular motors have been operated in a myriad of environments since their inception more than two decades ago. Of particular interest are systems in which a structural frame of reference for the motor’s rotary motion is established. Examples include motors adsorbed on surfaces, embedded in framework materials, used as dopants in liquid crystals, or incorporated into polymer matrices. Embedding the molecular motor as a guest in a supramolecular cage assembly, however, remains an unexplored strategy. Such systems are particularly attractive, as they would allow the motor’s rotation to drive mechanical motion from within the assembly, provided the motion does not lead to the guest motor’s release. Here, the first motor–nanocage host–guest system in which a thermal and light-driven full 360° unidirectional rotational cycle occurs within the confined space of the nanocage is reported. We identified key structural elements that enable the formation of a host–molecular motor complex with unprecedented stability, governed by a noncovalent interaction between the motor’s alkyl–COOH moiety and a carbonyl residue on the cage. This strategy allows the formation of stable host–guest complexes without relying on a size-induced fit, as is commonly observed in other inclusion complexes. This enables rotation to occur within the cage cavity despite dramatic geometric changes. We envision this strategy as a valuable tool for developing a new generation of molecular motors operating in confined spaces.

## Graphical abstract

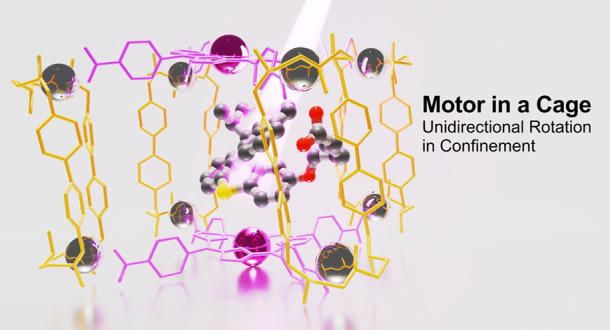

## Introduction

Biological molecular machinery,^1^ which is a source of inspiration for the design of their artificial counterparts,^2–8^ rarely function as a stand-alone entity in nature, but instead operates within complex, multicomponent assemblies.^9^ While systems like the F_1_F_0_ ATP synthase^10^ are functionally coupled to the membrane environment, a particularly striking example of motion within structural confinement is found in the DNA packaging motors of bacteriophages.^11,12^ These molecular assemblies drive the translocation of DNA into the confined space of a viral capsid, performing mechanical work against substantial entropic and electrostatic resistances (Figure 1a). Such systems highlight how biological function often emerges from the interplay between directional motion and spatial confinement—a principle largely unexplored in the field of artificial molecular motors.

**Figure 1:**
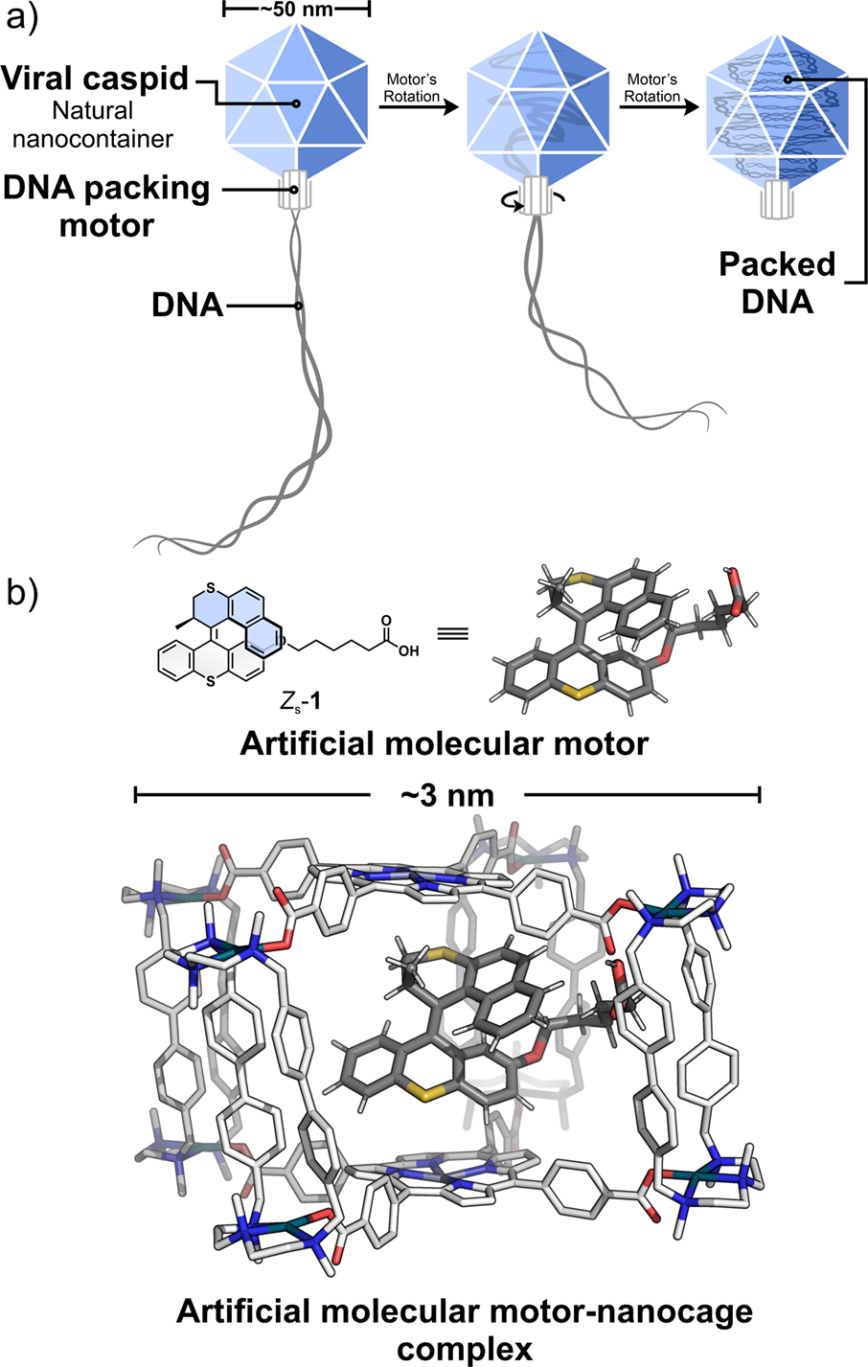
(a) DNA packing process driven by a biological motor-nanocontainer (viral capsid) dyad.^11,12^ (b) Artificial molecular motor-nanocage complex (this work).

Since the development of the first light-powered artificial molecular motor in 1999,^13^ the field has experienced continuous growth across multiple frontiers.^14–17^ In particular, the incorporation of molecular motors into diverse environments has been the key to harnessing their directional rotation. Examples include assembly on surfaces,^18–21^ integration into framework materials,^22–26^ membranes,^27–29^ liquid crystals^30–33^ and polymer networks.^34–36^ These environments provide a structural frame of reference for motor rotation, which is crucial to taking advantage of their mechanical functions.

In parallel, the field of nanocages also experienced a spectacular growth.^37–42^ These materials have been successfully used to perform a wide variety of chemistries both within their peripheral structure and in their confined space. Selected examples include molecular recognition,^43–45^ stabilization of reactive species,^46–49^ catalysis,^50–53^ and delivery systems.^54,55^

Molecular switches^56^ and molecular cages have proven to be a synergistic pair in supramolecular design.^57–61^ This combination has enabled the development of systems that exhibit stimuli-responsive molecular recognition,^62–65^ dynamic transformations,^66–69^ mass transport,^70,71^ and control over dynamic function.^72,73^ Despite this progress, however, molecular motors have rarely been embedded into cage systems.^74,75^ In contrast to switches, molecular motors offer the unique advantage of performing nonreciprocal, continuous rotary motion.^76,77^ Much like the aforementioned DNA packaging motors, the motor–cage pairing has the potential to synergistically enhance both components, enabling functions that would not be achievable by either element alone.

Here we report the first operational molecular host–guest motor cage system (Figures 1b and 2a). The supramolecular dyad is composed of a light-driven second-generation^78^ rotary motor **1** and heteroleptic metal–organic cage **(MOC) 4**·(BArF)_8_. Motor **1** is functionalized with an aliphatic tail featuring a carboxylic acid end group, while cage **4**·(BArF)_8_ is based on Zn-TCPP (5,10,15,20-tetrakis (4-carboxyphenyl)-porphyrin Zn­(II)) and bimetallic Pd­(II) macrocyclic complexes, featuring a tetragonal prismatic geometry. This cage has previously shown high selective affinity toward fullerenes mixtures,^79–83^ as well as strong binding of pyridine-based catalysts through simultaneous anchoring at the apical position of the two Zn-TCPP moieties.^84,85^ Here we demonstrate that the cage has also unprecedented capability to form a highly stable host-molecular motor complex. This occurs through a H-bond that serves as an anchor between the carboxylic acid group of the motor’s tail and the carbonyl residues present inside the cage cavity. By relying on this specific noncovalent interaction, the host–guest complex displays a unique feature that differs from most inclusion complexes in which a size induce fit is indispensable. In fact, the host–guest system between **1** and **4**·(BArF)_8_ has a volume occupancy of only 24%, far from the 55% stablished by the Rebek’s rule.^86^

**Figure 2:**
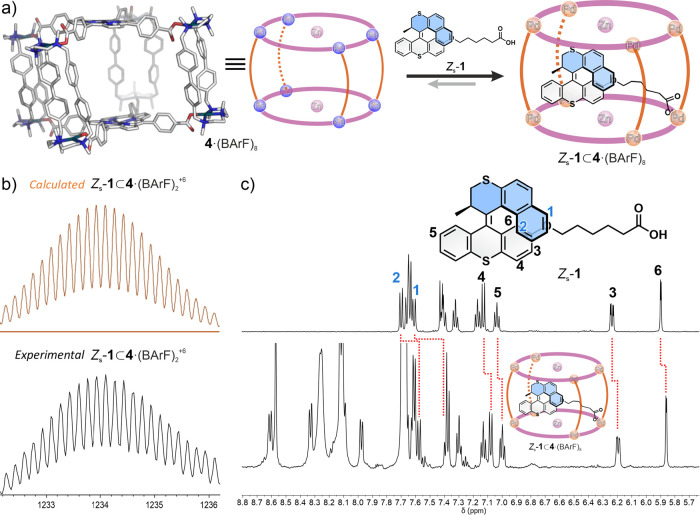
(a) Schematic representation of the binding of molecular motor *Z*_s_-**1** within cage **4**·(BArF)_8_ and the anchoring interaction based on a hydrogen bond between the motor tail and the porphyrin residue. (b) HRMS spectra (calcd and exp.) of *Z*_s_-**1** ⊂ **4**·(BArF)_8_ (+6 peak shown, see SI for details). (c) Stacked partial **^1^** H NMR spectra of motor *Z*_s_ **-1** and *Z*_s_-**1** ⊂ **4**·(BArF)_8_, showing spectral changes upon the addition of molecular motor *Z*_s_-**1** into a solution of **4**·(BArF)_8_ (shielding effect is consistent for all the protons of the Z_s_-**1** (Δδ 0,13-Δδ 0,19 ppm).

We attribute the volume independence of the motor-cage complex stability to be a significant factor in allowing uncompromised motor rotation in the cage confined space. Indeed, we observe the full rotation cycle of motor **1** inside **4**·(BArF)_8_ with almost no alteration in the motor’s behavior inside or outside of the cage. These results open the door to designing new generations of motor–nanocage supramolecular systems whose operation is no longer restricted by simple size-matching effects. This includes creating customized cages that permit free rotation in the solid state, cages that support energy-transfer processes, architectures with multiple motor attachment sites, and combinations of motors and cages of various dimensions. A deeper understanding of these behaviors could ultimately lead to integrated motor–cage assemblies with emergent mechanical functions, resembling—at least conceptually—the action of biological DNA-packing motors.

## Results and Discussion

### Synthesis and Characterization of Motor **1** and *Z*_s_-**1**⊂**4**·(BArF)_8_ Host–Guest Complex

Molecular motor *Z*_s_-**1** (Figure 2a,c) was synthesized from the reported motor **2** ^78^ (Figure 3a) via successive methoxy deprotection and etherification with the side chain (see SI for details about the synthesis).

**Figure 3:**
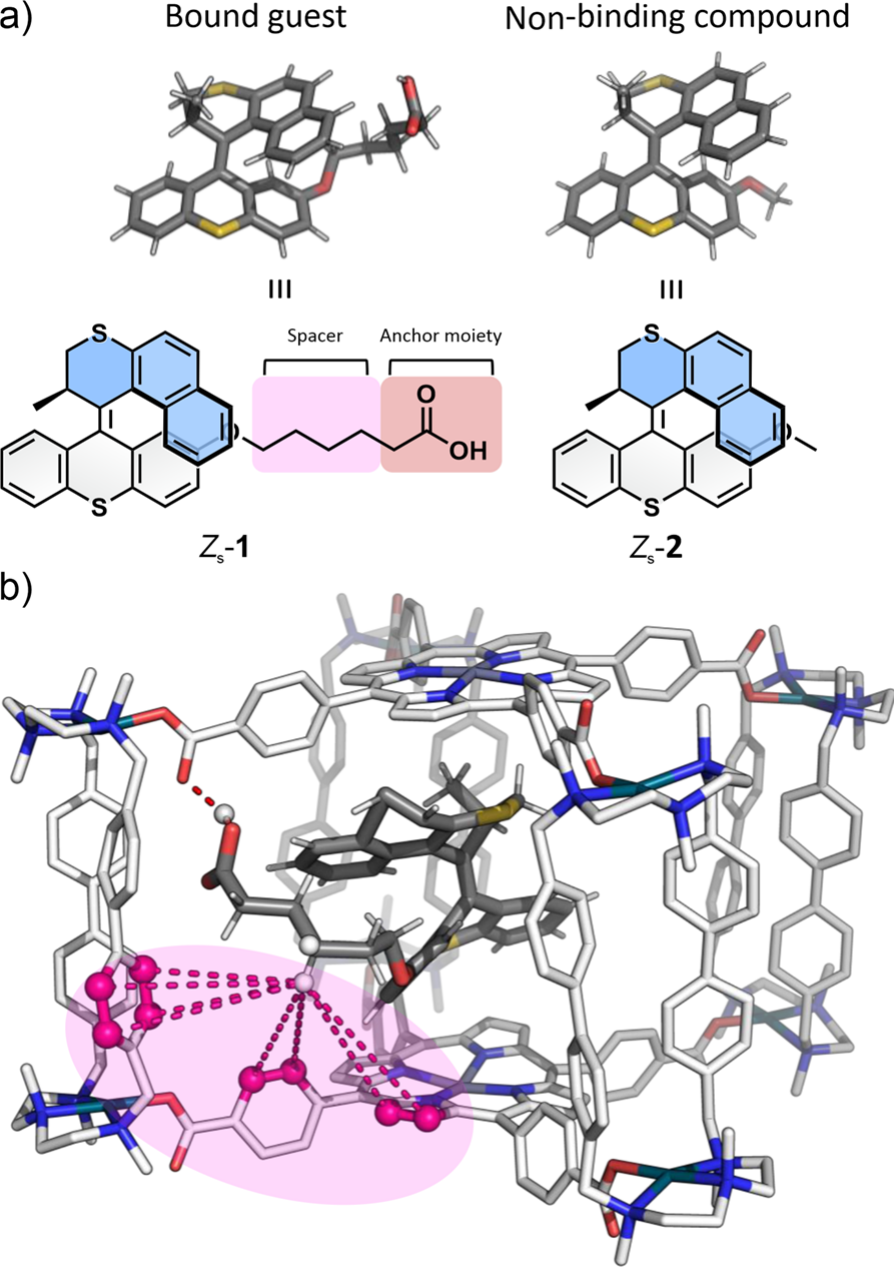
(a) Structure of bound guest motor **1** in its *Z* stable configuration and the nonbinding motor **2** in the same configuration. (b) Representative structure obtained from molecular dynamic simulations on the encapsulation of motor (*R*)-(*M*)-*Z*_s_ *-* **1** in the cavity of nanocage **4**·(Cl)_8_, revealing hydrogen bonding interaction between the carboxylic acid tail of the motor (hydrogen bond donor) and one carbonyl residue of **4**·(Cl)_8_ (hydrogen bond acceptor) (marked in orange). Additionally, NOESY-NMR interactions between some CH_2_ moieties of the motor tail and the aromatic and pyrrole C–H’s from the cage (marked in pink). Counter ions (Cl^–^) and hydrogens of the cage are not shown for clarity.

The encapsulation of *Z*_s_-**1** within the tetragonal prismatic cage **4**·(BArF)_8_ was achieved by addition of stoichiometric amounts of the guest into a CD_3_CN solution of the host (Figure 2).^79^

The corresponding host–guest complex was analyzed by NMR (Nuclear Magnetic Resonance) and HRMS (High-Resolution Mass Spectrometry). ^1^H NMR spectroscopy showed that **4**·(BArF)_8_ was able to accommodate one equivalent of *Z*_s_-**1** (Figures 2c and S8). The signals of the guest underwent an upfield shift attributed to an inclusion-induced shielding effect, consistent with guest binding within the cavity of **4**·(BArF)_8_ in the fast exchange regime on the NMR time scale (Figures 2c and S2). The signals corresponding to the protons of the metalloporphyrin residues of **4**·(BArF)_8_ pointing inward became broader and slightly downfield shifted, suggesting guest binding in the vicinity of these porphyrin panels (Figures 2c, inset, S2 and S8).

^1^H DOSY NMR data for *Z*_s_-**1 ⊂ 4**·(BArF)_8_ revealed a diffusion-coefficient of *D* = 3.1 × 10^–10^ m^2^ s^–1^, corresponding to solvodynamic diameter of 19.0 Å, which is in line with the dimensions of empty **4**·(BArF)_8_, and consistent with an internal binding of *Z*_s_-**1** (see Figure S40). The inclusion complex *Z*_s_-**1 ⊂ 4**·(BArF)_8_, was further confirmed by HRMS, observing molecular ions attributed to a 1:1 host–guest adduct (see Figure S6).

In addition, ^1^H NMR titrations allowed the elucidation of the host–guest stoichiometry, observing results consistent with the formation of a 1:1 host–guest complex (see Figure S8). The binding strength was quantified through ultraviolet–visible (UV–Vis) spectroscopy titration, resulting in a strong association constant (*K*_a_) of 2.3 (±0.5)×10^5^ M^–1^ (see Figure S7). Both enantiomers of *Z*_s_-**1**: (*R*)-(*M*)-*Z*_s_-**1** and (*S)-(P*)-*Z*_s_-**1** were separated using chiral HPLC (see SI section I.1 and IX for details). The chiral nature of the confined *Z*_s_-**1 ⊂ 4**·(BArF)_8_ was studied by circular dichroism (CD) spectroscopy, confirming that the dynamic chirality of the motor is preserved within the confined space (vide infra, Figures 4d and S36).

**Figure 4:**
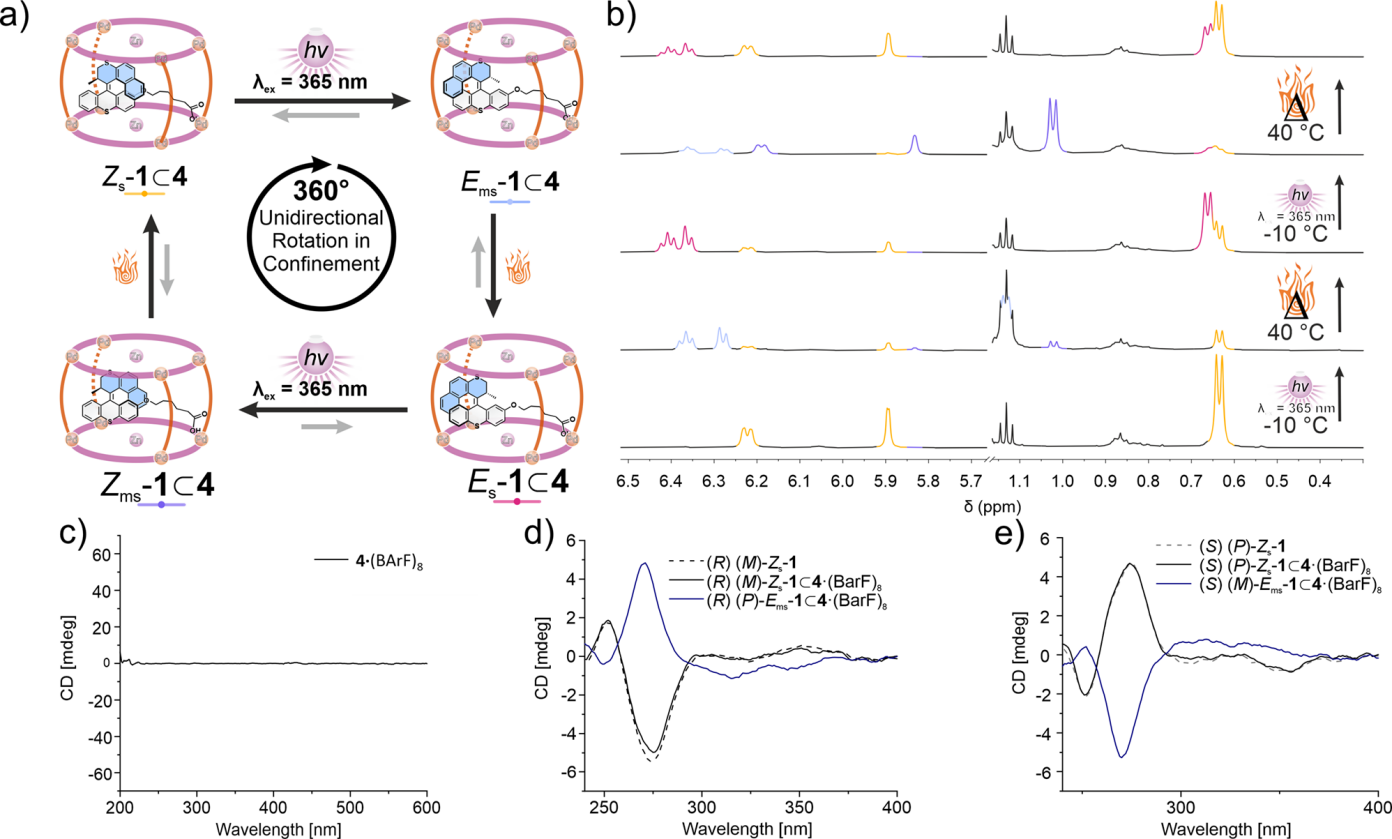
(a) Schematic representation of the 4-step rotation cycle starting from *Z*_s_-**1** ⊂ **4**·(BArF)_8_. (b) Stacked partial **^1^** H NMR spectra (from bottom to top) of *Z*_s_-**1** ⊂ **4**·(BArF)_8_, *E*_ms_-**1** ⊂ **4**·(BArF)_8_, *E*_s_-**1** ⊂ **4**·(BArF)_8_ and *Z*_ms_-**1** ⊂ **4**·(BArF)_8_. (c) CD spectrum (acetonitrile, ∼3 × 10^–6^ M, 20 °C) of **4**·(BArF)_8_ (no CD signals observed). (d) CD spectra (acetonitrile, ∼1 × 10^–6^ M motor and ∼3 × 10^–6^ M cage, 20 °C) of (*R*)-(*M*)-*Z*_s_-**1** before irradiation in bulk solution (dashed), (*R*)-(*M*)-*Z*_s_-**1** ⊂ **4**·(BArF)_8_ at the same concentration (black) and (*R*)-(*P*)-*E*_ms_-**1** ⊂ **4**·(BArF)_8_, obtained after irradiation at 365 nm (blue). (e) CD spectra (acetonitrile, ∼1 × 10^–6^ M motor and ∼3 × 10^–6^ M cage, 20 °C) of stable (*S*)-(*P*)-*Z*_s_-**1** before irradiation in bulk solution (dashed), (*S*)-(*P*)-*Z*_s_-**1** ⊂ **4**·(BArF)_8_ at the same concentration (black) and (*S*)-(*M*)-*E*_ms_-**1** ⊂ **4**·(BArF)_8_ obtained after irradiation at 365 nm (blue).

### Nature of the Interaction Motor ⊂ Cage Host–Guest Complex

We hypothesized that a contribution of the coordination of the sulfur atoms present in the guest, toward the Zn-TCPP residues paneling two opposite faces of **4**·(BArF)_8_, could be a factor in the driving force promoting the molecular recognition of *Z*_s_-**1**. In order to get more insight into the nature of the binding event, the encapsulation of motor **2** ^87^ was studied in a control experiment. The structure of **2** differs from that of **1** exclusively by the presence of a methoxy substituent instead of the side aliphatic chain bearing a terminal carboxylic group (Figure 3a). Following the same experimental protocols as those used, we attempted the binding of *Z*_s_-**2**. Surprisingly, no evidence for the formation of *Z*_s_-**2 ⊂ 4**·(BArF)_8_ host–guest complex was detected, neither by ^1^H NMR, UV–Vis (see Figures S9 and S10) or HRMS. Therefore, it can be concluded that the CO_2_H– terminated alkyl chain present at *Z*_s_-**1** is crucial for the binding of molecular motors within **4**·(BArF)_8_, whereas contributions of the sulfur atoms seem negligible.

Molecular dynamics (MD) simulations were performed to rationalize the molecular interactions responsible for the selective binding of *Z*_s_-**1** in the cavity of **4**·(Cl)_8_. Multiple replicas of 500 ns unconstrained and restrained MD simulations were initiated with either *Z*_s_-**1** or *Z*_s_-**2** positioned inside the cavity (see SI section IV), revealing significantly different behavior for both systems. A persistent hydrogen bond was identified between the carboxylic acid moiety of *Z*_s_-**1** and one of the eight carbonyl groups of the carboxylate moieties in **4**·(Cl)_8_, effectively retaining the guest within the cavity (Figure S12). In contrast, *Z*_s_-**2**, which lacks the alkyl-COOH tail, exhibited fast unbinding from the cavity (Figure S11). This suggests that the carboxylic acid group plays a crucial role in anchoring the molecular motor, while the long alkyl chain allows the motor to be positioned unrestricted within the cavity (see Figure S12 for further details). These computational results are in perfect agreement with the experimental evidence obtained from the binding analysis for nanocage **4**·(BArF)_8_ with *Z*_s_-**2** and *Z*_s_-**1**.

In both *Z*_s_-**1** and *Z*_s_-**2**, MD simulations showed transient weak interactions between the sulfur atoms in the motor and Zn porphyrins (see Video S1). However, these interactions were insufficient to confine the nonalkylated motor within the cavity. For *Z*_s_-**1**, multiple orientations coexisted within the cavity of **4**·(Cl)_8_. Although not explicitly observed within the simulation time scale, the COOH anchoring likely alternates between the eight equivalent carbonyl groups.

Nuclear Overhauser Effect Spectroscopy (NOESY) NMR showed correlations between hydrogen atoms in the aliphatic chain of *Z*_s_-**1** and ^1^H NMR signals assigned to inward hydrogen atoms near the carbonyl groups at the **4**·(BArF)_8_ inner cavity (Figures 3 and S5). These results agreed with the MD simulations, thus confirming that the alkyl-COOH functional group is a crucial anchoring handle for *Z*_s_-**1** binding. Additionally, docking *Z*_s_-**1** within the cavity of the crystal structure of **4**·(BArF)_8_, reveals an occupancy volume of only 24% (Figure S13),^36^ being far from the optimal 55% Rebek’s volume occupancy rule.^86^ Hence, all these considerations point toward the anchoring alkyl-COOH tail being essential for the encapsulation of *Z*_s_-**1** within **4**·(BArF)_8_. Furthermore, this specific binding mode allows for sufficient free cavity volume and high stability of the host–guest complex (*K*_a_ = 2.3 (±0.5) × 10^5^ M^–1^), potentially generating an inclusion complex capable of maintaining the host–guest interaction upon conformational changes and small volume variations of the confined guest.

### Rotary Behavior of Motor **1** in Solution

Thermal and light-driven molecular motors typically undergo unidirectional rotation by a four-step cycle.^16^ Initially, photochemical *E*/*Z* isomerization yields a metastable isomer, which relaxes via thermal helix inversion (THI) to a stable state. This process is repeated to yield a 360° revolution around the double bond.^78,88^ Study of the rotary cycle of motor **1** in solution started by the in situ ^1^H NMR irradiation (λ_irr_ = 365 nm, 5 min, −10 °C) of pristine stable *Z*_s_-**1** at −10 °C (step I, Figures S15–S17) in order to prevent the THI to facilitate the analysis. After 5 min of irradiation, 75% of the *E*_ms_-**1** isomer is obtained (See Table S1 for the full isomeric distribution). This photoisomerization step was also studied by CD, revealing sign inversions of the two main Cotton effects at 217 and 273 nm (Figures 4d,e, and S23–S26). This observation is consistent with the usual behavior of overcrowded-alkene molecular motors, undergoing helicity inversion upon *E*/*Z* photoisomerization. Subsequent heating in the absence of light allowed for the first THI of the cycle to take place (Step II, Figure S18) as is observed by ^1^H NMR. After 16 h of monitoring the system at 40 °C, stable *E*_s_-**1** was obtained. Further heating until no metastable isomer was observed led to an isomeric abundance of 76%. Subsequent photoisomerization was achieved by irradiation (λ_irr_ = 365 nm, 5 min, −10 °C) allowing the formation of *Z*_ms_-**1** in an isomeric abundance of 60% (step III, Figures S19 and S20). Finally, heating the mixture (monitored for 16 h at 40 °C and further heated until no metastable isomer was observed, step IV, Figures S21 and S22) completes the cycle, yielding 63% of *Z*_s_-**1**. The observation of the different intermediates of the four-step cycle confirms unambiguously the unidirectional rotation of the motor.

### Rotary Behavior of Motor **1** in the Confined Space of **4**·(BArF)_8_

The rotary behavior of *Z*_s_-**1** in the confined environment of **4**·(BArF)_8_ was studied by ^1^H NMR, submitting the host–guest adduct to the same experimental conditions used to elucidate the rotation behavior of the *Z*_s_-**1** motor in bulk solution.

Due to the presence of oxygen, however, the **1 ⊂ 4**·(BArF)_8_ complex experienced degradation (Figure S27). Since **4·**(BArF)_8_ behave as a photosensitizer, singlet oxygen was produced, oxidizing and degrading the guest.^89^

In order to avoid this process, NMR monitoring of the rotation in the confined space was performed in a J Young-NMR tube degassed by the freeze–pump–thaw method. Hence, the motor’s rotation in the confined space of the cage was monitored by ex-situ irradiation (λ_irr_ = 365 nm, 5 °C, 5 min) of *Z*_s_-**1 ⊂ 4**·(BArF)_8_ to obtain *E*_ms_-**1 ⊂ 4**·(BArF)_8_ (step I, Figures S28 and S29). The signals of motor **1** present an upfield shift, consistent with the guest binding within the cavity of **4**·(BArF)_8_ (Figures 4 and S28).

Increasing the temperature to 40 °C allowed the first THI to occur, promoting the complete relaxation of *E*_ms_-**1 ⊂ 4·**(BArF)_8_ to the stable *E*_s_-**1 ⊂ 4·**(BArF)_8_ (Figure S30). A second ex-situ irradiation (λ_irr_ = 365 nm, 5 °C, 5 min) yields *Z*_ms_-**1 ⊂ 4·**(BArF)_8_ from *E*_s_-**1 ⊂ 4·**(BArF)_8_ (Figures S31 and S32). Finally, the second THI regenerates *Z*_s_-**1 ⊂ 4** (BArF)_8_ as a result of the thermal relaxation of *Z*_ms_-**1 ⊂ 4**·(BArF)_8_ (Figure S29). The isomeric distribution of **1 ⊂ 4·**(BArF)_8_ is comparable to the one of bulk **1** during the motor rotation cycle (see Table S1 for the full isomeric distribution). This indicates that the cage does not hinder in any way the mechanical function of molecular motor **1**. The spectra of the different isomers of **1** in bulk and confined space of **4·**(BArF)_8_ were compared (Figures S34–S36) and, in all cases, the shielding effect on the motor signals induced by the cage confinement was observed. Fatigue of the host guest system was not observed, as a second rotation cycle in confinement proceeded as expected (Figure S37). Furthermore, continuous irradiation (16 h; Figure S38) did not induce degradation, indicating that the cage-motor complex is robust and can be operated over multiple cycles. ^1^H DOSY NMR data of the host–guest adducts exhibited a clear correlation with the dimensions of empty **4·**(BArF)_8_ in all cases (Figure S40). These results are consistent with internal host–guest complexation.^90,91^ This was further confirmed by restrained MD simulations for the four isomers of *Z*_s_-**1** bound within the cavity (see SI Methods and Figure S12). All isomers of **1** exhibited similar behavior in terms of orientations and host–guest interactions, indicating that all four isomers remain within the cavity despite the drastic geometrical changes of **1**.

The photoisomerization of motor **1** within the confined space of **4·**(BArF)_8_ was also studied by CD spectroscopy. As expected, the achiral **4·**(BArF)_8_ does not display CD signals (Figures 4c and S35). Upon encapsulation of (*R*)-(*M*)-*Z*_s_ **-1** or (*S*)-(*P*)-*Z*_s_-**1**, CD spectra identical to the ones of the motor in bulk solution were obtained with the main Cotton effect at 275 nm. Upon irradiation with 365 nm light at 20 °C, this Cotton effect showed sign inversion, identical with the behavior of the motor in solution (Figure 4d,e). This observation is consistent with the preservation of the motor characteristics upon encapsulation within **4·**(BArF)_8_.

Metal–organic cage **4·**(BArF)_8_ does not interfere with motor rotation in the host–guest complex. Interestingly, the influence of confined space on the operation of the molecular motor in **1** is very small, in line with the low occupancy of the cavity (vide supra). Indeed, almost no changes in the photochemical steps and no significant differences are observed in the thermal processes with respect to the motor in bulk solution (Figure S33), unlike the generally observed alteration of molecular motor properties upon confinement within 2D or 3D solid materials.^47,59^

## Conclusions

We have shown that the rotation of a molecular motor can occur in the confined space of a MOC. Molecular host **4**·(BArF)_8_ enables the accommodation of rotary molecular motor **1** (comprising an alkyl chain with a terminal COOH anchoring group) within its cavity. 1D and 2D NMR analysis, together with MD simulations, allowed us to conclude that the host–guest confinement is essentially driven by the H-bonding between the guest alkyl-COOH tail, and the carbonyl groups residues present in the cavity of **4**·(BArF)_8_ instead of a traditional size-fit interaction. Due to our host–guest design that allows for sufficient cavity free volume, the confined molecular motor is able to perform a 360° unidirectional rotation cycle without exiting the host and without noticeable alteration of its rotation properties. By using ^1^H NMR and CD spectroscopy, the light-driven rotary motion in confined space could be directly compared to that in solution. This design is particularly attractive as a proof of concept, to be expanded to different types of molecular motors with a simple modification of their structure by functionalization with an alkyl-COOH chain. This will allow, in principle, the accommodation of different motor guests into a nanocage without the restrictions imposed by a size-fit dependence.

New generations of motor-cage host–guest systems can be developed based on these design principles, for example, energy transfer-enabling cages, multiple installation of motor anchoring points, and exploration of different motor and cage sizes, to mention a few. Understanding these phenomena could enable the development of systems with unique mechanical functions mimicking, for instance, nature’s DNA packing motors.

## Supplementary Material





## Notes

Materials, instrumentation, experimental procedures, spectroscopic and photophysical characterization of all compounds; characterization of the host–guest adducts; molecular dynamics studies; and in-situ NMR experiments (PDF)
MD simulation movie (AVI)
