# Supplementary material for: Light-Induced Rotation of a Molecular Motor in the Confined Space of a Metal–Organic Nanocage
Source: J Am Chem Soc. 2026 Jan 26;148(4):4189–97. doi: 10.1021/jacs.5c16349 (PMC12879731; doi:10.1021/jacs.5c16349)
Supplement: Supplementary file 1 [file ja5c16349_si_001.pdf]

Supporting information for

Light-Induced Rotation of a Molecular Motor in the Confined Space of a Metal-Organic Nanocage

Carles Fuertes-Espinosa,[1¥] Marco Ovalle,[2¥] Yohan Gisbert,[2] Clara Sabrià,[1] Valentina Iannace, [1] Josep M. Luis,[1] Ferran Feixas,[1] Alexander Ryabchun,[2] Xavi Ribas[1]* and Ben L. Feringa[2]*

[1]Institut de Química Computacional i Catàlisi (IQCC) and Departament de Química, Universitat de Girona, Campus Montilivi, 17003 Girona, Catalonia, Spain

[2]Stratingh Institute for Chemistry, University of Groningen, Groningen, 9747AG, The Netherlands

[¥] These authors contributed equally.

I. Supplementary experimental procedures 2
1. General methods 2
2. Synthetic procedures 3
II. Characterization of the host-guest adducts 5
III. Control experiments with Z_s-2 10
IV. Molecular Dynamics studies 11
V. *In-situ* NMR experiments in bulk solution. 16
VI. CD spectroscopy of motor in bulk solution 24
VII. NMR experiments in confined space. 26
VIII. CD spectroscopy of Motor in Confined Space 38
IX. HPLC chromatograms 40
X. Computational assignment of stereochemistry 42
XI. NMR spectra of new compounds 45
XII. References 49

I. Supplementary experimental procedures

1. General methods

Commercial reagents and solvents: All chemicals and solvents were purchased from commercial suppliers unless otherwise stated. Anhydrous solvents were obtained using a MBraun SPS 800 system and stored under N_2.

Synthesized reagents: Molecular motor **2** (2-Methoxy-9-(2',3'-dihydro-2'-methyl-1'*H*-naphtho[2,1-*b*]thiopyran-1'-ylidene)-9*H*-thioxanthene)[1,2] and **4**·$(BArF)_8$[3] were synthesized according to reported literature procedures and characterized using routine characterization techniques.

Synthesis and purification: Standard Schlenk techniques were used, employing nitrogen or argon as the inert gas. If they were not performed at room temperature, the reaction temperatures refer to the temperature of the heating/cooling bath or heating block.

Flash column chromatography was performed on a Biotage Selekt system using the indicated solvents. TLC analysis was done on Merck silica gel 60 F_{254} aluminum sheets, and compounds were visualized with a UV lamp (254 nm or 365 nm).

Analysis:

NMR:

Full characterization of the newly synthesized compounds was performed using a Bruker Avance Neo 600 (600 MHz) or an Agilent MR (400 MHz) spectrometer. NMR spectra for routine ^{1}H NMR, ^{1}H DOSY and host-guest ^{1}H NMR titration were recorded using a Bruker 400 MHz Avance III HD Smart Probe. Chemical shifts (δ) are given in parts per million (ppm) relative to TMS, using the solvent residual peak as internal standard ($CDCl_3$: δ = 7.26 for ^{1}H, δ = 77.16 for ^{13}C, Acetonitrile-d_3: δ = 1.94 for ^{1}H). Data is reported as follows: chemical shifts (δ) in ppm, multiplicity (s = singlet, d = doublet, dd = doublet of doublets, ddd = doublet of doublets of doublets, t = triplet, q = quartet, m = multiplet), coupling constants *J* (Hz), and integration. Signals were assigned with the help of 2D NMR experiments. Variable temperature NMR and *in-situ* irradiation experiments were performed using a Varian Inova 500 (500 MHz) spectrometer. NMR irradiation experiments were performed at the indicated temperature with a fiber-coupled LED and a 1000 μm optical fiber (FT1000URT) to guide the light directly into the NMR tube inside the NMR spectrometer. *Ex-situ* irradiation was performed in J Young NMR tubes. The samples were degassed by three freeze-thaw cycles.

High-resolution mass (HMRS) spectra were recorded on a Thermofisher LTQ Orbitrap XL or Bruker MicroTOF-Q-II

HPLC analysis and separation was performed using a Shimadzu SPD M10AVP diode array detector using Chiralpak IB analytical and semi-preparative columns with mixtures of HPLC-grade *n*-heptane and isopropanol as the eluent (90:10, 1 mL/min for analytical and 80:20, 4.5 mL/min for semi-preparative) with a column temperature of 40 °C.

UV/Vis absorption spectra were recorded on a Agilent Cary 8454 spectrophotometer in 1 cm quartz cuvettes. The LEDs were attached via a 1500 μm optical fiber (M93L01). **CD** spectra were recorded on a Jasco J-815 spectropolarimeter.

Irradiation experiments were performed using fiber-coupled LEDs (M365F1) powered with a T-Cube™ LEDD1B driver obtained from Thorlabs Inc.

2. Synthetic procedures

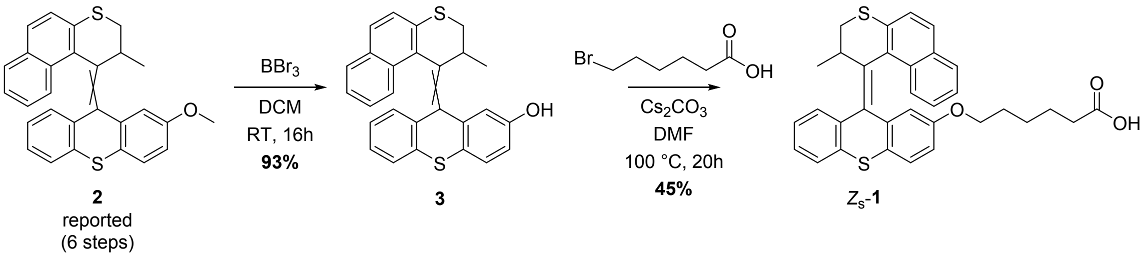

Figure S1. Synthesis of molecular motor **1**.

2-Hydroxy-9-(2‘,3‘-dihydro-2‘-methyl-1‘*H*-naphtho[2,1-*b*]thiopyran-1‘-ylidene)-9*H*-thioxanthene (3)

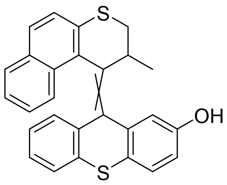

2-Methoxy-9-(2‘,3‘-dihydro-2‘-methyl-1‘*H*-naphtho[2,1-*b*]thiopyran-1‘-ylidene)-9*H*-thioxanthene **2** (300 mg, 0.68 mmol, 1 equiv.) was dissolved in anhydrous CH_2Cl_2 (7 ml). BBr_3 (1M in CH_2Cl_2, 1.5 mL, 1.45 mmol, 2.1 equiv.) was added dropwise and the solution was stirred at room temperature for 16 hours under a nitrogen atmosphere. The mixture was slowly poured into a saturated $NaHCO_3$ solution (10 mL). The organic layer was diluted with an additional 10 mL of DCM, separated, washed with water (10 mL) and brine (10 mL). The resulting solution was dried over anhydrous $MgSO_4$ and the solvent was removed in vacuo. The crude residue was then purified by column chromatography (SiO_2, EtOAc) to afford 2-hydroxy-9-(2‘,3‘-dihydro-2‘-methyl-1‘*H*-naphtho[2,1-*b*]thiopyran-1‘-ylidene)-9*H*-thioxanthene **3** composed of a ~1:1 mixture of *E*- and *Z*- isomers as a white solid in 93% yield (269 mg, 0.63 mmol).

^{1}H NMR (600 MHz, $CDCl_3$, 25 °C): δ = 7.65 – 7.53 (m, 8H), 7.47 (d, *J* = 8.4 Hz, 1H), 7.38 (dt, *J* = 8.4, 1.1 Hz, 2H), 7.37 – 7.33 (m, 1H), 7.28 (t, *J* = 8.0 Hz, 2H), 7.17 – 7.09 (m, 4H), 7.07 – 7.02 (m, 1H), 7.00 (ddd, *J* = 8.3, 6.7, 1.3 Hz, 1H), 6.77 (dd, *J* = 8.4, 2.6 Hz, 1H), 6.74 – 6.70 (m, 1H), 6.43 – 6.36 (m, 2H), 6.26 (dd, *J* = 8.4, 2.7 Hz, 1H), 5.87 (d, *J* = 2.8 Hz, 1H), 4.23 – 4.17 (m, 1H), 4.14 – 4.08 (m, 1H), 3.73 – 3.66 (m, 2H), 3.10 – 3.03 (m, 2H), 0.80 (d, J = 6.9 Hz, 3H), 0.79 (d, J = 7.0 Hz, 3H) ppm. **^{13}C{^{1}H} NMR** (151 MHz, $CDCl_3$, 25 °C): 154.4, 153.8, 140.1, 138.4, 138.1, 136.9, 136.7, 136.1, 135.2, 135.0, 135.0, 132.6, 132.5, 131.6, 131.6, 131.5, 131.5, 131.0, 130.9, 129.1, 129.0, 127.9, 127.8, 127.7, 127.7, 127.7, 127.6, 126.9, 126.6, 126.2, 126.1, 126.0, 125.9, 125.6, 125.6, 125.4, 124.7, 124.6, 124.5, 115.9, 114.9, 114.3, 113.9, 37.3, 37.3, 32.6, 32.4, 19.4, 19.4 ppm. **HR-MS** (ESI+): calcd. for $C_{27}H_{19}OS_2$ [M-H]$^-$: 423.08828, found 423.08755.

For ^{1}H NMR, *E*- and *Z*- isomers were integrated separately, resulting in an overall number of protons being double of the one expected for the compound. In ^{13}C NMR, some signals of *E*- and *Z*- isomers are overlapping, resulting in an overall number of signals smaller than two times the number of carbons.

Z_s-**1**

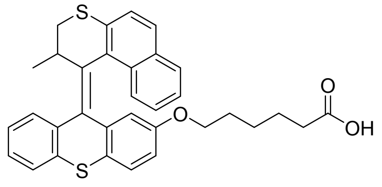

2-Hydroxy-9-(2‘,3‘-dihydro-2‘-methyl-1‘*H*-naphtho[2,1-*b*]thiopyran-1‘-ylidene)-9*H*-thioxanthene **3** (269 mg, 0.63 mmol, 1 equiv.), 6-bromohexanoic acid (247 mg, 1.27 mg, 2 equiv.) and Cs_2CO_3 (826 mg, 2.53 mmol, 4 equiv.) were placed in a vial equipped with a magnetic stir bar. Anhydrous *N*,*N*-dimethylformamide (20 mL) was added, the vial was sealed, heated to 100 °C and stirred at this temperature for 20 hours. The solvent was removed in vacuo yielding a dry residue which was acidified with 1M aqueous HCl (75 mL, verifying that pH is acidic). CH_2Cl_2 (75 ml) was added, the organic layer was washed with brine (75 mL) and dried over anhydrous $MgSO_4$. The solvent was removed in vacuo and the crude residue purified by flash column chromatography (SiO_2, EtOAc/Pentane 0:100 to 7:93) to afford pure Z_s-**1** as a white solid in 45% yield (120 mg, 283 mmol).

^{1}H NMR (400 MHz, $CDCl_3$, 25 °C): δ = 7.64 – 7.56 (m, 5H), 7.41 – 7.32 (m, 2H), 7.30 – 7.25 (m, 1H), 7.14 (t, *J* = 7.9 Hz, 2H), 7.04 (ddd, *J* = 8.5, 6.8, 1.5 Hz, 1H), 6.25 (dd, *J* = 8.4, 2.7 Hz, 1H), 5.87 (d, *J* = 2.7 Hz, 1H), 4.15 (dd, *J* = 6.1, 4.6 Hz, 2H), 4.14 – 4.08 (m, 1H), 3.70 (dd, *J* = 11.4, 7.4 Hz, 1H), 3.07 (dd, *J* = 11.4, 3.2 Hz, 1H), 2.40 – 2.34 (m, 2H), 1.71 – 1.59 (m, 4H), 1.51 – 1.40 (m, 2H), 0.78 (d, *J* = 6.7 Hz, 3H) ppm. **^{13}C{^{1}H} NMR** (101 MHz, $CDCl_3$, 25 °C): δ = 173.8, 153.9, 140.1, 136.9, 136.6, 136.2, 135.0, 132.5, 131.6, 131.5, 131.0, 127.9, 127.7, 127.7, 127.7, 127.8, 126.9, 126.2, 126.1, 125.7, 125.6, 124.6, 124.6, 115.9, 113.9, 63.3, 37.3, 34.7, 32.4, 28.2, 24.7, 24.7, 19.3 ppm. **HR-MS** (ESI+): calcd. for $C_{33}H_{31}O_3S_2$ $[M+H]^+$: 539.17092, found 539.17028. **HPLC** (CHIRALCEL IB, ^{i}PrOH/^{n}heptane 10:90, 1 mL/min, 40 °C, 256 nm): retention times (min) 6.76 (*R*)-Z_s-**1** , 9.87 **(*S*)**-Z_s-**1**.

II. Characterization of the host-guest adducts

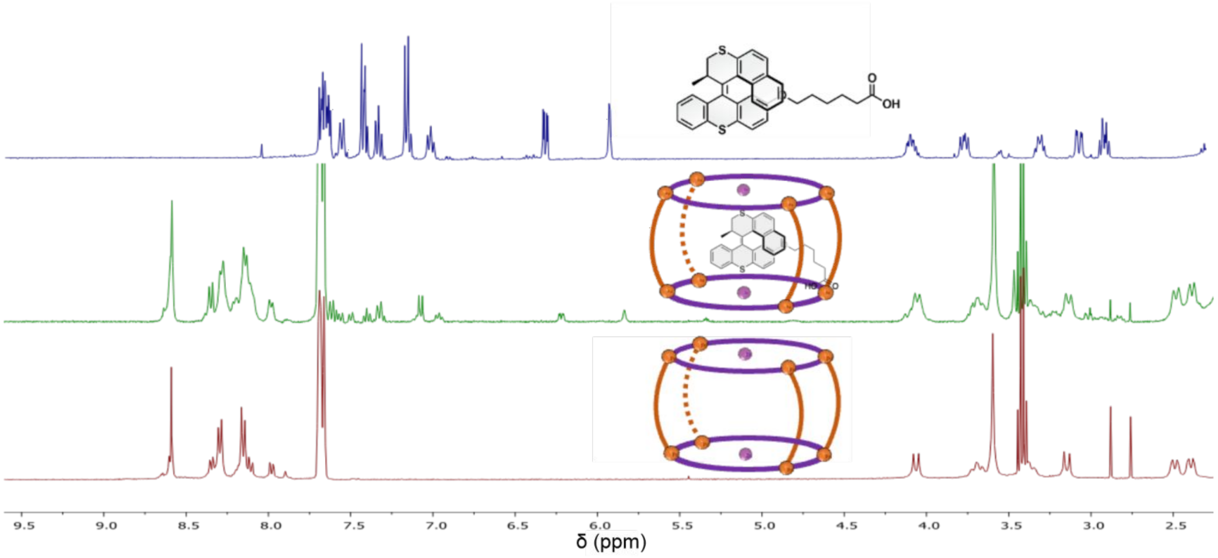

Figure S2. Partial ^{1}H NMR spectra (400 MHz, CD_3CN, 298 K) of **4**·$(BArF)_8$ (bottom), the prepared Z_s-**1** ⊂ **4**·$(BArF)_8$ host-guest complex (middle) and Z_s-**1** (top).

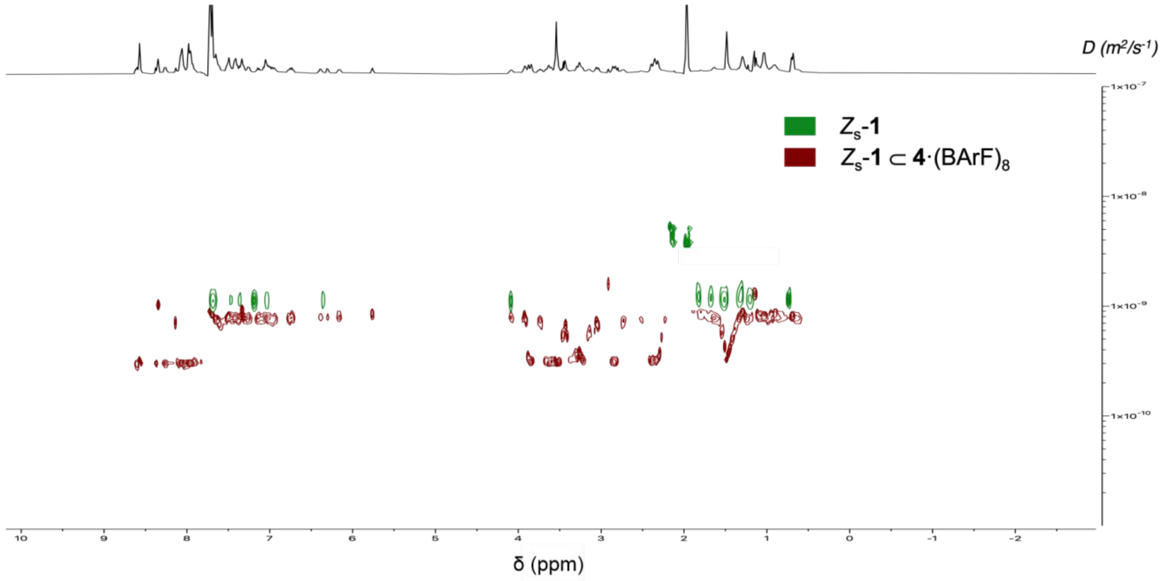

Figure S3. Overlaid ^{1}H DOSY spectra (400 MHz, CD_3CN, 298 K) of the prepared Z_s-**1** ⊂ **4**·$(BArF)_8$ host-guest complex (red) and the Z_s-**1** (green). The diffusion coefficients for the host and the guest species in CD_3CN were measured to be 3.1×10^{-10} m^2 s^{-1} and 8.1×10^{-10} m^2 s^{-1} respectively. The diffusion coefficient for free Z_s-**1** in CD_3CN was measured to be 2.1×10^{-9} m^2 s^{-1}. The differences observed in the diffusion coefficients of the host and the guest in the Z_s-**1** ⊂ **4**·$(BArF)_8$ host-guest complex are indicative of fast exchange binding dynamics on the NMR time scale.

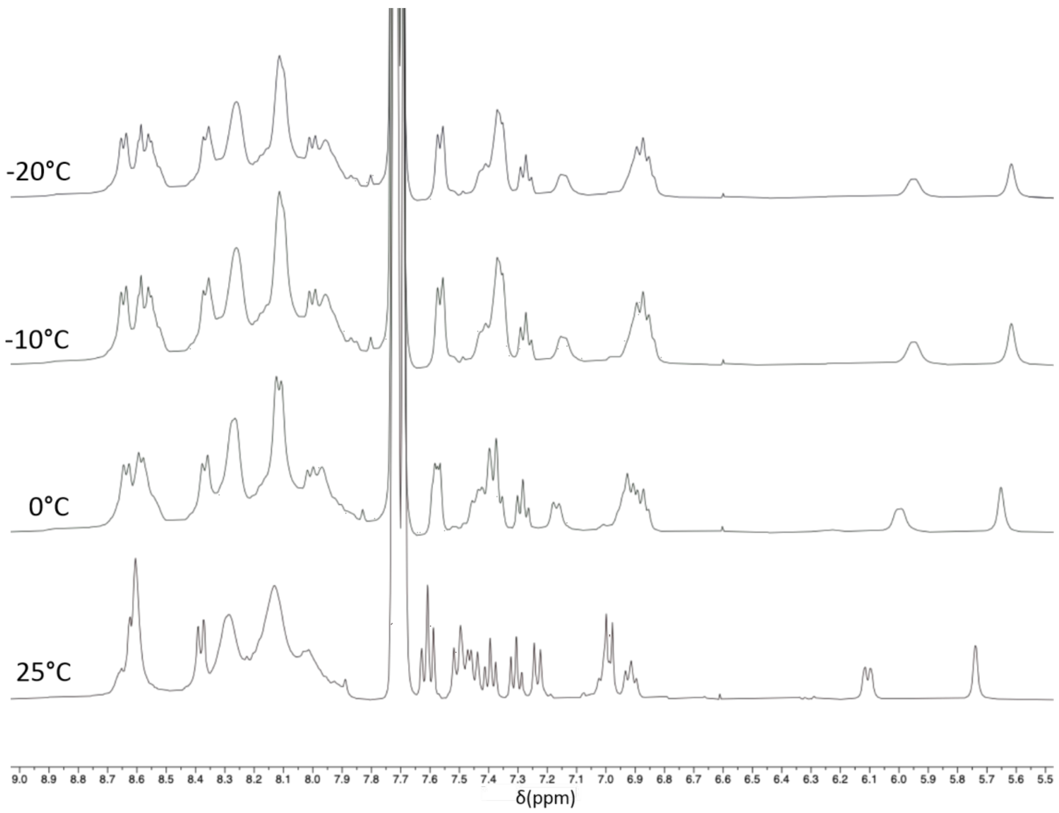

Figure S4. Stacked ^{1}H NMR spectra (400 MHz, CD_3CN) of Z_s-**1** $\subset$ **4**·$(BArF)_8$ host-guest complex, recorded at different temperatures.

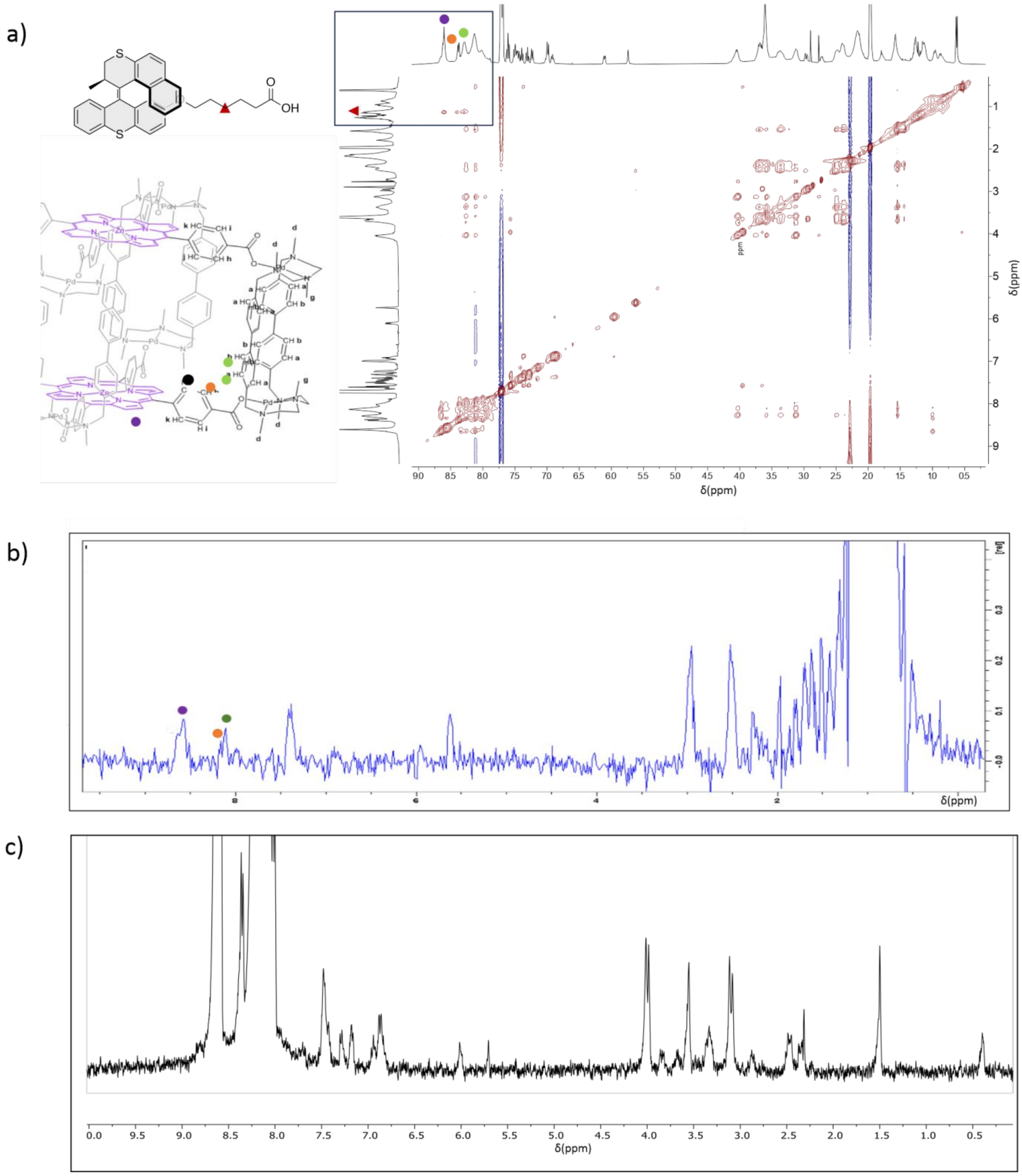

Figure S5. *One and two-dimensional NOESY spectra (400 MHz, CD_3CN, 298 K) of Z_s-1 ⊂ 4·(BArF)$_8$ host-guest complex. Illustration of different 4·(BArF)$_8$ protons showing NOESY correlations between the aliphatic protons from the alkyl site chain present in Z_s-1 and the aromatic protons of 4·(BArF)$_8$ near by the carbonyl groups present in the host cavity. a) ^1H-^1H NOESY spectrum at 298 K and (b) 1D selective gradient NOESY spectrum (irradiated at 1.05 ppm) at 263 K. (c) 1D selective gradient NOESY spectrum (irradiated at 8,1204 ppm) at 263 K, showing correlations between the protons of the cage pointing inwards the cavity and the aromatic protons of Z_s-1.*

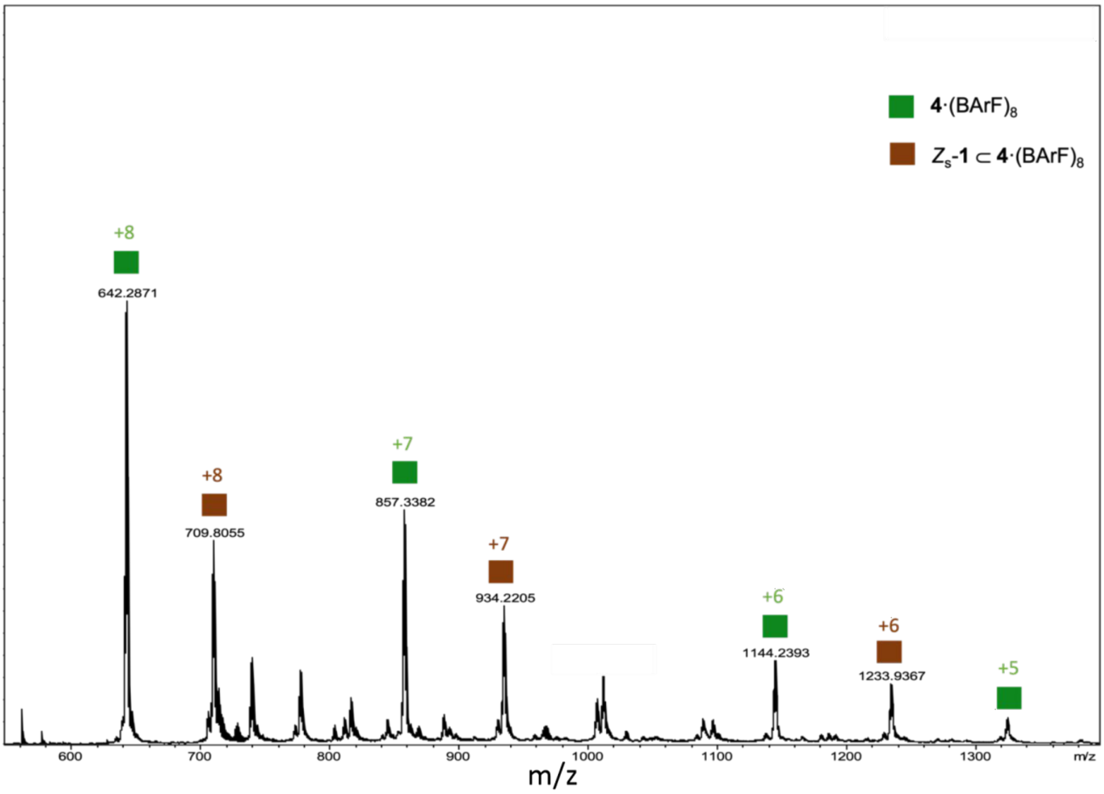

Figure S6. High Resolution Mass Spectrometry (HRMS) spectrum of Z_s-**1** ⊂ **4**·(BArF)$_8$ host-guest complex (CH_3CN solvent).

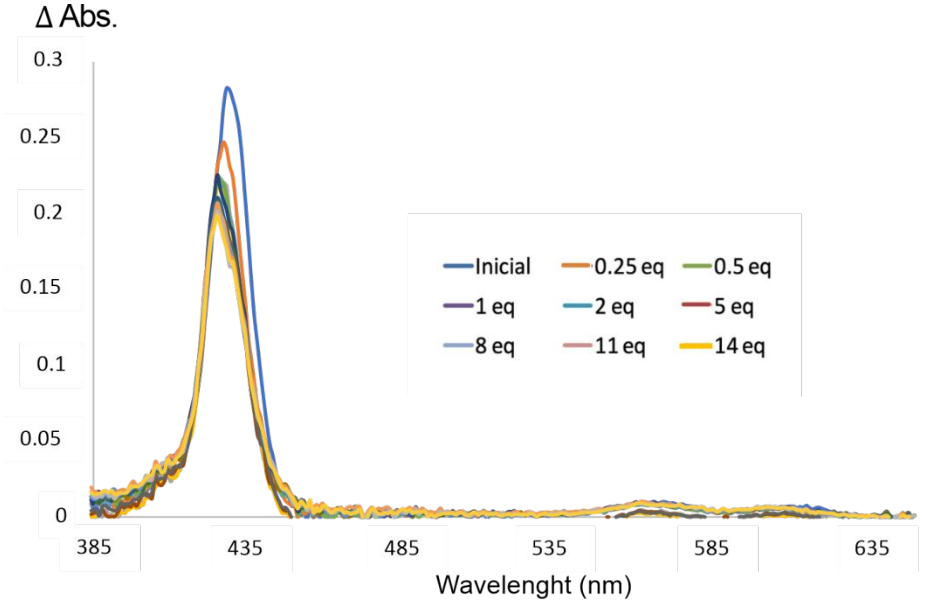

Figure S7. UV-Vis monitoring for the titration of **4**·(BArF)$_8$ with Z_s-**1**. Fixed total concentration of **4**·(BArF)$_8$ in acetonitrile was measured to be $4.32 \cdot 10^{-7}$ M (fitting with www.supramolecular.org, $K_a = 2.3\ (\pm 0.5) \cdot 10^5\ M^{-1}$).

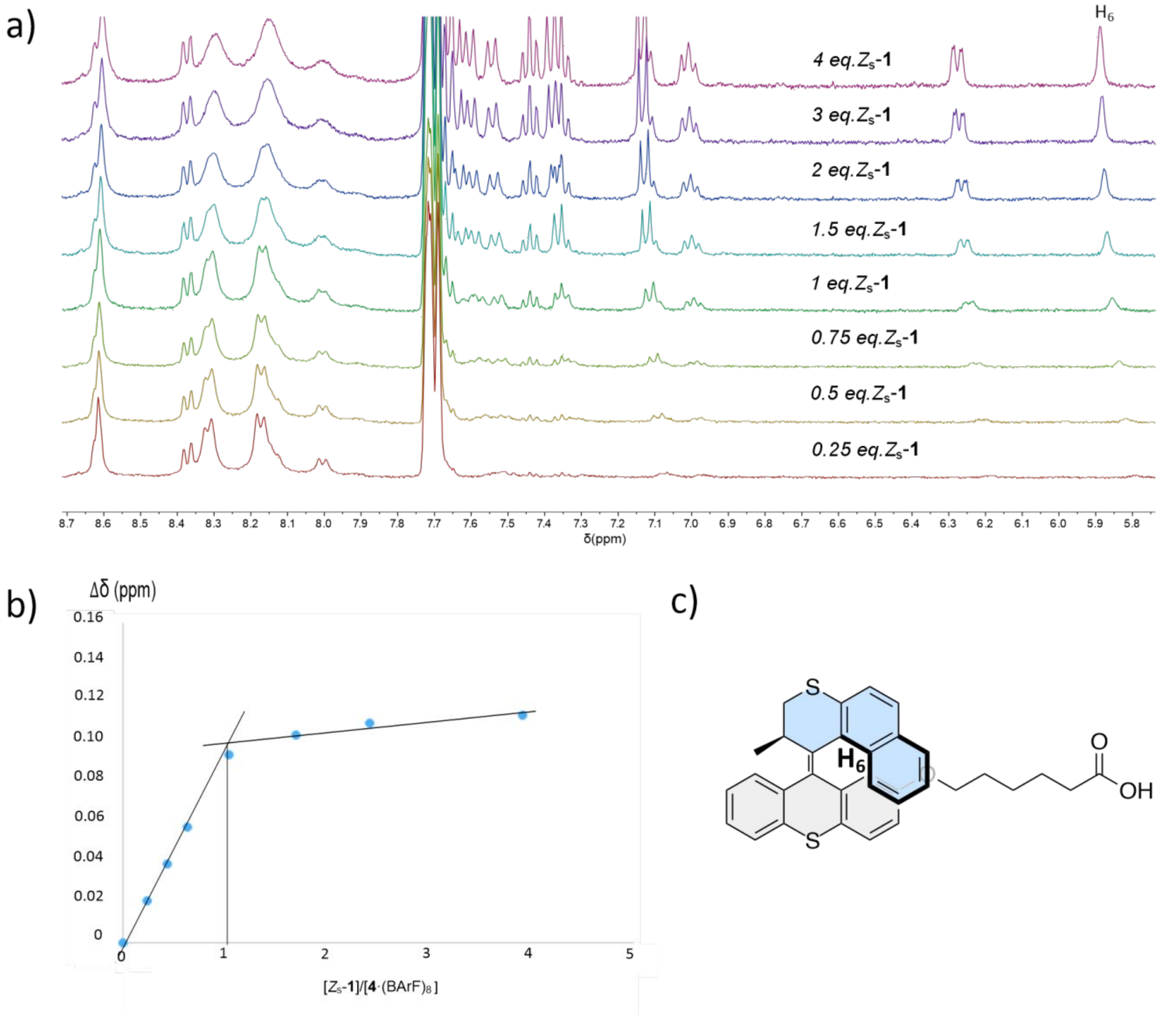

Figure S8. a) Partial ^{1}H NMR spectra (400 MHz, CD_3CN, 298 K) of the titration of **4**·$(BArF)_8$ with Z_s-**1**, showing the chemical shifts of the guest shifting toward lower field upon increasing molar fraction of guest in solution. b) ^{1}H-NMR stoichiometry plot for the titration of **4**·$(BArF)_8$ with Z_s-**1**. The plot refers to the chemical shift of H_6 of Z_s-**1**. A series of 4 mM solutions of **4**·$(BArF)_8$ containing varying concentrations of Z_s-**1**, ranging from 2.06 mM to 66 mM, was prepared. The solutions were allowed to equilibrate before they were examined by ^{1}H-NMR spectroscopy. The stoichiometry was found to be one guest molecules per one host molecule using the mole ratio method.[4]

III. Control experiments with Z_s-2

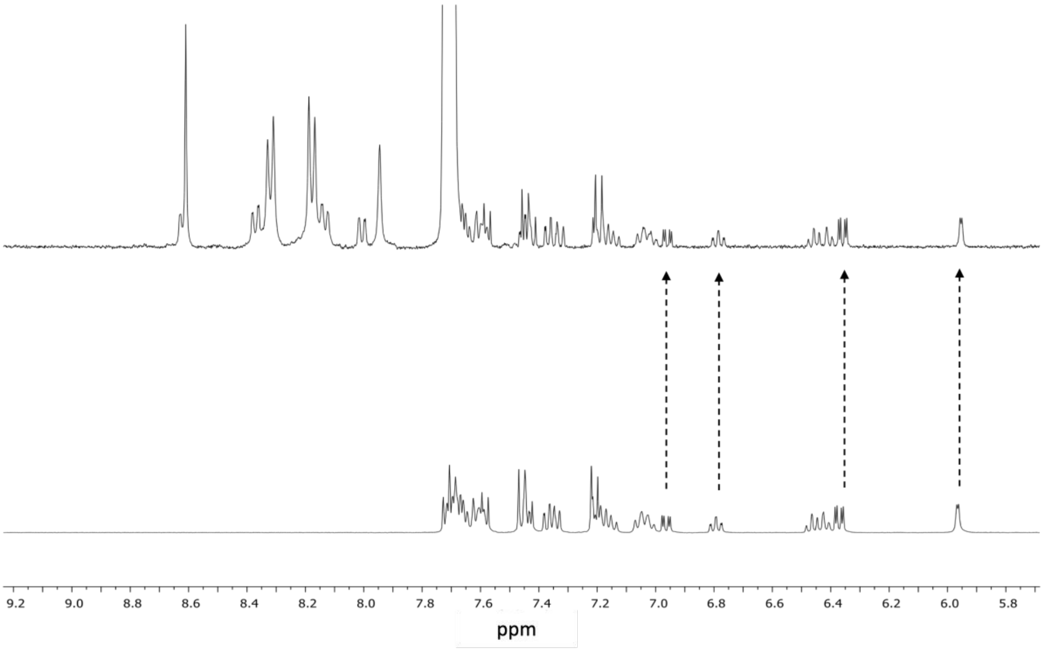

Figure S9. Partial ^{1}H NMR spectra (400 MHz, CD_3CN, 298 K) of Z_s-**2** (bottom) and Z_s-**2** in the presence of **4**·(BArF)$_8$ (top). No evidence of host-guest interactions was found.

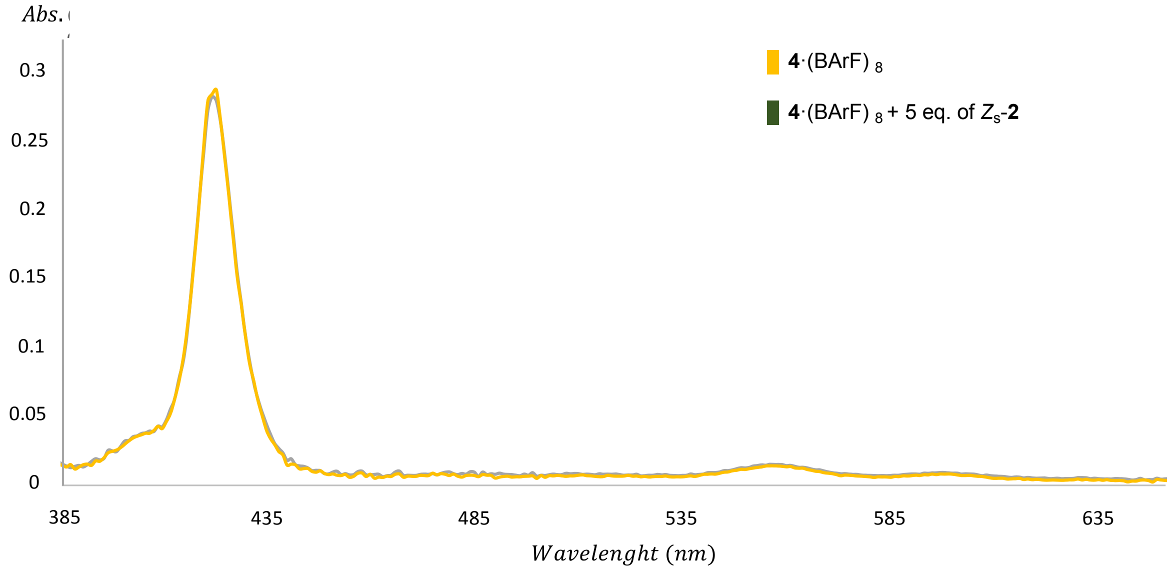

Figure S10. UV-Vis monitoring for the titration of **4**·(BArF)$_8$ with Z_s-**2**. Fixed total concentration of **4**·(BArF)$_8$ in acetonitrile was measured to be $4.32 \cdot 10^{-7}$ M. No absorbance variation at 432 nm (Soret band from **4**·(BArF)$_8$) were detected upon the addition of different equivalents of Z_s-**2** (up to five equivalents).

IV. Molecular Dynamics studies

System preparation and MD protocols

The cage **4**·$(Cl)_8$ was parametrized following a similar procedure reported in our previous work.[5] The parameters were obtained with a protocol combining the General Amber Force Field (GAFF)[6] and the Metal Center Parameter Builder (MCPB.py)[7] tools, with crystallographic X-ray data as the starting structure of the cage. The cage was divided in different fragments to obtain the parameters independently. Regarding the fragments that do not contain metals, as the macrocyclic clips, the parameters were obtained using GAFF. The optimization and the partial charges were obtained at the B3LYP/6-31G* level of theory using the restricted-electrostatic potential (RESP) charges.[8] The charges were calculated using the Merz-Singh-Kollman method with the Gaussian 16 package.[9] For the fragments containing metal centers (Pd(II) and Zn(II)) were parametrized using the MCPB.py and the antechamber module of AMBER 22 package.[10] The RESP atomic charges of metal-based fragments were obtained from B3LYP/6-31G* (SDD basis function for Pd) calculations while bonding, angle, and torsion parameters were obtained from frequency calculations using the Seminario method implemented in MCPB.py. Finally, all the fragments were put together based on the reported crystallographic structure.

Molecular dynamics (MD) simulations were carried out with acetonitrile (MeCN) as solvent and the GPU code (pmemd)[11] of the AMBER 18 package. As the starting point for the simulations, the motor (**1** or **2**) was placed inside the cage. The host-guest complex was immersed in a preequilibrated truncated octahedron box with a 10 Å buffer of MeCN molecules, using the leap module of the AMBER package, and neutralized by adding eight explicit counterions (Cl^-). Each complex was submitted to a two-stage geometry optimization approach. The first stage corresponds to a minimization of the positions of solvent molecules and ions imposing positional restraints on the solute by harmonic potential with a force constant of 500 kcal mol^{-1} $Å^{-2}$. The second stage corresponds to an unrestrained minimization of all the atoms in the simulation cell. Then, the system was gently heated using six 50 ps steps, where the temperature is increased by 50 K each step (0-300 K) under constant volume and periodic boundary conditions. The SHAKE algorithm was employed to constraint the covalent bonds involving hydrogens. Long-range electrostatic effects were modelled using the particle-mesh Ewald method.[12] An 8 Å cutoff was applied to Lennard-Jones and electrostatic interactions. Harmonic restraints of 10 kcal.mol^{-1} were applied to the solute and the Langevin equilibration scheme was used to control and equalize the temperature. The time step was maintained at 2 fs during the heating steps, allowing potential in homogeneities to self-adjust. Finally, each system was equilibrated without restrains for 2 ns with a 2 fs time step at a constant pressure and temperature of 300 K. 2 replicates of 1 µs have been carried out for the system with rotor Z_s-**1** and rotor Z_s-**2**, while 2 replicates of 0.5 µs have been performed for the other rotors (E_{ms}-**1**, Z_{ms}-**1** and E_s-**1**).

Initially, unconstrained MD simulations were conducted starting from a pre-bound state, where the motor was already positioned inside the cavity of the cage, as the encapsulation is beyond the accessible simulation timescales. During these simulations, a spontaneous formation of a hydrogen bond was observed between the carboxylic acid group of the rotor and one carboxylate oxygen of the host, effectively anchoring the rotor inside the cavity. The cage contains eight equivalent carboxylate groups linking the porphyrin moieties to the lateral clips via monodentate coordination to palladium through one of the two oxygen atoms. This mode of coordination leaves the second oxygen atom of each carboxylate group unbound and available as a potential hydrogen bond acceptor. In the crystal structure, these free oxygens are oriented toward the solvent, but rotational flexibility of the carboxylate groups allows for transient reorientation where the free oxygen atoms can point toward the interior of the cavity. This is what happens when the formation of the hydrogen bond is observed in the initial simulations, a single carboxylate oxygen rotates toward the interior of the cavity to establish

the interaction with the rotor. However, since the carboxylate group reorientation is a relatively slow process, such rotation events are rare within typical MD timescales.

For this reason, restrained simulations were carried out to systematically explore the behavior of the system in the presence of this interaction. A soft distance restraint between the hydrogen of the motor's carboxylic acid and the oxygen of a selected carboxylate group of the cage was applied. This restraint was only applied when the distance exceeded 6 Å and was designed to keep a physically realistic range (1.5-4.5Å). This restraint does not artificially enforce a hydrogen bond interaction; rather, it allows to study the structural consequences of such an interaction once it has formed, under conditions where the natural carboxylate rotation might otherwise be too infrequent to sample. During the simulations, the distance remained well within hydrogen-bonding range, suggesting that the interaction is both favorable and stable once established.

Find the MD simulation video as the uploaded Video S1 (VideoS1_Zs-1@Cage.mpeg).

Molovol has been used to calculate the volume.[13] The molecular volume of the motors (**1** and **2**) was calculated with the single-probe mode, using 1.2 Å as the small probe radius, 0.2 Å as grid resolution and 4 as optimization depth. This molecular volume ($V_{mol} = V_{vdw} + V_{void}$) corresponds to the effective volume occupied by the motor. The volume of the cavity of the cage can be calculated with the two-probe mode, with a small probe radius of 1.2 Å, a large probe radius of 5 Å, a grid resolution of 0.2 Å and an optimization depth of 4. Since the windows of this cage are quite large, we have not been able to obtain a cavity without considering also the windows. So, it was hypothesized that the cavity's volume of the cage was being overestimated. For this reason, the final volume of the cavity was calculated by defining a tetragonal prism from the internal cavity, limiting the vertexes of the prism on the oxygen atoms that are binding the palladium centers. The volumes were visualized using Pymol.

The non-covalent interaction (NCI) volume was calculated between the cage **4**·(Cl)$_8$ and the motor Z_s-**1** for three frames where the motor is in different orientations (Fig. S14).[14] The calculation was performed without taking into account the solvent molecules. The NCI was visualized using Pymol. A rainbow scale was used, where red represents repulsive interactions, blue represents attractive interactions, and green shows weak van der Waals interactions.

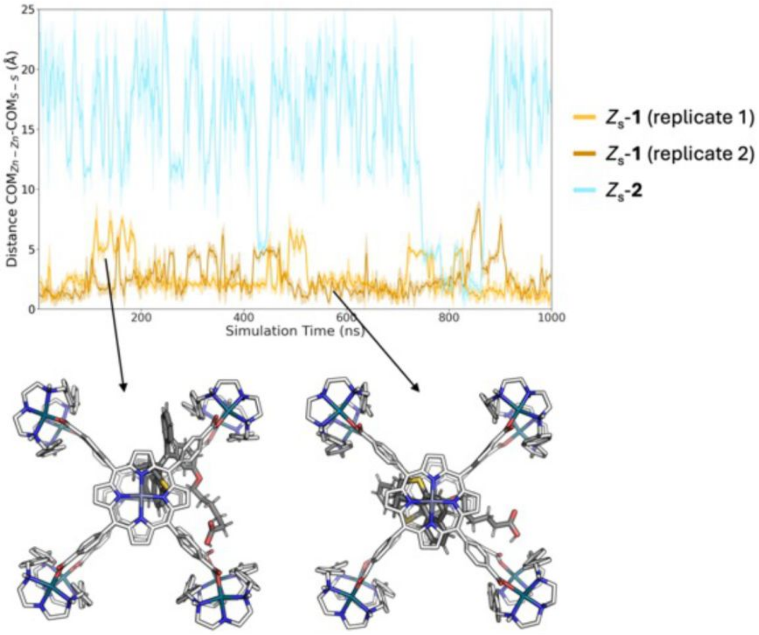

Figure S11. Representation of the distance (Å) between the center of masses of the two Zn atoms of the cage and the center of masses of the two S atoms of the motor respect the

simulation time (ns). Comparison between the motor Z_s-**1** in yellow (with the carboxylic tail, two replicates) and Z_s-**2** (without the carboxylic tail) in blue. Z_s-**1** shows smaller distances, since it is encapsulated inside the cage thanks to the hydrogen bonding interaction. Z_s-**2** does not show encapsulation the majority of the time (the distance shown in blue is larger). Two frames with different distances are shown, where the orientation of Z_s-**1** inside the cage can be observed.

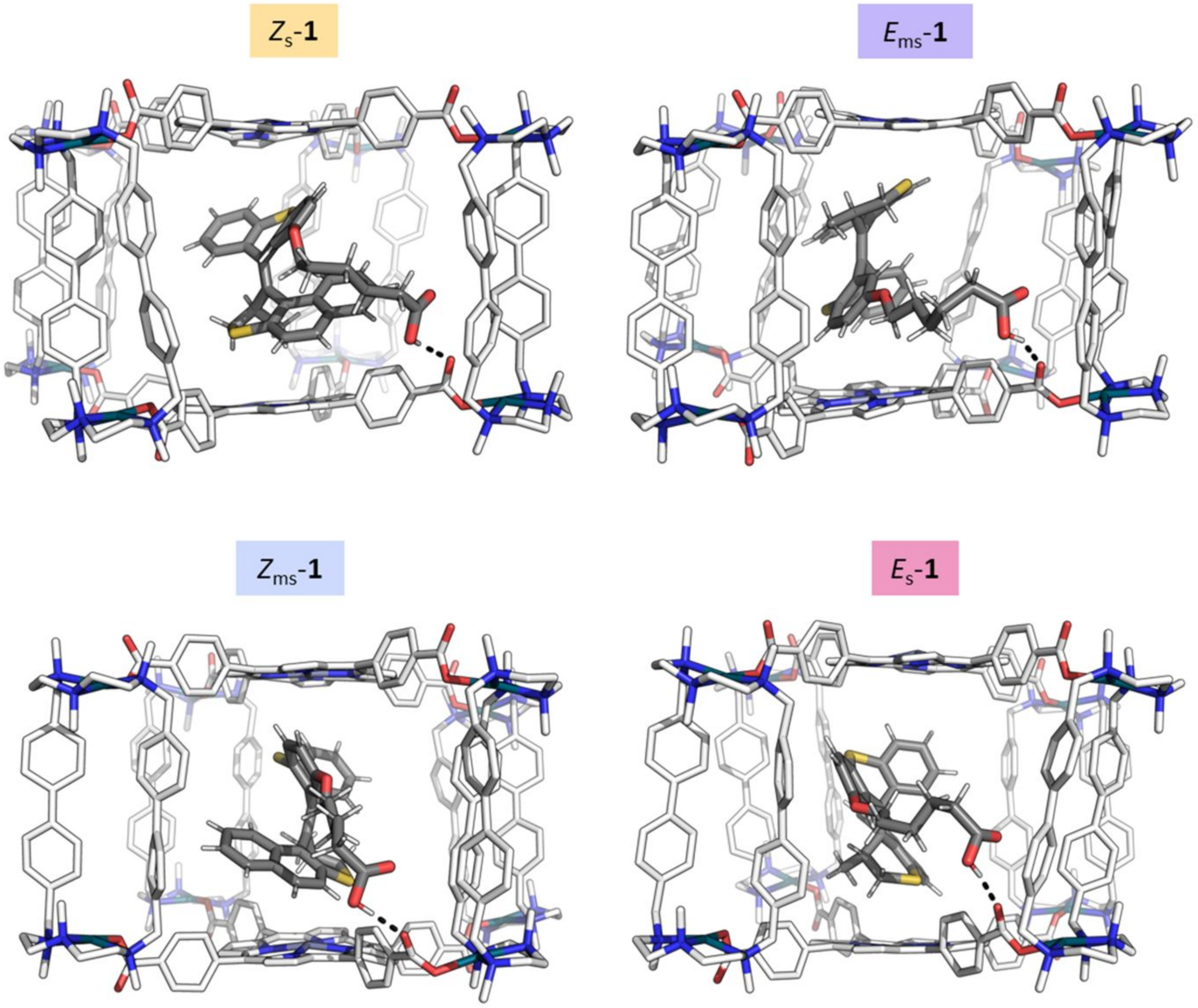

Figure S12. Snapshot of each isomer of motor **1** inside the cage **4**·$(Cl)_8$ taken from the MD simulations. The hydrogen bonding between the carboxylic acid of the motor and the carboxylate of the cage is marked with a dotted line in black. Hydrogens of the nanocage have been omitted for clarity.

a) Calculation of the cavity volume of the nanocage **4**·$(Cl)_8$ with Molovol

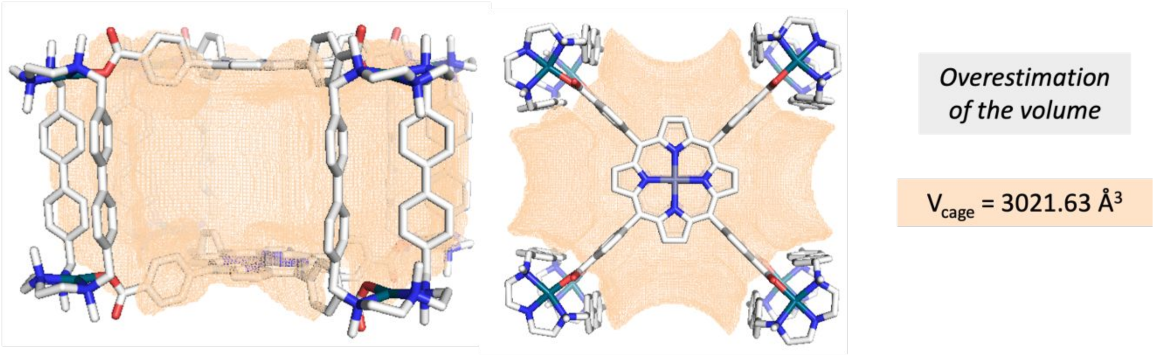

b) Representation of the nanocage **4**·$(Cl)_8$ volume and the molecular volume of the motor *Z*$_s$-**1**

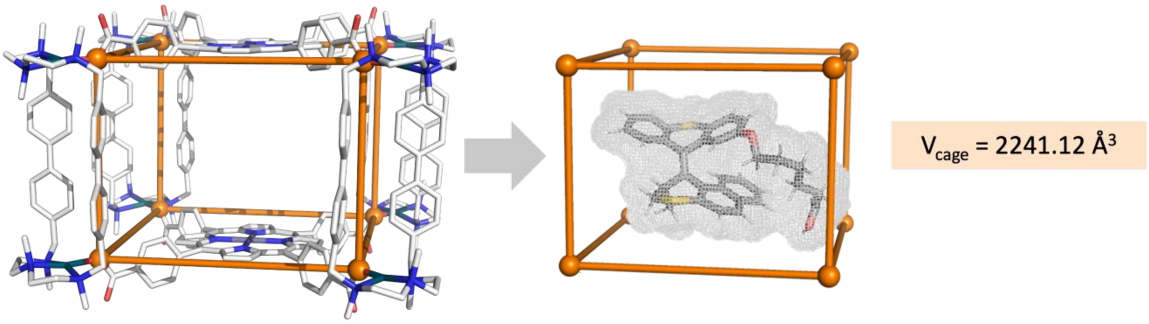

c) Percentage of occupation of each isomer of motor **1** inside the cage **4**·$(Cl)_8$

Motor 1	V_{motor} (Å^3)	% of $V_{occupation}$
Z$_s$-**1**	540.00	24.10
E$_{ms}$-**1**	535.02	23.87
E$_s$-**1**	536.95	23.96
Z$_{ms}$-**1**	536.66	23.95

Figure S13. a) Calculation of the cavity volume of the nanocage **4**·$(Cl)_8$ using Molovol (two-probe mode with small probe radius 1.2 Å, large probe radius 5 Å, grid resolution 0.2 Å and optimization depth 4). Representation of the cavity volume in orange, observing that the windows are also considered, so this volume is being overestimated. Hydrogens of the nanocage have been omitted for clarity. b) Calculation of the cavity volume by limiting a tetragonal prism (represented in orange) from the oxygen atoms bonding the palladium vertexes of the cage. Calculated molecular volume of the motor shown in grey. Hydrogens of the nanocage have been omitted for clarity. c) Table showing the percentage of the cavity volume occupancy of each isomer of motor **1** inside the nanocage **4**·$(Cl)_8$.

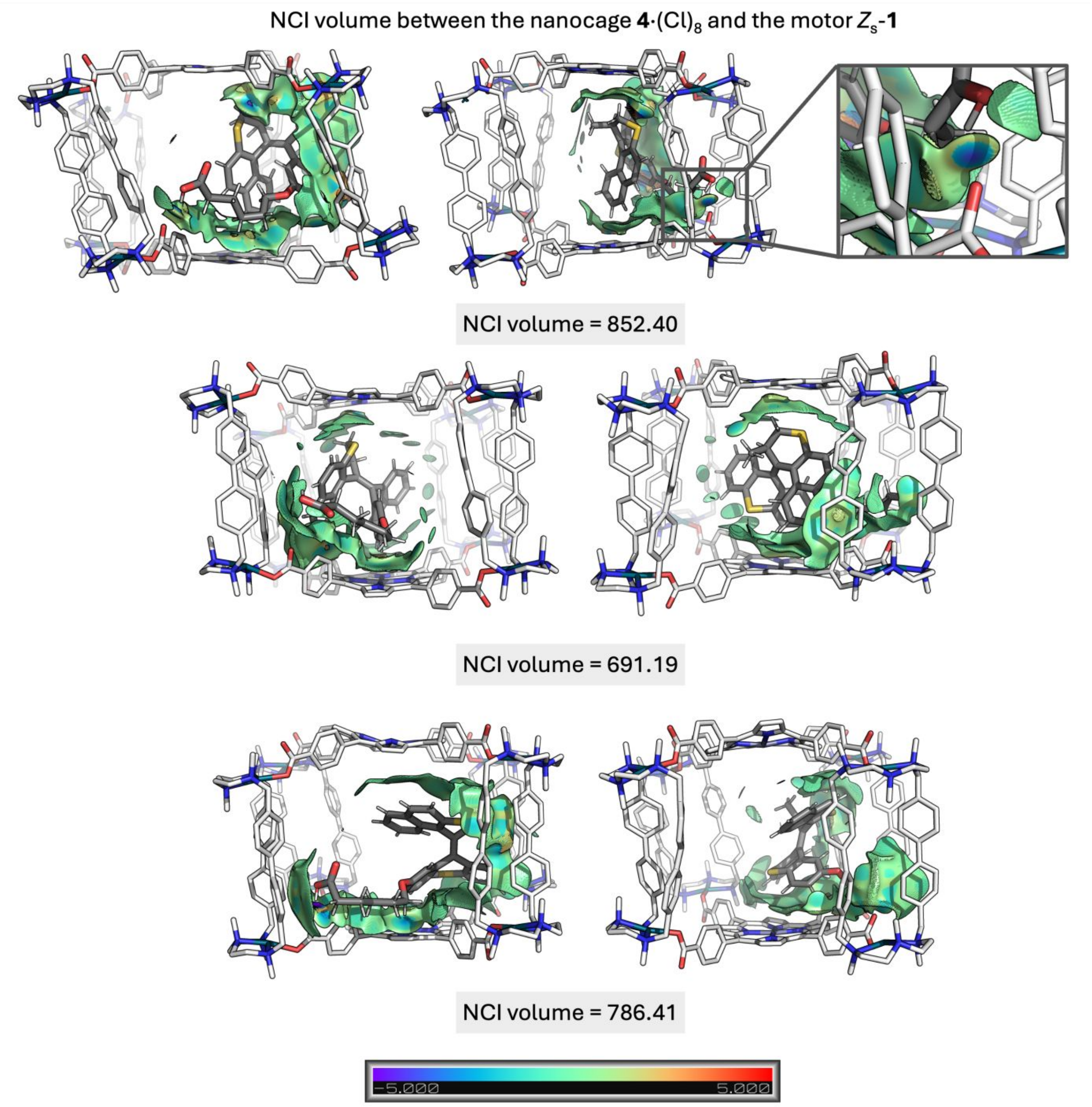

Figure S14. Visualization of the non-covalent interaction (NCI) volume calculated between the nanocage **4**·(Cl)$_8$ and the motor Z_s-**1** in three different orientations inside the cavity. A rainbow scale is used, where hydrogen bonding interaction is seen in blue (attractive interaction) and localized. Weak van der Waals interactions between the motor and the nanocage are represented in green. Value of the NCI volume in each orientation shown in Å^3.

V. *In-situ* NMR experiments in bulk solution.

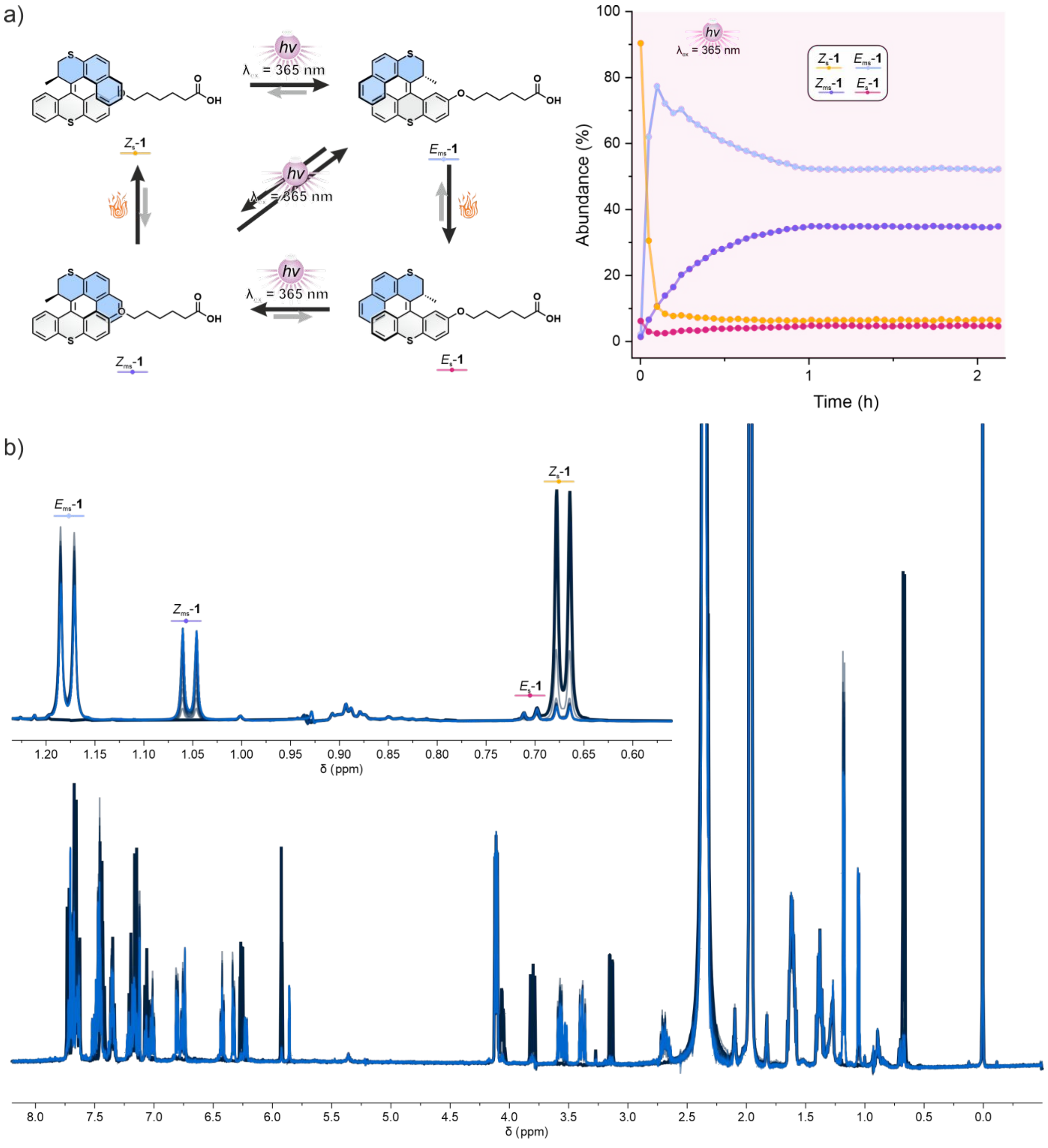

Figure S15. Kinetics of the photoisomerization of Z_s-**1** to E_{ms}-**1** (1 mM, acetonitrile-d_3) with 365 nm irradiation at -10 °C for 2 h. a) Kinetic traces of the evolution of the different isomers of **1** during the process. b) Evolution of the ^{1}H NMR spectrum (from black to blue) during *in-situ* irradiation. *Inset*: Expansion of the methyl signals of the different isomers of **1**. Note: Prolongated irradiation beyond the 5 minutes triggers the E_{ms}-**1** to Z_{ms}-**1**. To avoid this secondary process irradiation was stopped after 5 minutes (*vide infra*) in each photochemical step.

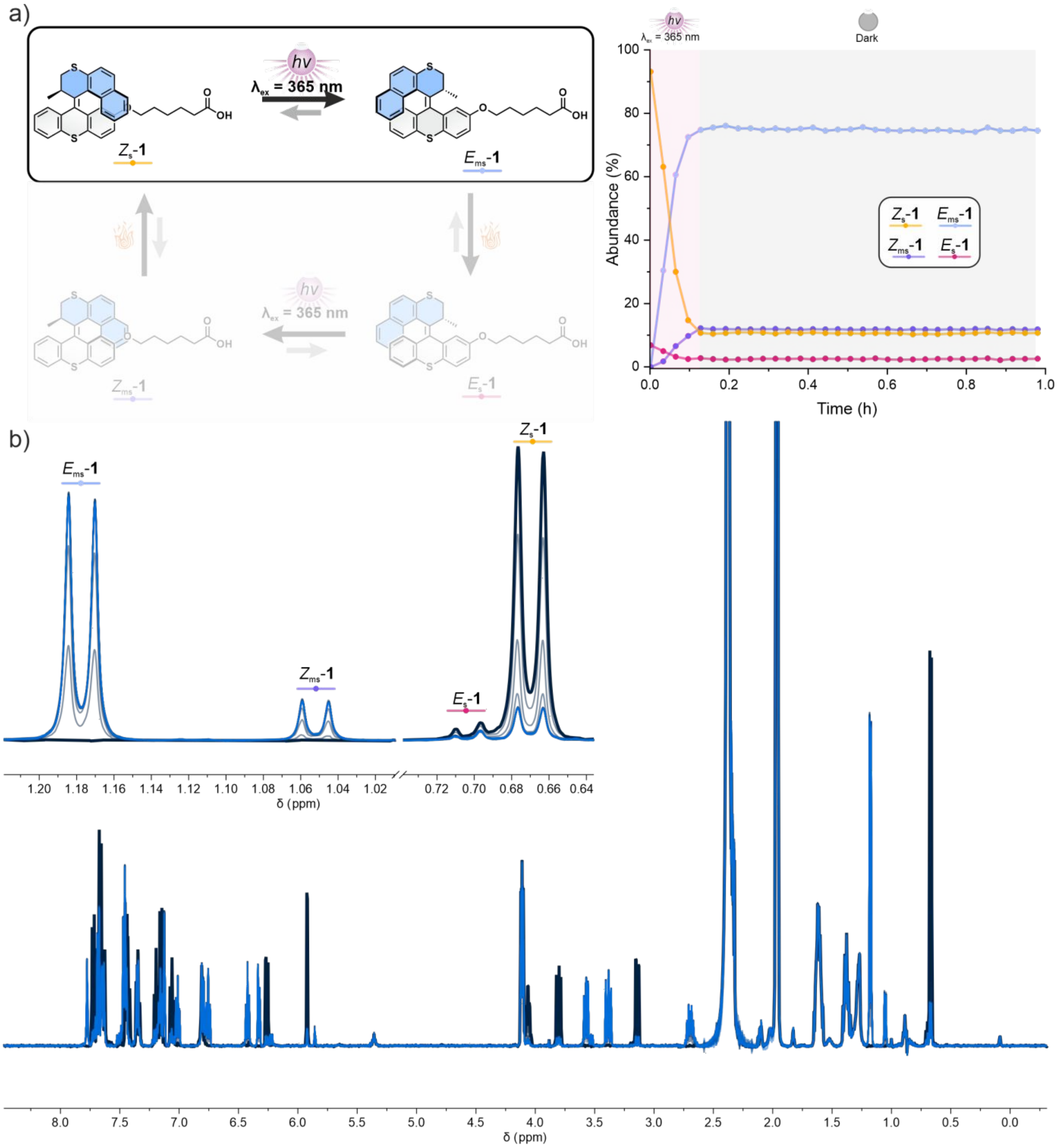

Figure S16. Kinetics of the photoisomerization of Z_s-**1** to E_{ms}-**1** (1 mM, acetonitrile-d_3) with 365 nm at -10 °C for 5 min followed by a dark period. a) Kinetic traces of the evolution of the different isomers of **1** during the process. b) Evolution of the ^{1}H NMR spectrum (from black to blue) during *in-situ* irradiation. *Inset*: Expansion of the methyl signals of the different isomers of **1**.

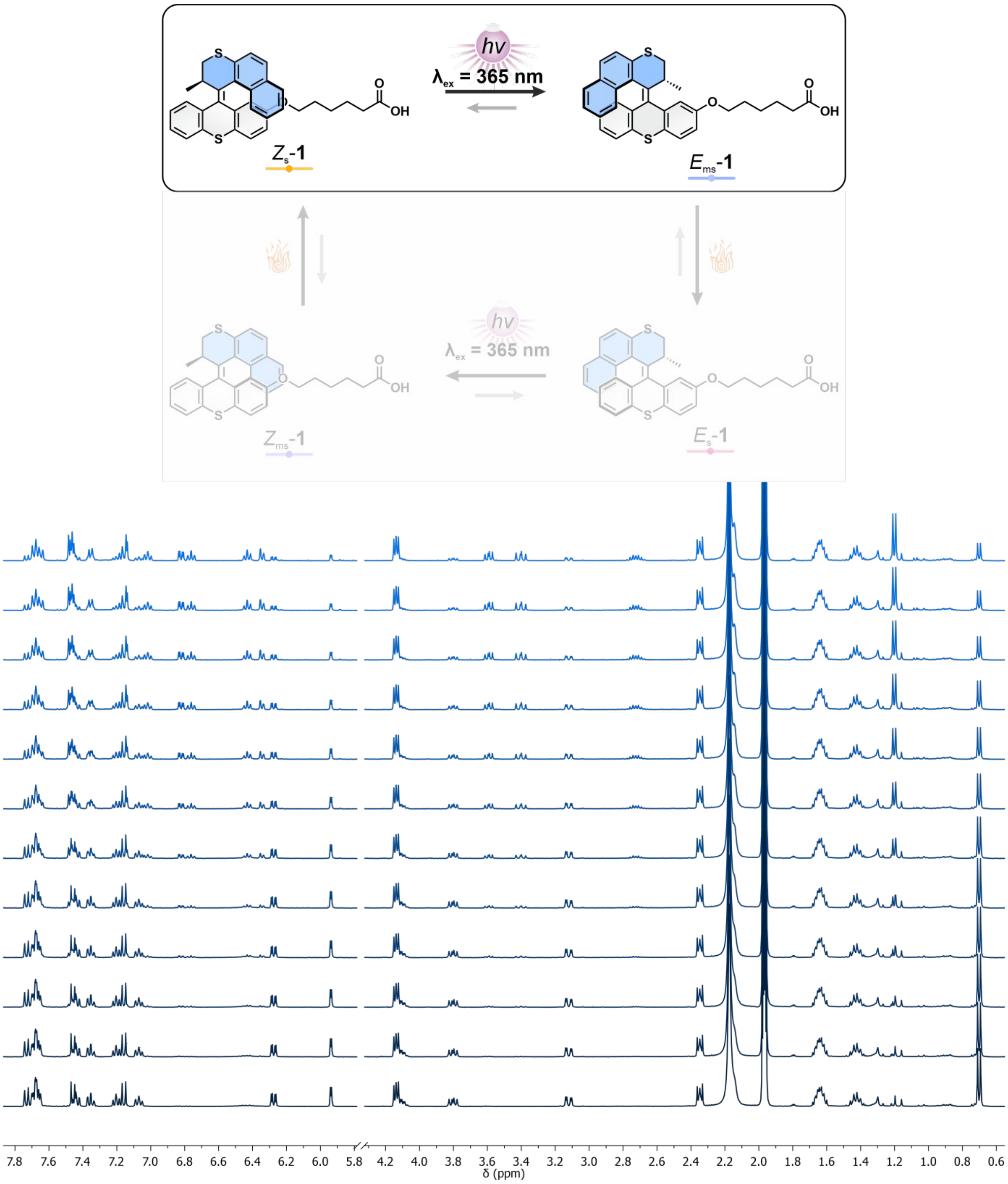

Figure S17. Kinetics of the photoisomerization of Z_s-**1** to E_{ms}-**1** (1 mM, acetonitrile-d_3) with 365 nm with a lower irradiation intensity compared to Figure S16 for a better time resolution.

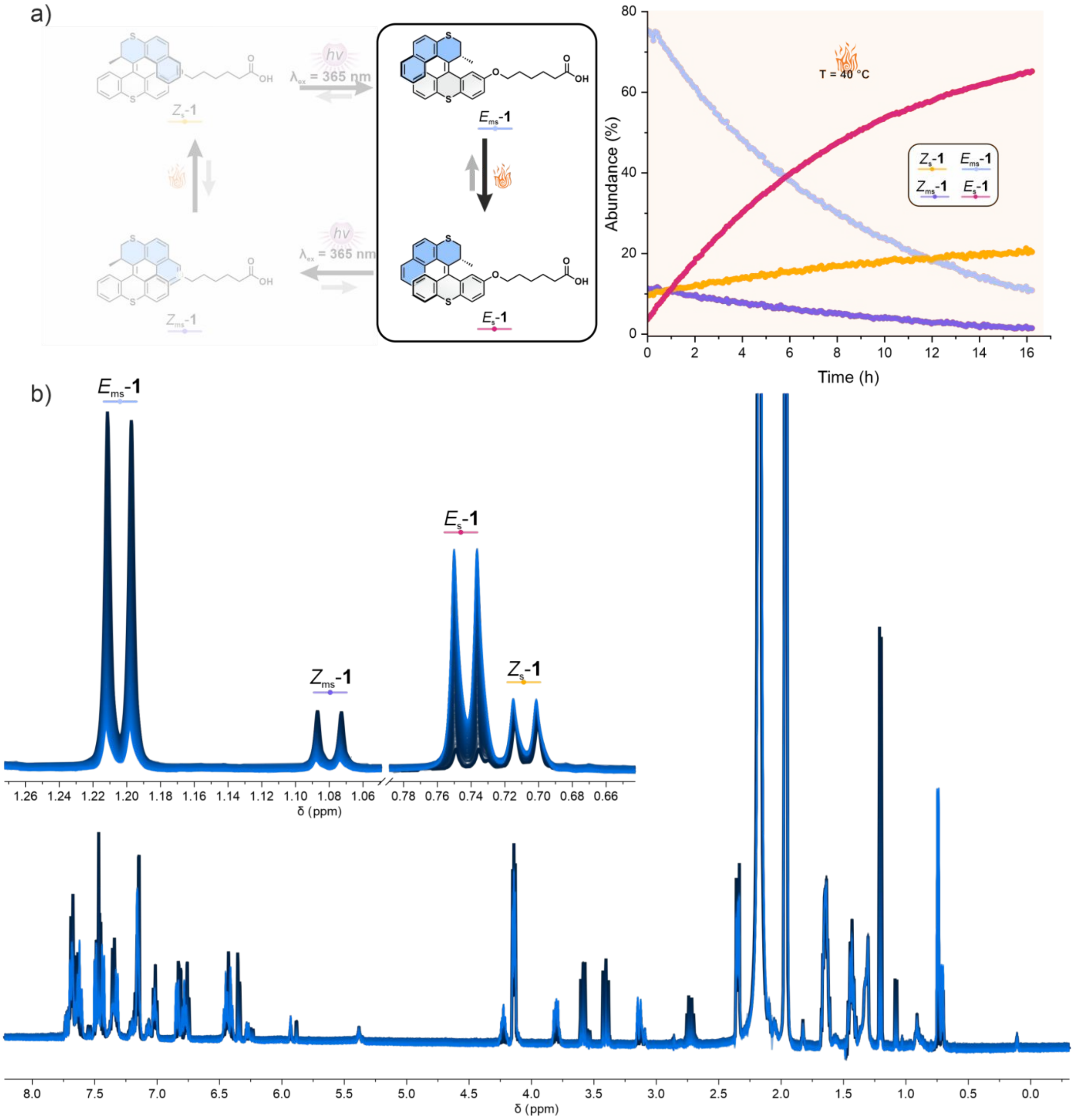

Figure S18. Kinetics of the thermal relaxation of E_{ms}-**1** to E_s-**1** (1 mM, acetonitrile-d_3) at 40 °C. a) Kinetic traces of the evolution of the different isomers of **1** during the process. b) Evolution of the ^{1}H NMR spectrum (from black to blue) during *in-situ* thermal relaxation. *Inset*: Expansion of the methyl signals of the different isomers of **1**.

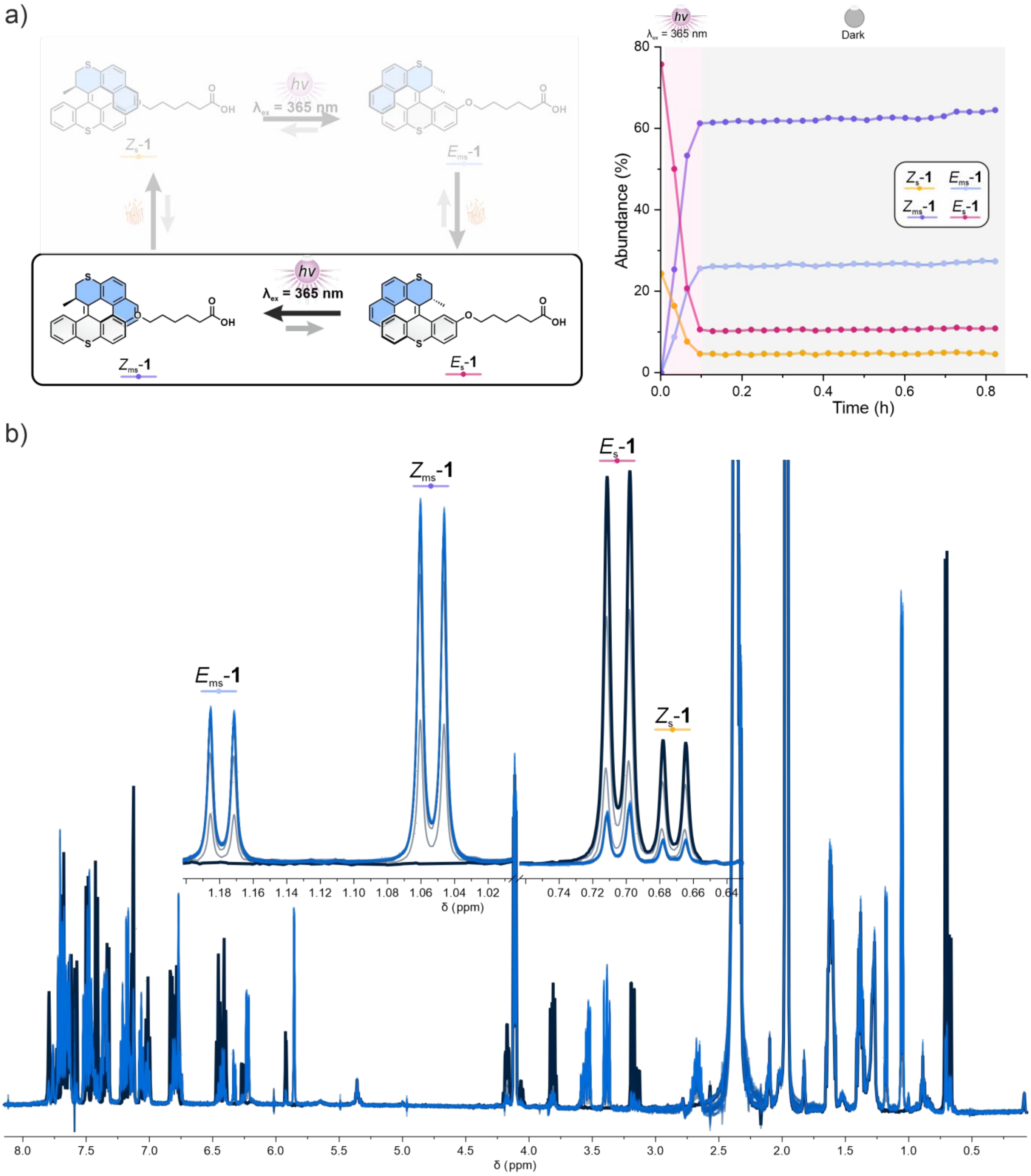

Figure S19. Kinetics of the photoisomerization of E_s-**1** to Z_{ms}-**1** (1 mM, acetonitrile-d_3) with 365 nm at -10 °C. a) Kinetic traces of the evolution of the different isomers of **1** during the process. b) Evolution of the ^{1}H NMR spectrum (from black to blue) during *in-situ* irradiation. *Inset*: Expansion of the methyl signals of the different isomers of **1**.

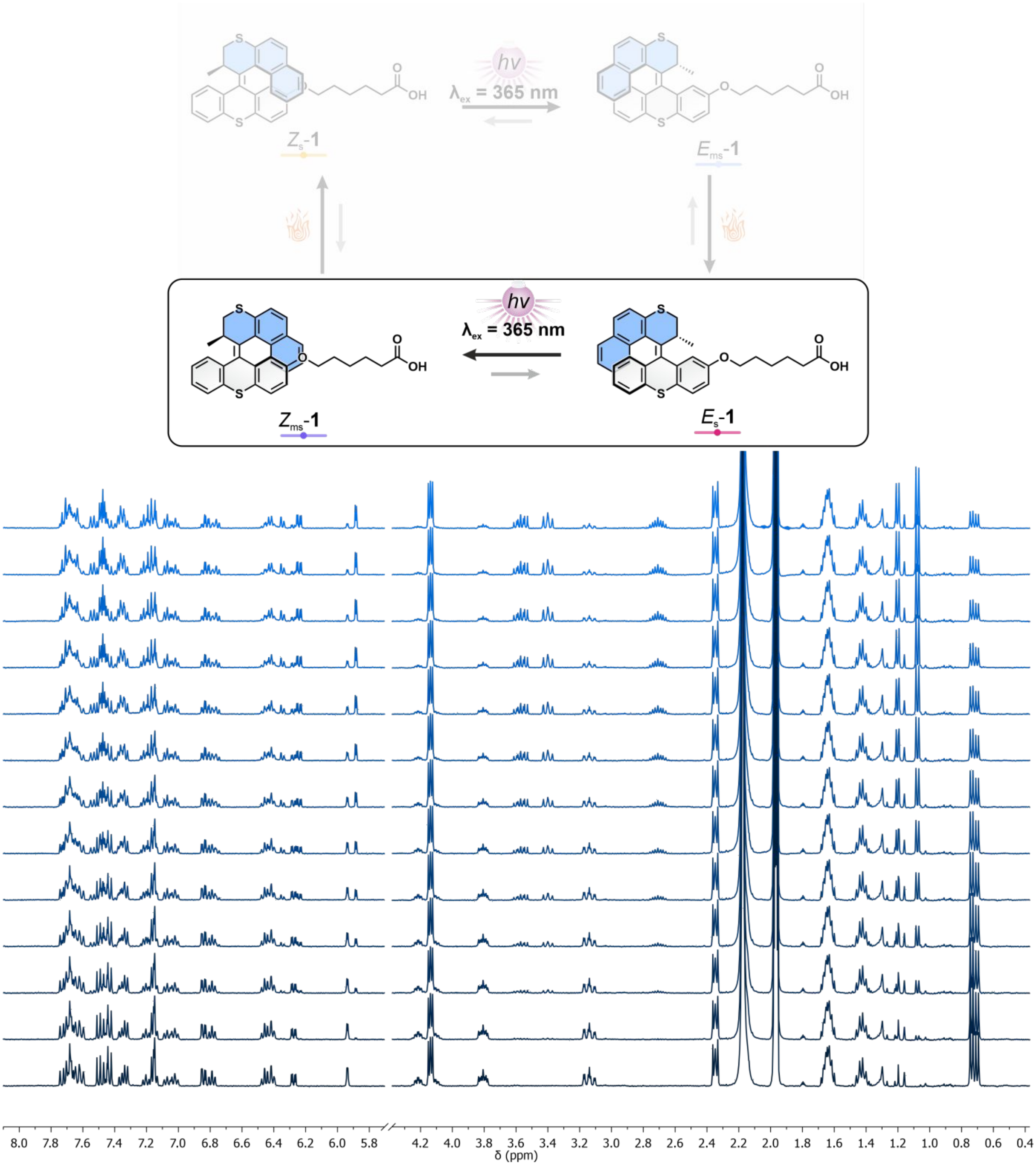

Figure S20. Kinetics of the photoisomerization of E_s-**1** to Z_{ms}-**1** (1 mM, acetonitrile-d_3) with 365 nm with a lower irradiation intensity compared to Figure S19 for improved time resolution.

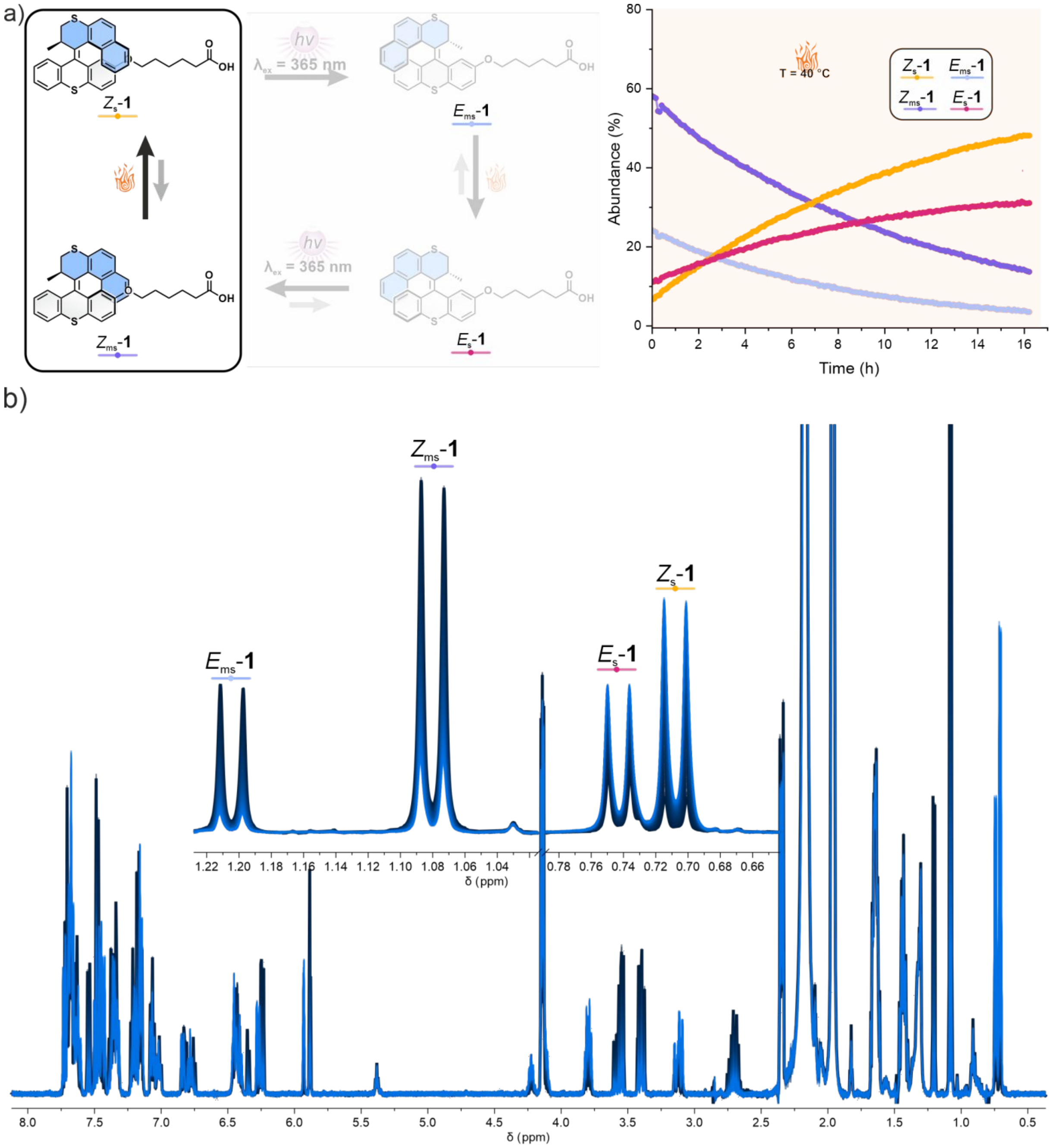

Figure S21. Kinetics of the thermal relaxation of Z_{ms}-**1** to Z_s-**1** (1 mM, acetonitrile-d_3) at 40 °C. a) Kinetic traces of the evolution of the different isomers of **1** during the process. b) Evolution of the ^1H NMR spectrum (from black to blue) during *in-situ* thermal relaxation. *Inset*: Expansion of the methyl signals of the different isomers of **1**.

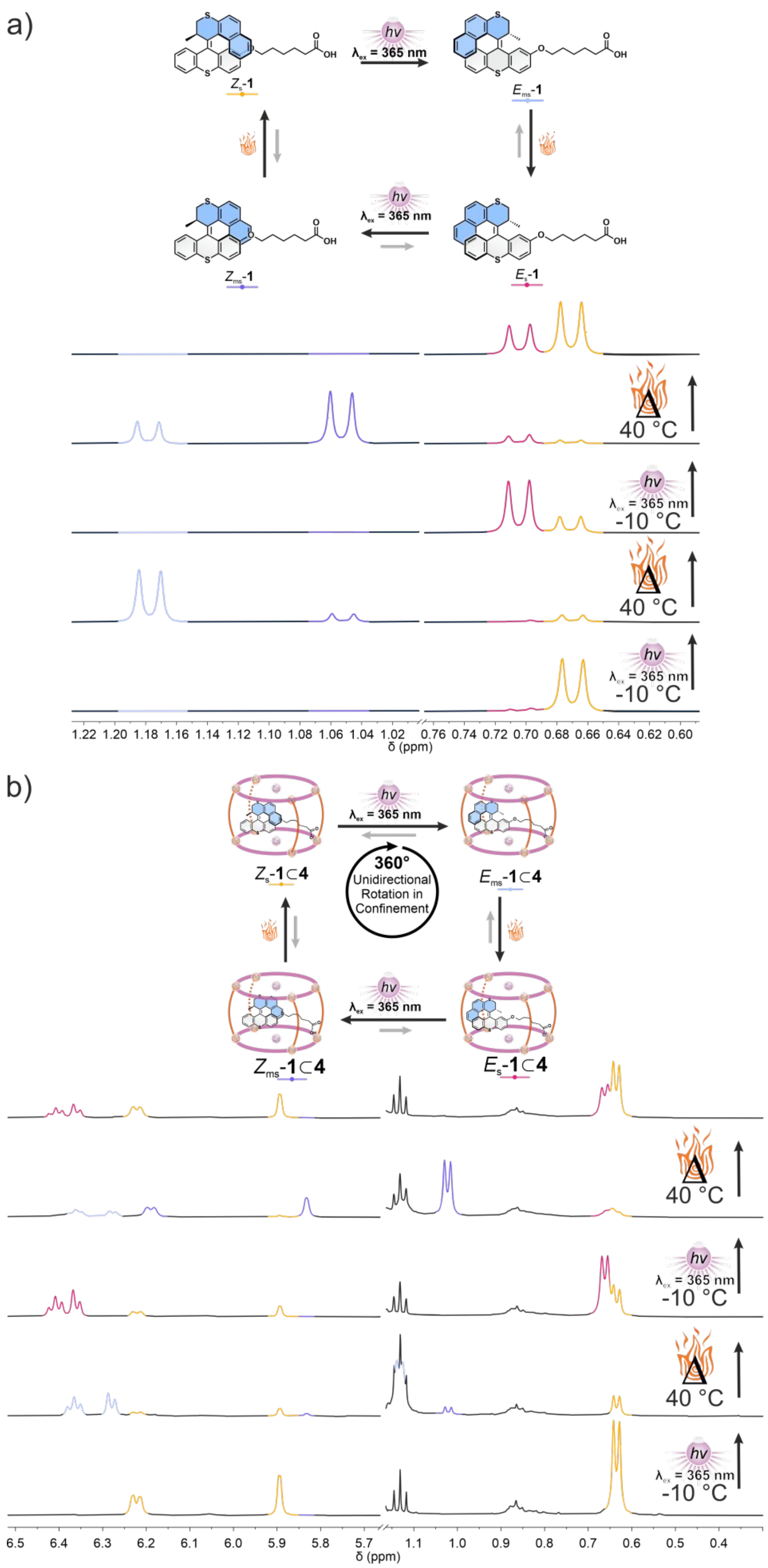

Figure S22. Stacked spectra ordered from bottom to top for the different motor **1** rotation steps. a) In bulk and b) in the confined space of cage **4**·(BArF)$_8$. *Note:* residual diethyl ether signal overlaps with the methyl peak of E_{ms}-**1⊂4**·(BArF)$_8$.

VI. CD spectroscopy of motor in bulk solution

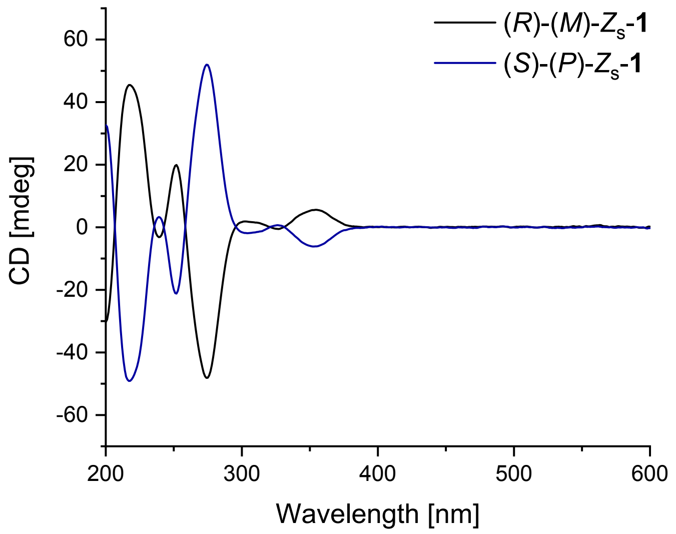

Figure S23. CD spectra (acetonitrile, □15x10^{-6} M, 20 °C) of (*R*)-(*M*)-Z_s-**1** (blue) and (*S*)-(*P*)-Z_s-**1** (black).

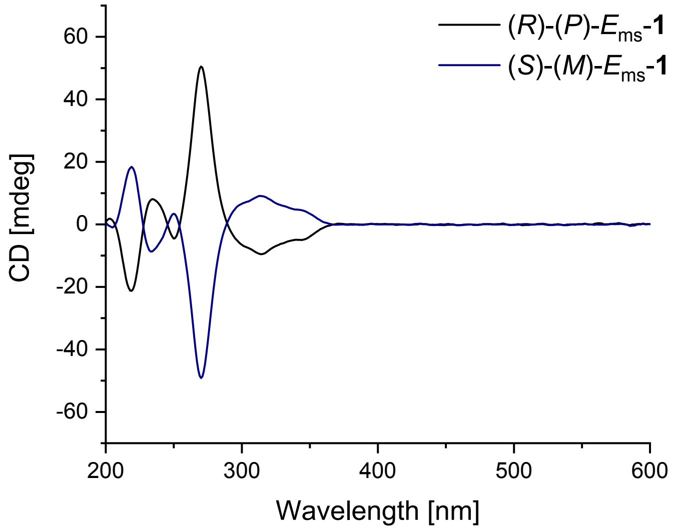

Figure S24. CD spectra (acetonitrile, □15x10^{-6} M, 20 °C) of (*R*)-(*P*)-E_{ms}-**1** (blue) and (*S*)-(*M*)-E_{ms}-**1** (black) obtained by irradiation of the corresponding stable states with 365 nm UV light.

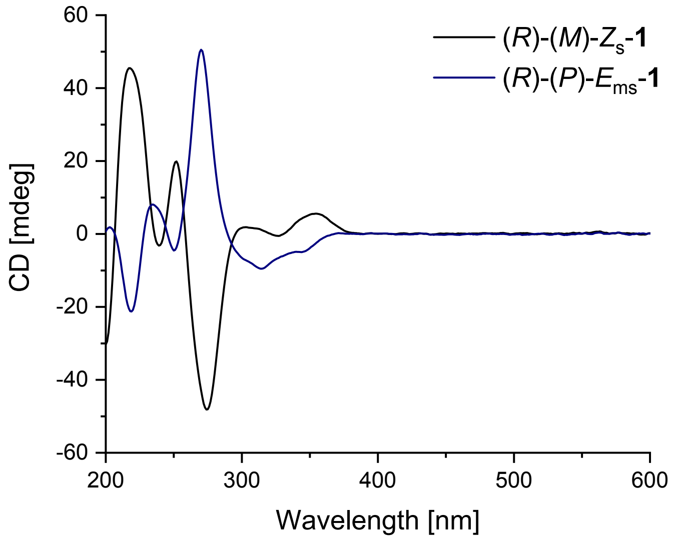

Figure S25. CD spectra (acetonitrile, □15x10^{-6} M, 20 °C) of (*R*)-(*M*)-Z_s-**1** (blue) and (*R*)-(*P*)-E_{ms}-**1** obtained by irradiation with 365 nm UV light (blue).

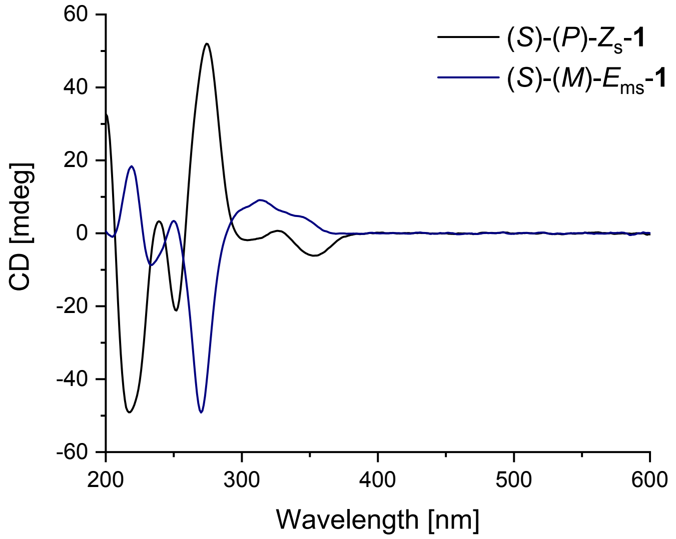

Figure S26. CD spectra (acetonitrile, □15x10^{-6} M, 20 °C) of (*S*)-(*P*)-Z_s-**1** (blue) and metastable (*R*)-(*M*)-E_{ms}-**1** obtained by irradiation with 365 nm UV light (blue).

VII. NMR experiments in confined space.

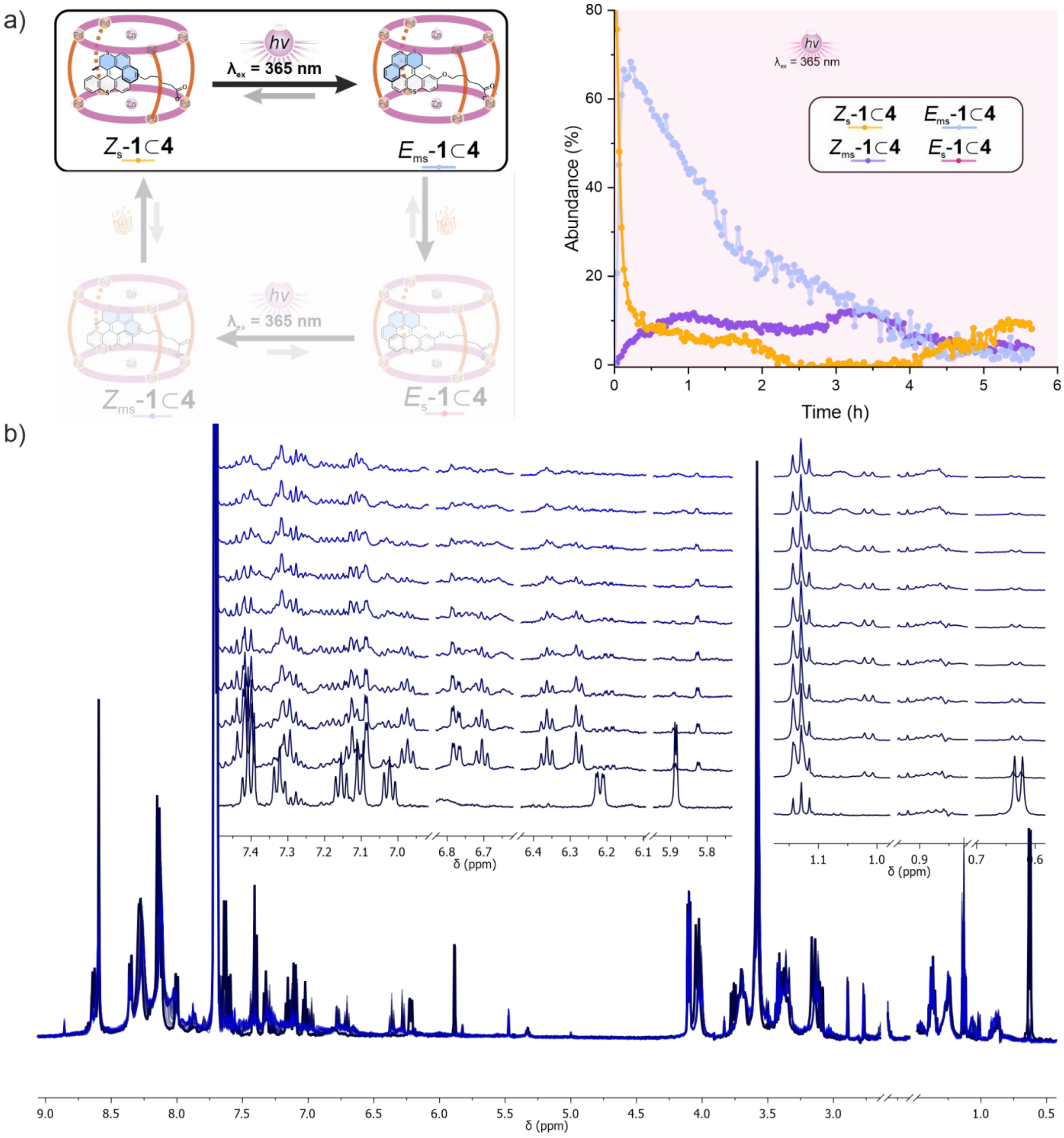

Figure S27. Kinetics of the photoisomerization of Z_s-**1** to E_{ms}-**1** (1 mM, acetonitrile-d_3) and decomposition with 365 nm at -10 °C inside the cage (1 mM, acetonitrile-d_3). a) Kinetic traces of the evolution of the different isomers of **1** during the process. b) Evolution of the ^{1}H NMR spectrum (from black to blue) during *in-situ* thermal relaxation. *Inset*: Expansion of the methyl signals of the different isomers of **1**. R = -O(CH$_2$)$_5$COOH and characteristic aromatic motor signals.

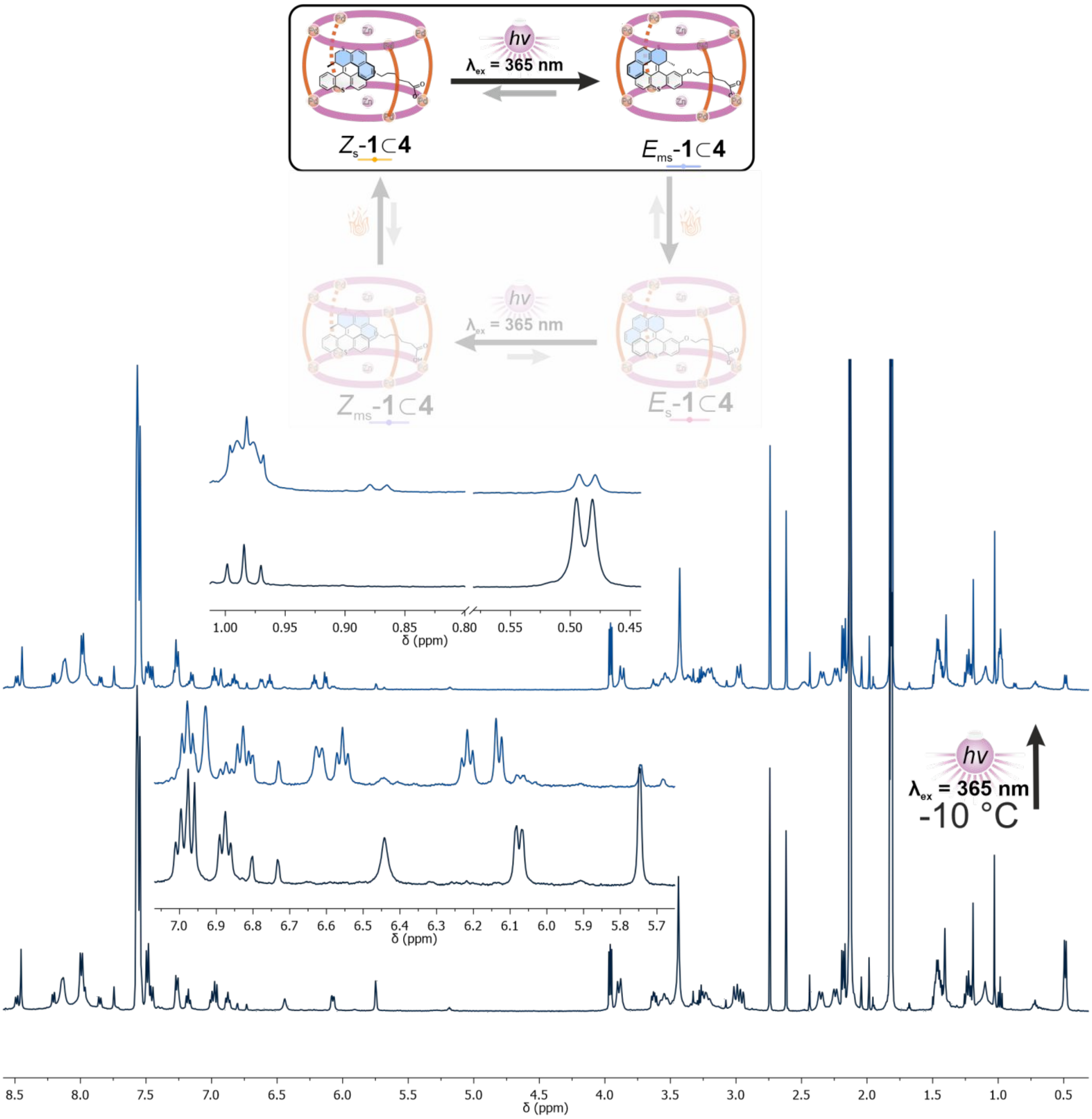

Figure S28. Stacked spectra from bottom to top for the photoisomerization of Z_s-**1⊂4**·(BArF)$_8$ to E_{ms}-**1⊂4**·(BArF)$_8$ (1 mM, acetonitrile-d$_3$) with 365 nm *ex-situ* irradiation in a degassed J Young NMR tube. *Inset*: Expansion of the methyl and aromatic signals. *Note:* residual diethyl ether signal overlaps with the methyl peak of E_{ms}-**1⊂4**·(BArF)$_8$. R = -O(CH$_2$)$_5$COOH.

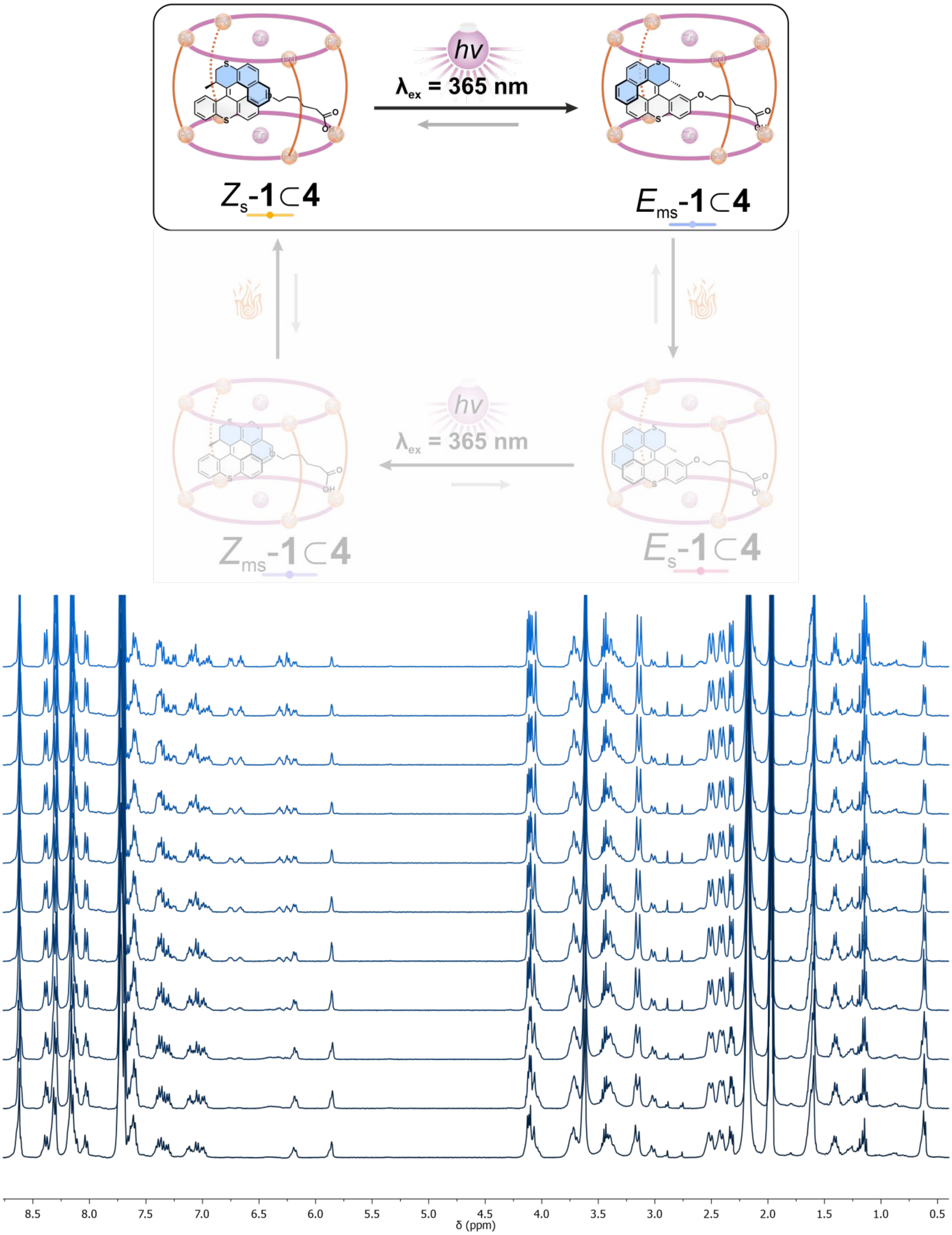

Figure S29. Kinetics of the photoisomerization of Z_s-**1⊂4**·(BArF)$_8$ to E_{ms}-**1⊂4**·(BArF)$_8$ (1 mM, acetonitrile-d$_3$) with 365 nm with a lower irradiation intensity compared to Figure S28 for improved time resolution.

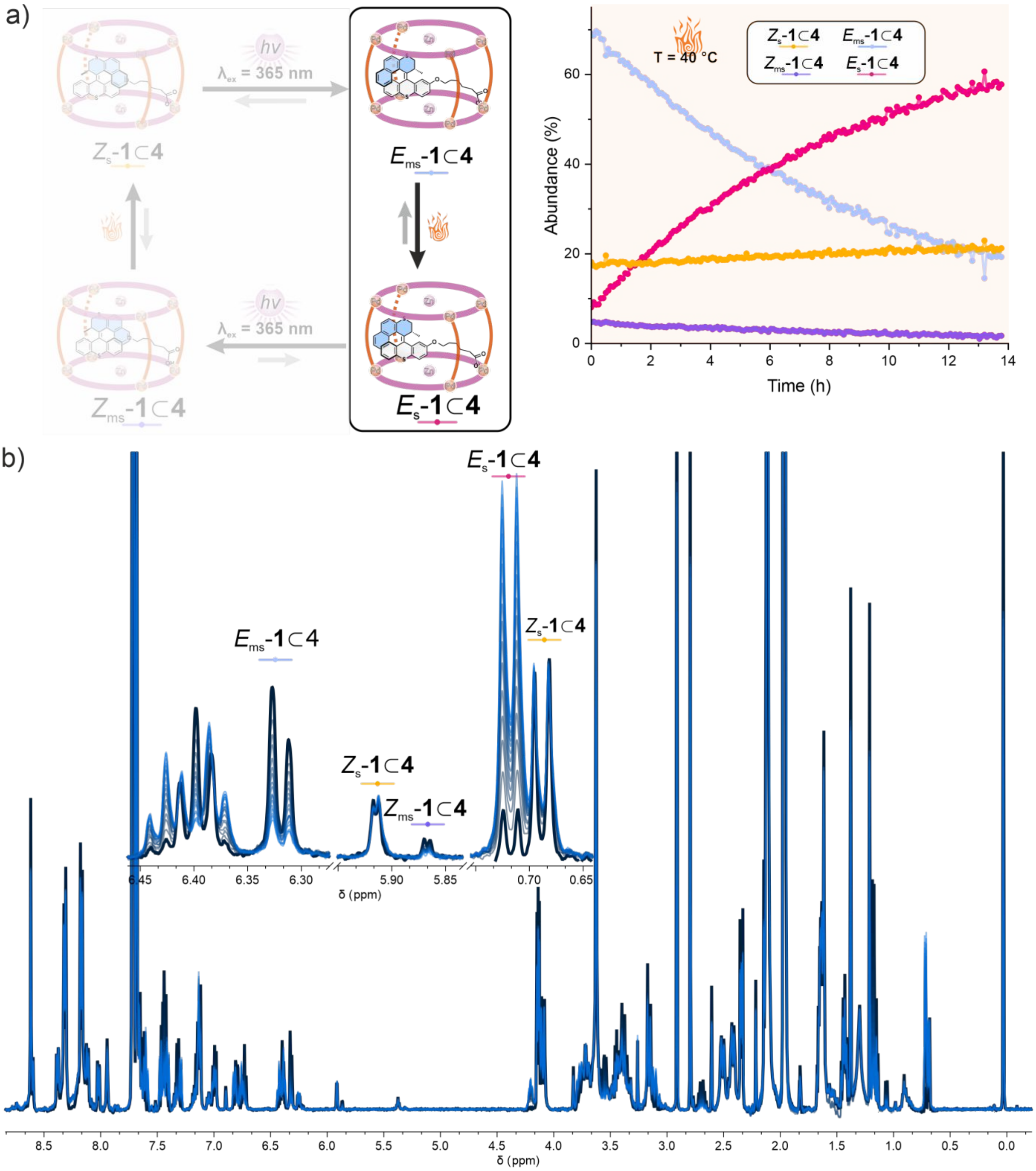

Figure S30. Kinetics of the thermal relaxation of E_{ms}-**1** to E_{s}-**1** inside the cage (1 mM, acetonitrile-d_3) at 40 °C after *ex-situ* irradiation (degassed, 5 miuntes, λ_{irr} = 365 nm). a) Kinetic traces of the evolution of the different isomers of **1** during the process. b) Evolution of the ^{1}H NMR spectrum (from black to blue) during *in-situ* thermal relaxation. *Inset*: Expansion of the methyl signals of the different isomers of **1** and characteristic aromatic motor signals. R = -O(CH$_2$)$_5$COOH.

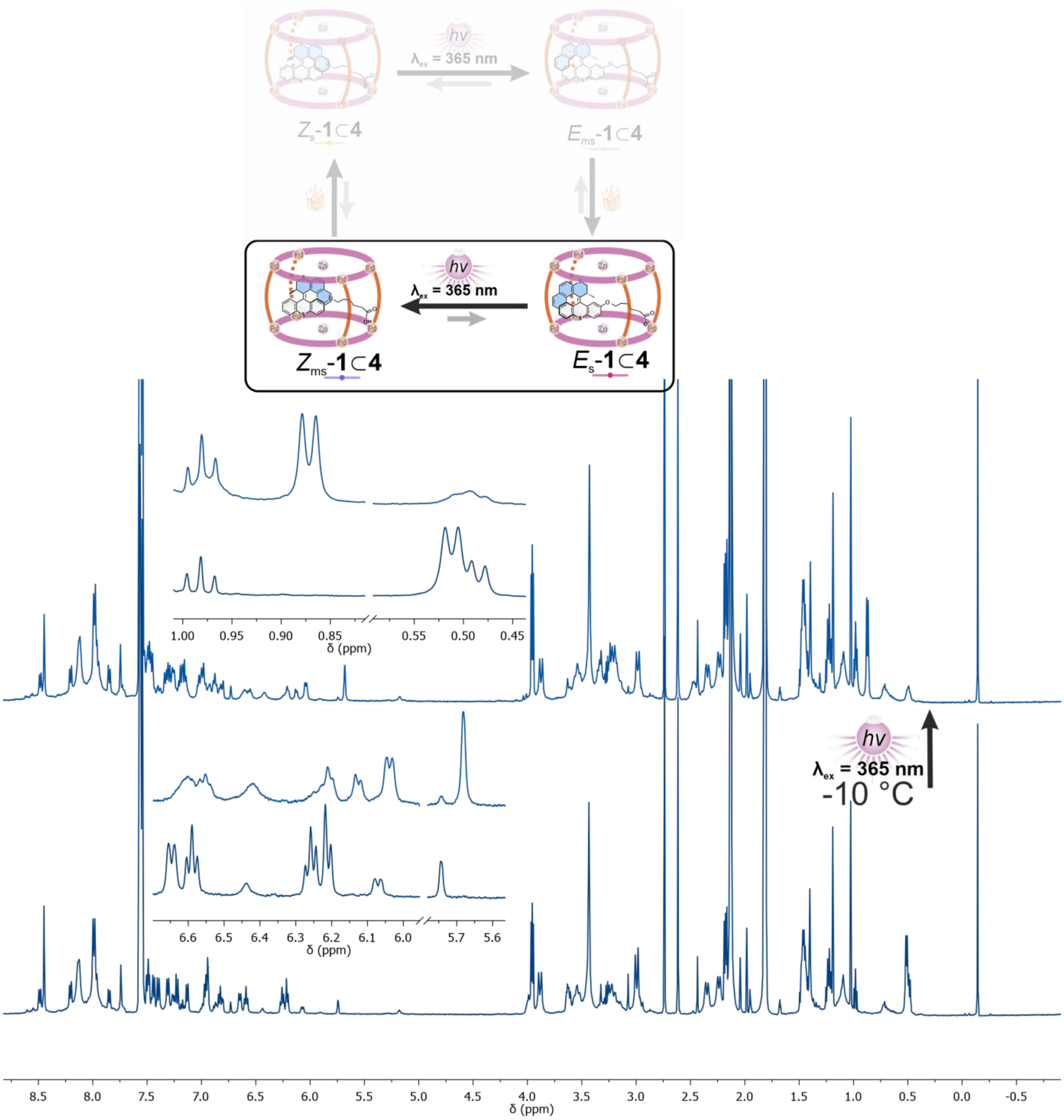

Figure S31. Stacked spectra from bottom to top for the photoisomerization of E_s-**1⊂4**·(BArF)$_8$ to Z_{ms}-**1⊂4**·(BArF)$_8$ (1 mM, acetonitrile-d$_3$) with 365 nm *ex situ* irradiation in a degassed J Young NMR tube. *Inset*: Expansion of the methyl and aromatic signals. R = -O(CH$_2$)$_5$COOH.

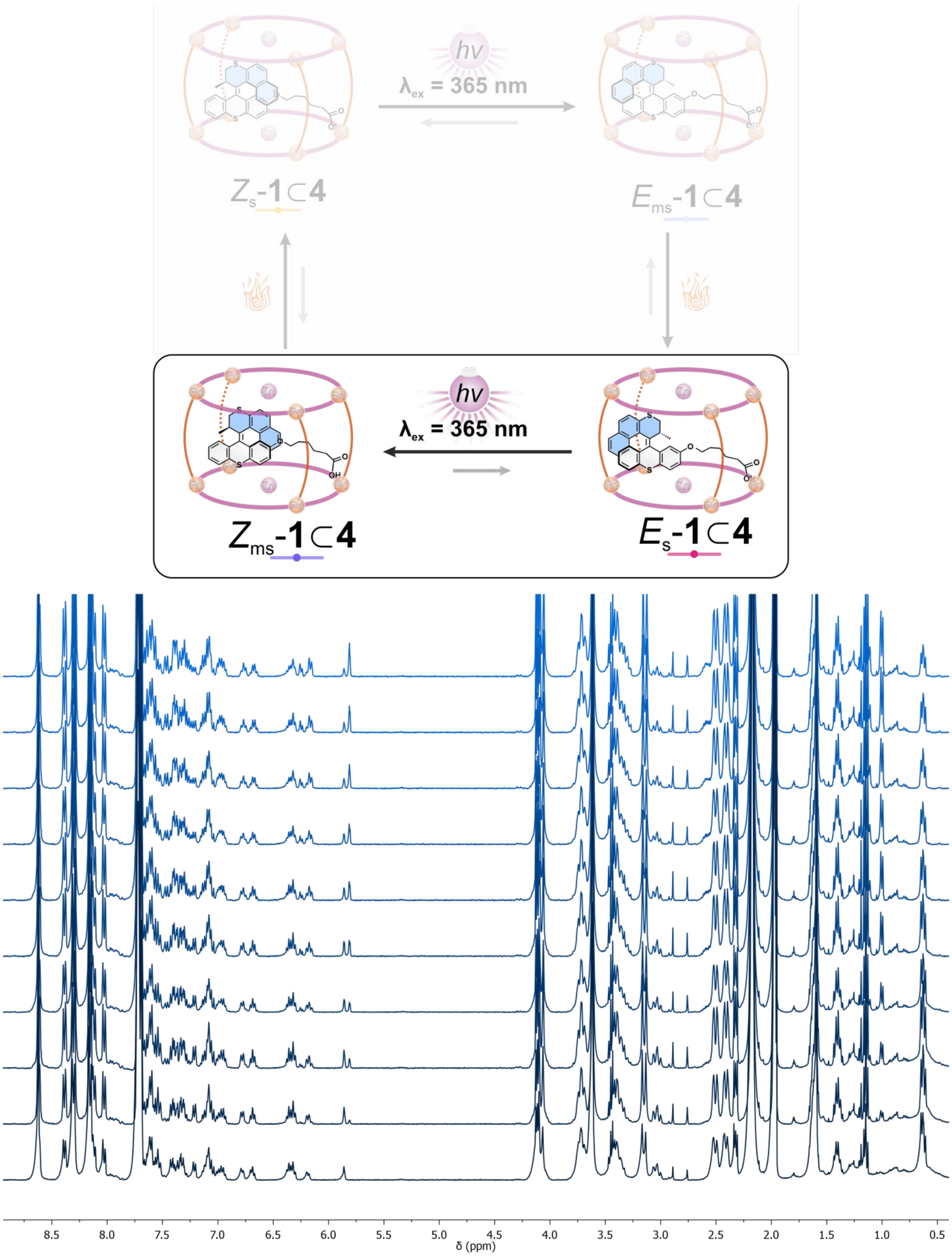

Figure S32. Kinetics of the photoisomerization of E_s-**1⊂4**·(BArF)$_8$ to Z_{ms}-**1⊂4**·(BArF)$_8$ (1 mM, acetonitrile-d$_3$) with 365 nm with a lower irradiation intensity compared to Figure S31 for improved time resolution.

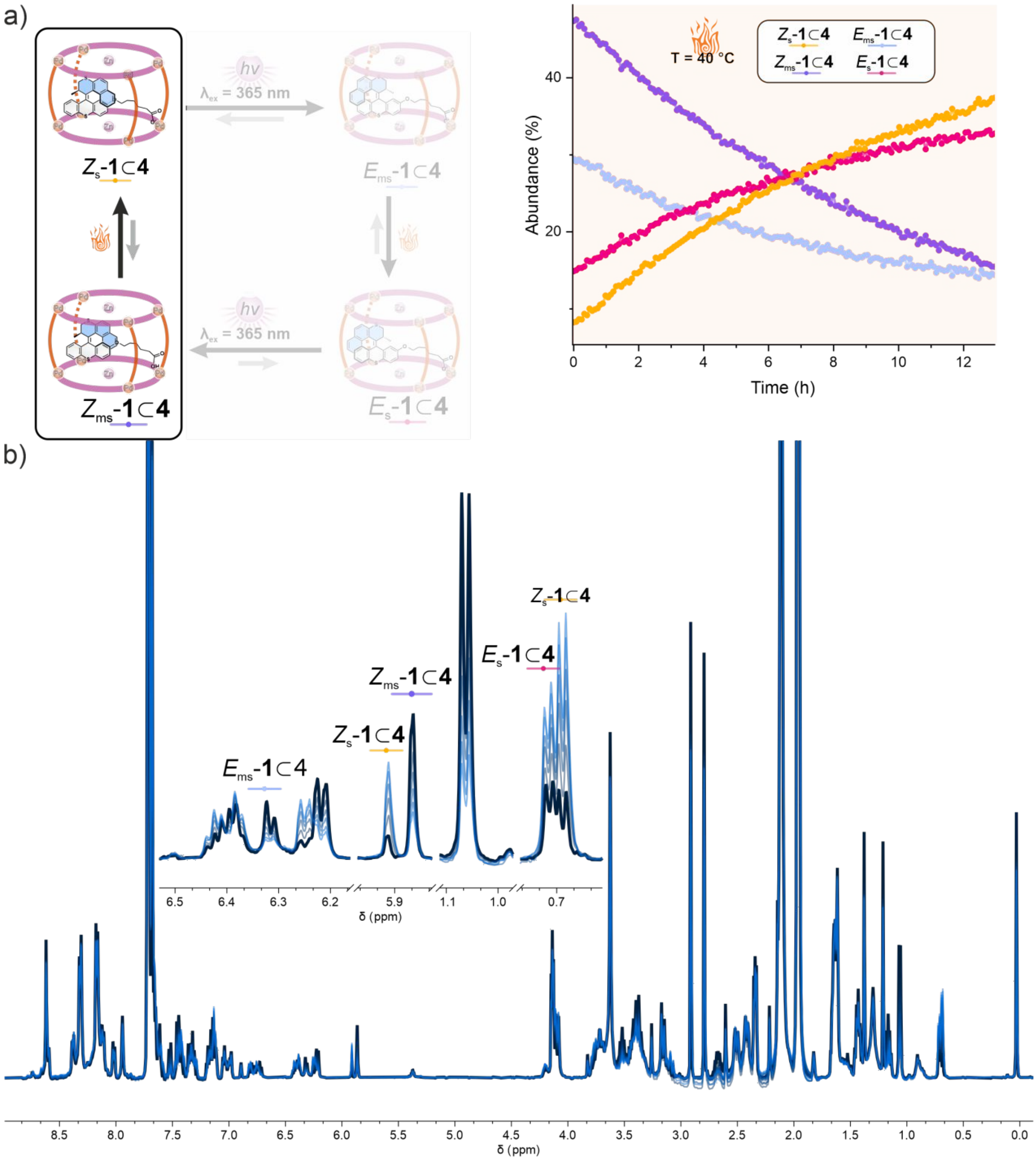

Figure S33. Kinetics of the thermal relaxation of Z_{ms}-**1** to Z_s-**1** inside the cage (1 mM, acetonitrile-d$_3$) at 40 °C. a) Kinetic traces of the evolution of the different isomers of **1** during the process. b) Evolution of the ^{1}H NMR spectrum (from black to blue) during *in-situ* thermal relaxation. *Inset*: Expansion of the methyl signals of the different isomers of **1**. R = -O(CH$_2$)$_5$COOH.

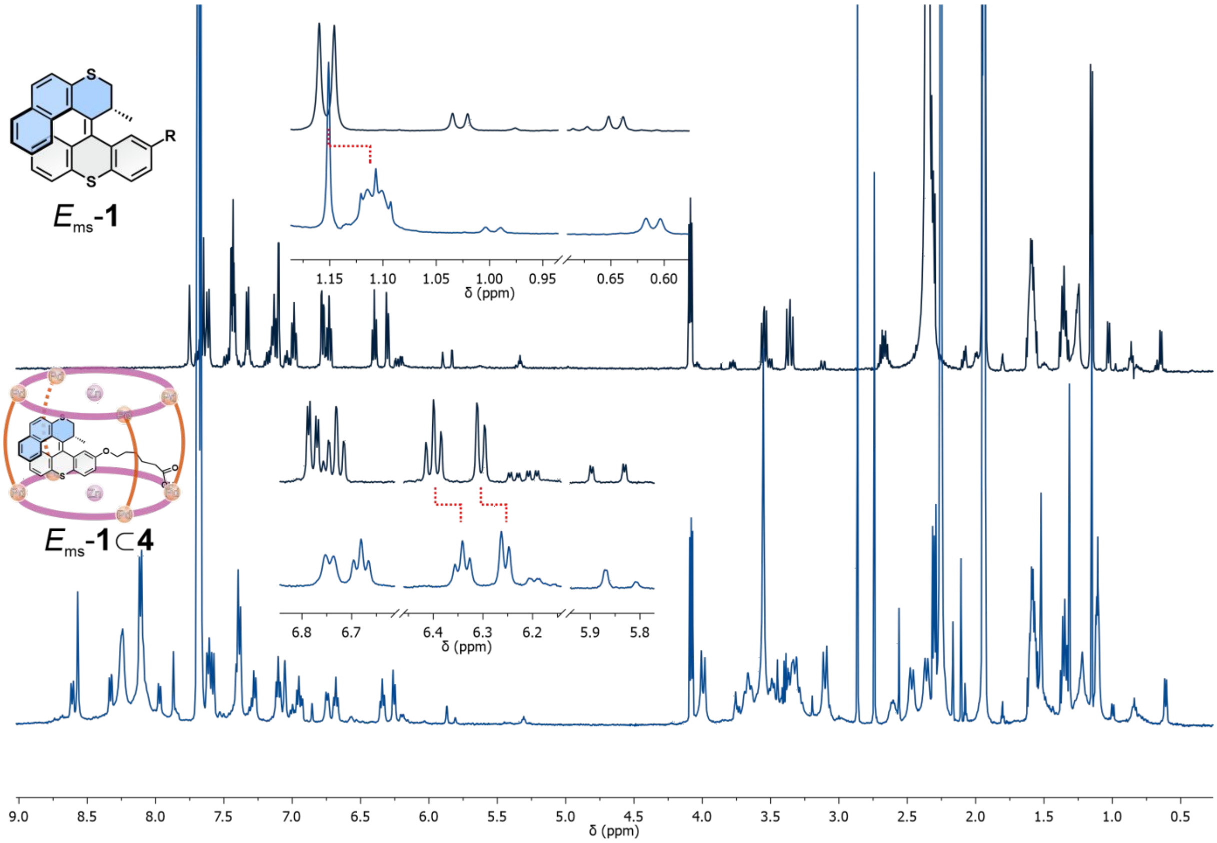

Figure S34. Comparison of the ^{1}H NMR signals of the motor isomer *E*$_{ms}$-**1** (1 mM, acetonitrile-d_3) in bulk (top) and in the confined space (bottom). *Note:* residual diethyl ether signal overlaps with the methyl peak of *E*$_{ms}$-**1⊂4**·(BArF)$_8$. R = -O(CH$_2$)$_5$COOH.

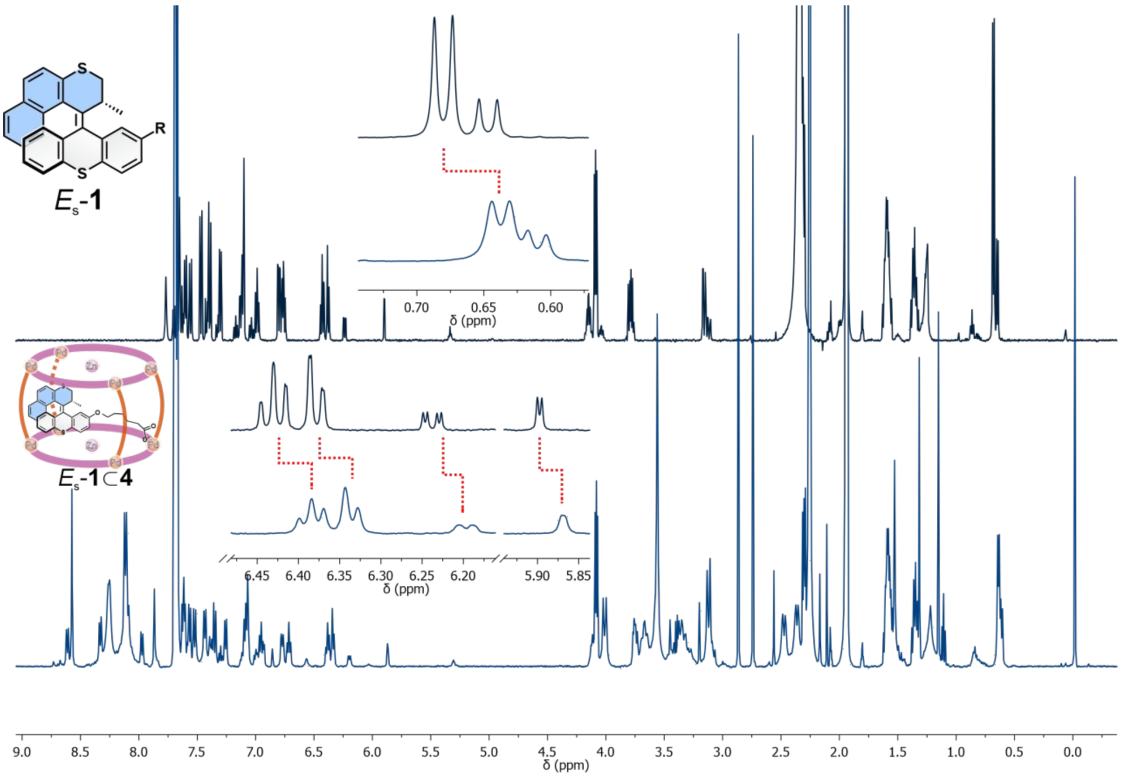

Figure S35. Comparison of the ^{1}H NMR signals of the motor isomer E_s-**1** (1 mM, acetonitrile-d_3) in bulk (top) and in the confined space (bottom). *Note:* residual diethyl ether signal overlaps with the methyl peak of E_{ms}-**1⊂4**·(BArF)$_8$. R = -O(CH$_2$)$_5$COOH.

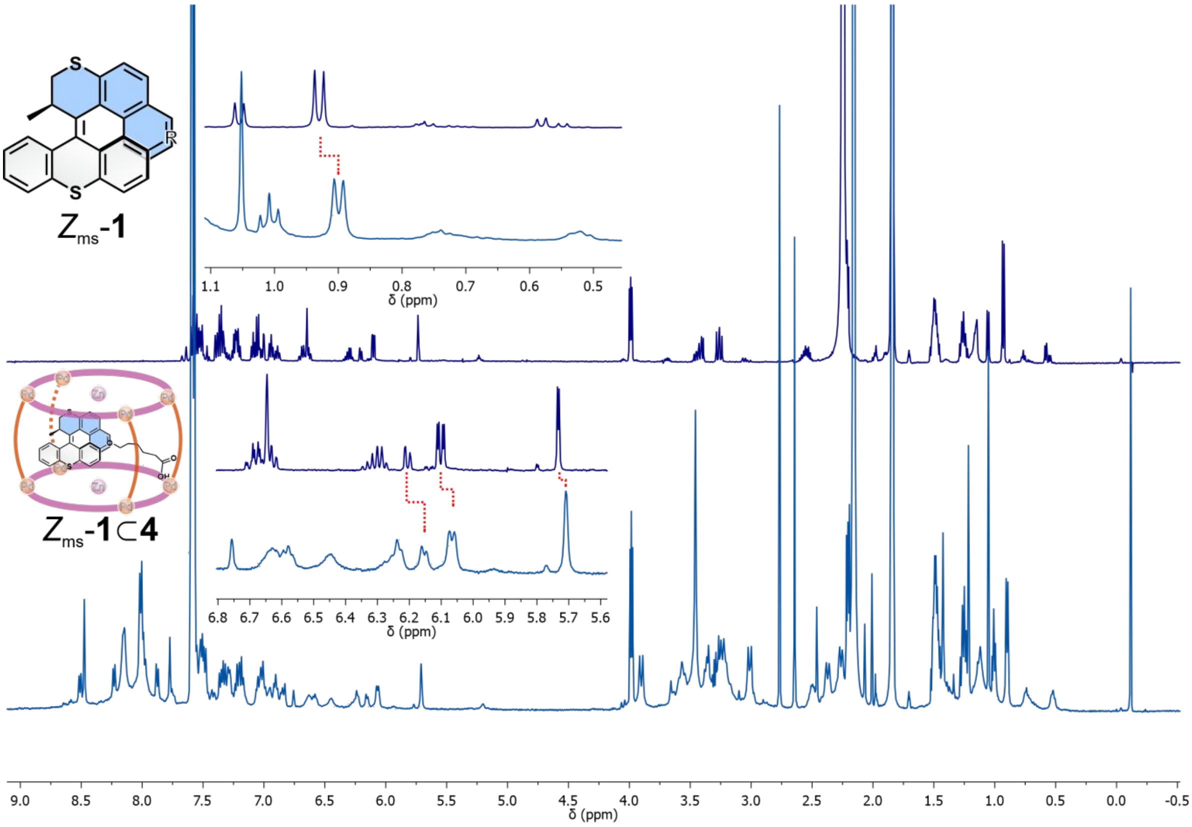

Figure S36. Comparison of the ^{1}H NMR signals of the motor isomer Z_{ms}-**1** (1 mM, acetonitrile-d_3) in bulk (top) and in the confined space (bottom). *Note:* residual diethyl ether signal overlaps with the methyl peak of E_{ms}-**1⊂4**·(BArF)$_8$. R = -O(CH$_2$)$_5$COOH.

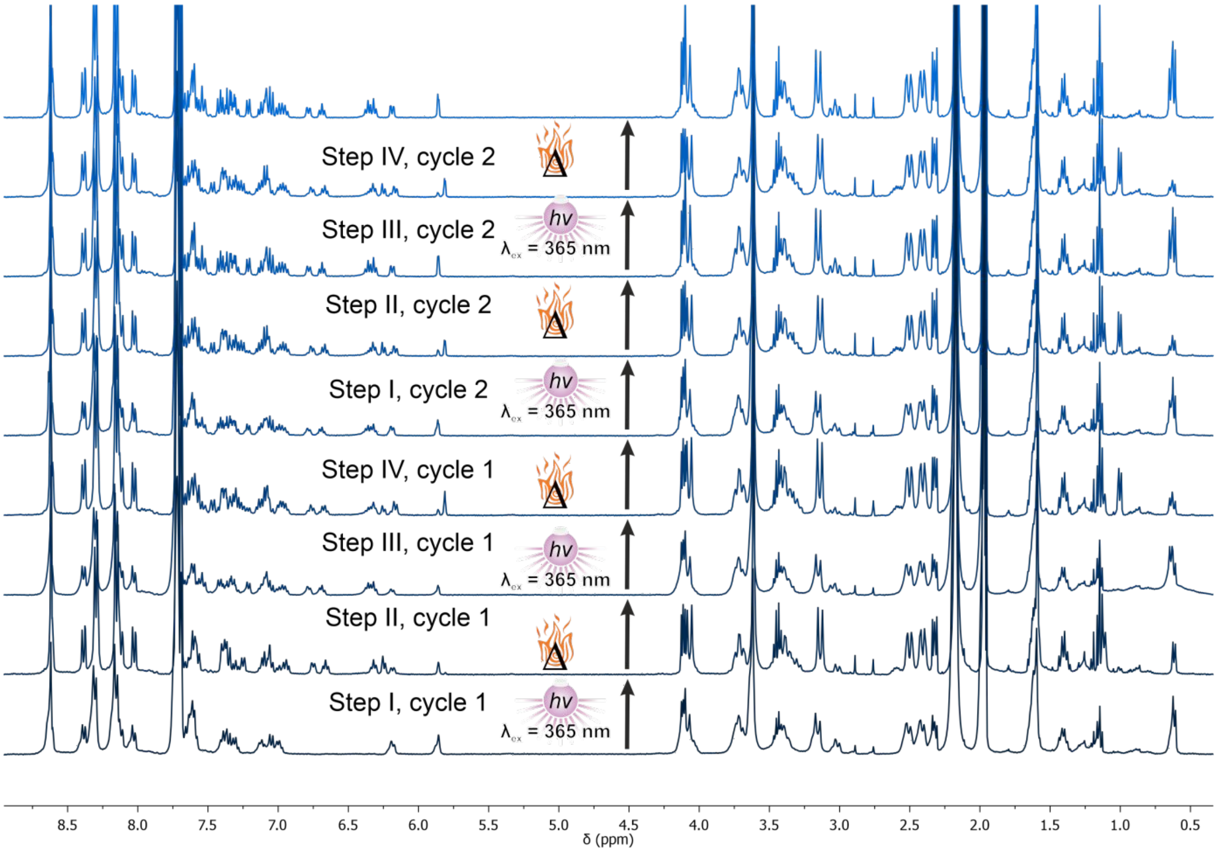

Figure S37. Two consecutive rotation cycles of **1⊂4·**(BArF)$_8$.

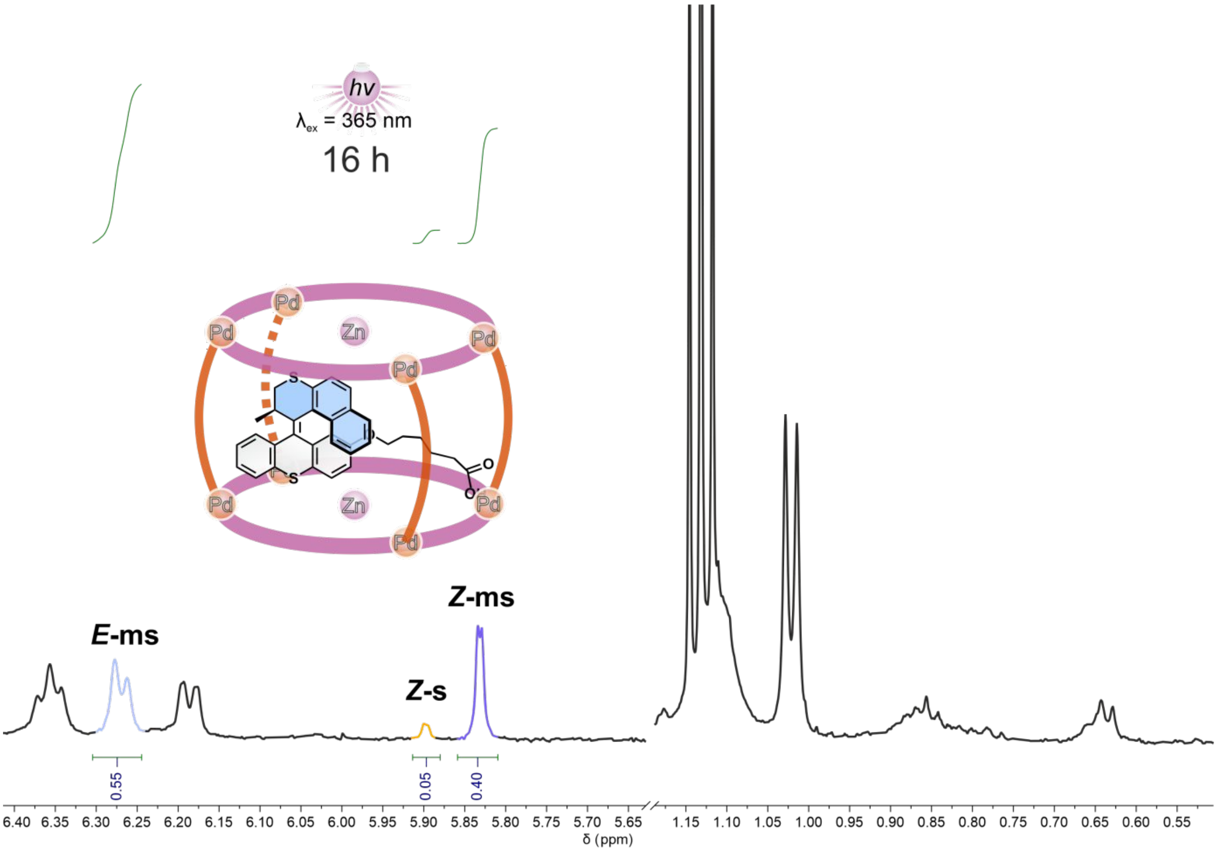

Figure S38. 1⊂4·(BArF)$_8$ 16 h irradiation at room temperature.

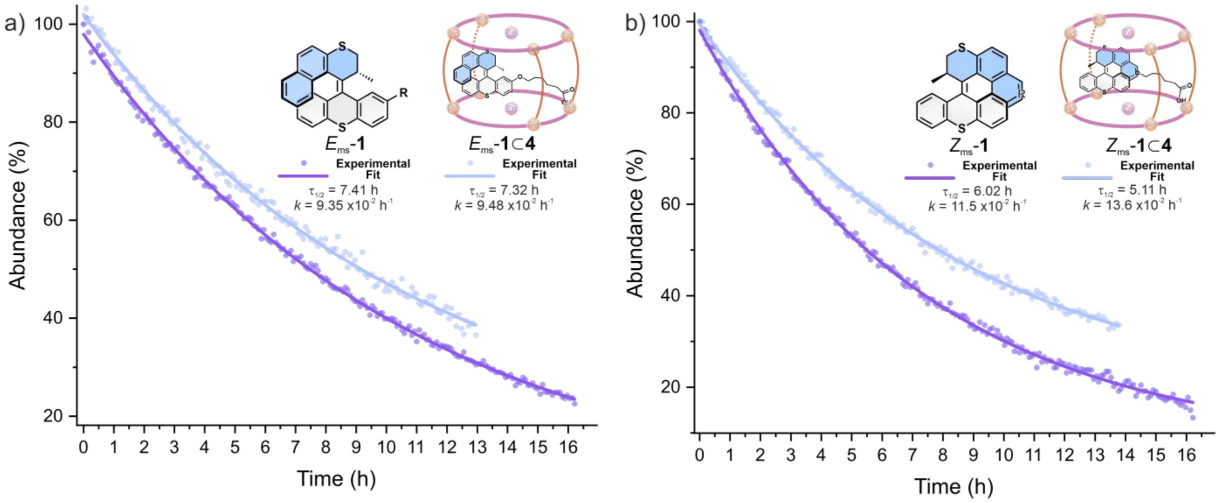

Figure S39. Thermal decay (40 °C), THI rate constant and half-life of the metastable isomers a) Z_{ms}-**1** in bulk and in the confined space of **4·**(BArF)$_8$ and b) E_{ms}-**1** in bulk and in the confined space of **4·**(BArF)$_8$.

Table S1. Isomeric distribution of **1** during the different steps of the motor rotation cycle in bulk and in the confined space of **4**·(BArF)$_8$ determined by ^{H}H NMR.

Bulk motor distribution (%)			
Z_s-**1**	E_{ms}-**1**	Z_{ms}-**1**	E_s-**1**
Pristine			
**~97%**	**N.D.**	**N.D.**	**~3**
Step I. Z_s-**1** to E_{ms}-**1** Photoisomerization			
**11**	**75**	**12**	**3**
Step II. E_{ms}-**1** to E_s-**1** THI			
**24**	**N.D.**	**N.D.**	**76**
Step III. E_s-**1** to Z_{ms} Photoisomerization			
**4**	**26**	**60**	**10**
Step III. **1** Z_{ms} to Z_s THI			
**63**	**N.D.**	**N.D.**	**37**

Confined motor distribution (%)			
Z_s-**1**	E_{ms}-**1**	Z_{ms}-**1**	E_s-**1**
Pristine			
**~97%**	**N.D.**	**N.D.**	**~3**
Step I. Z_s-**1** to E_{ms}-**1** Photoisomerization			
**18**	**75**	**7**	**N.D.**
Step II. E_{ms}-**1** to E_s-**1** THI			
**25**	**N.D.**	**N.D.**	**75**
Step III. E_s-**1** to Z_{ms} Photoisomerization			
**6**	**29**	**58**	**7**
Step III. **1** Z_{ms} to Z_s THI			
**60**	**N.D.**	**N.D.**	**40**

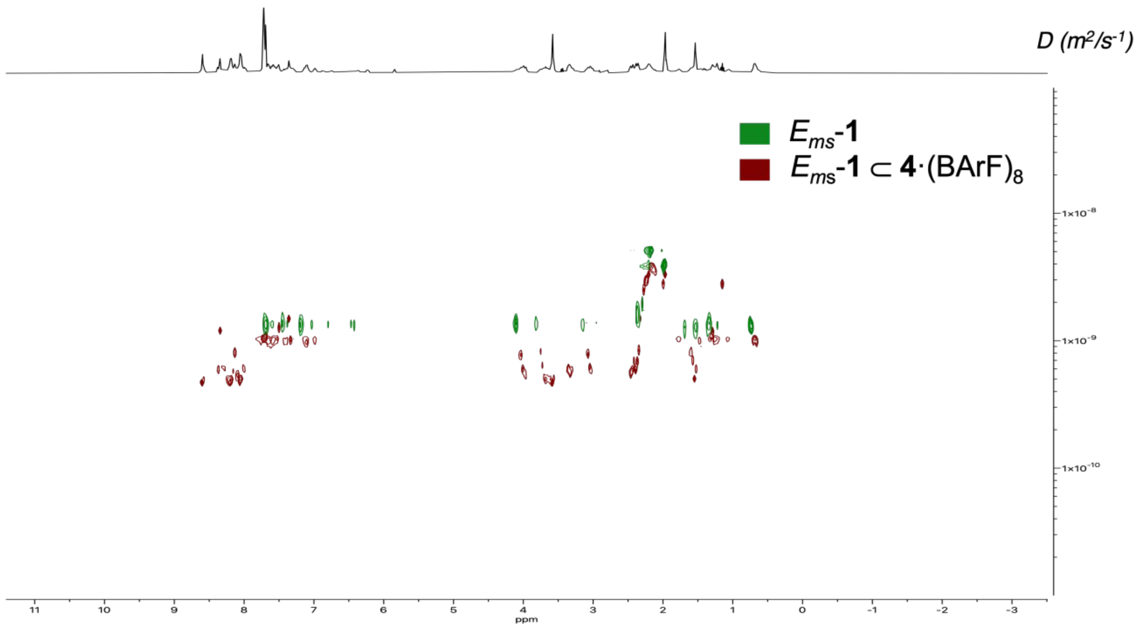

Figure S40. Overlaid ^{1}H DOSY spectra (400 MHz, CD_3CN, 298 K) of the prepared E_{ms}-**1** ⊂ **4**·(BArF)$_8$ host-guest complex (red) and the E_{ms}-**1** (green). The diffusion coefficients for the host and the guest species in CD_3CN were measured to be 3.1×10^{-10} m^2 s^{-1} and 7.9×10^{-10} m^2 s^{-1} respectively. The diffusion coefficient for free E_{ms}-**1** in CD_3CN was measured to be 2.3×10^{-9} m^2 s^{-1}. The differences observed in the diffusion coefficients of the host and the guest for the E_{ms}-**1** ⊂ **4**·(BArF)$_8$ host-guest complex, are indicative of fast exchange binding dynamics on the NMR time scale.

VIII. CD spectroscopy of motor in confined Space

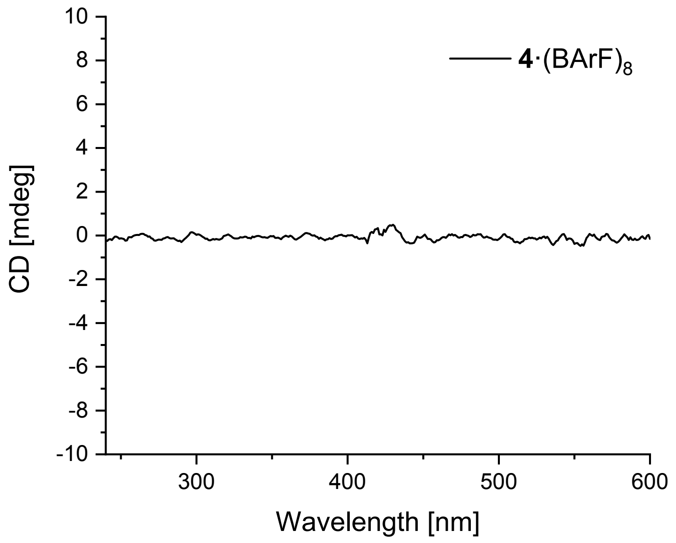

Figure S41. CD spectrum (acetonitrile, ~3x10^{-6} M, 20 °C) of 4·(BArF)$_8$ showing no circular dichroism.

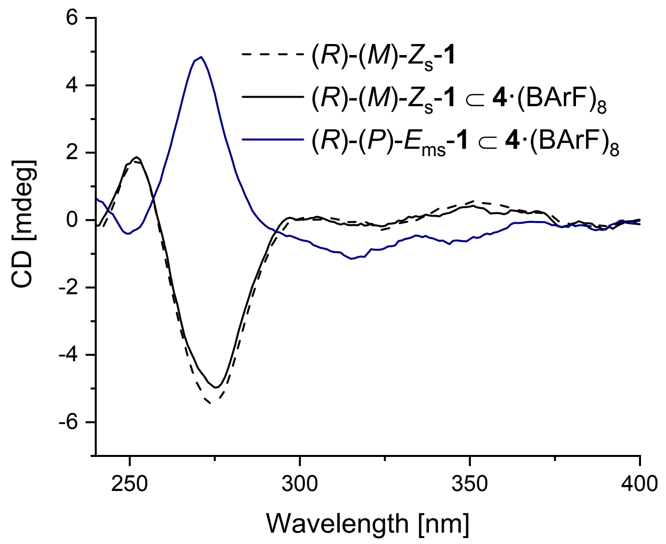

Figure S42. CD spectra (acetonitrile, 1x10^{-6} M **1** and 3x10^{-6} M **4·**(BArF)$_8$, 20 °C) of (*R*)-(*M*)-*Z*$_s$-**1** before irradiation without cage (dashed), at the same concentration in the presence of an excess of **4·**(BArF)$_8$ (black) and encapsulated (*R*)-(*P*)-*E*$_{ms}$-**1** obtained after irradiation with 365 nm UV light in the presence of an excess of cage (blue).

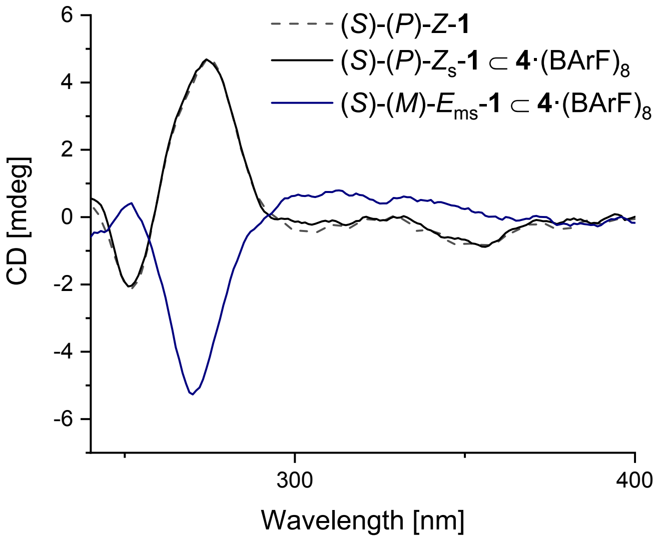

Figure S43. CD spectra (acetonitrile, $1x10^{-6}$ M **1** and $3x10^{-6}$ M **4·**(BArF)$_8$, 20 °C) of (*S*)-(*P*)-*Z*$_s$-**1** before irradiation without cage (dashed), at the same concentration in the presence of an excess of **4·**(BArF)$_8$ (black) and encapsulated (*S*)-(*M*)-*E*$_{ms}$-**1** obtained after irradiation with 365 nm UV light in the presence of an excess of cage (blue)

IX. HPLC chromatograms

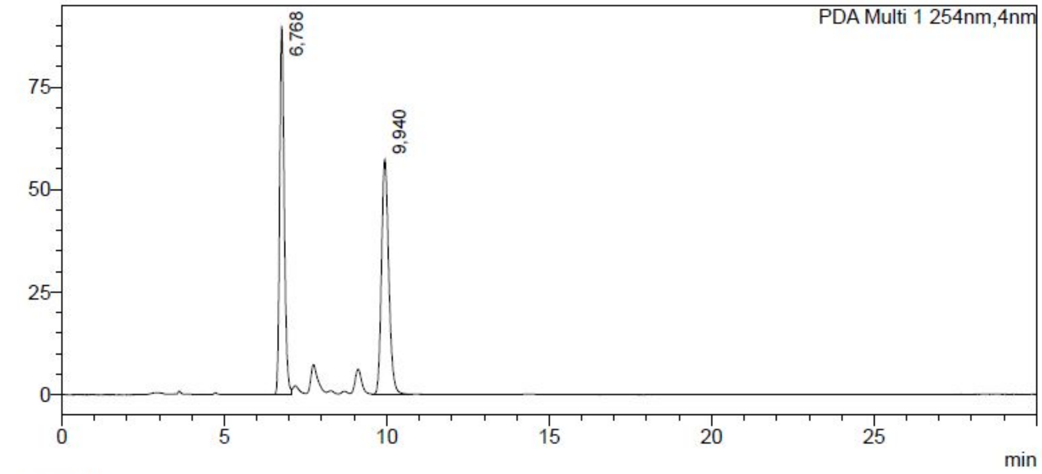

<Peak Table>

PDA Ch1 254nm

Peak#	Ret. Time	Area	Area%
1	6,768	893694	50,165
2	9,940	887802	49,835
Total		1781496	100,000

Figure S44. HPLC Chromatogram of a racemic mixture of (*R*)-(*M*)-Z_s-**1** and (*S*)-(*P*)-Z_s-**1** (Chiralpak IB, *n*-heptane/*i*-PrOH 90:10, 1.0 mL/min, 40 °C).

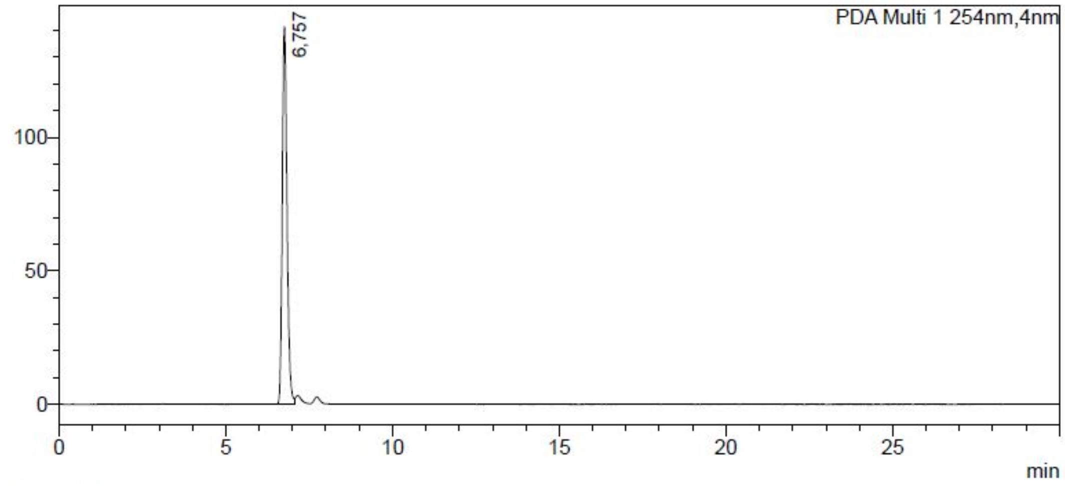

<Peak Table>

PDA Ch1 254nm

Peak#	Ret. Time	Area	Area%
1	6,757	1401691	100,000
Total		1401691	100,000

Figure S45. HPLC Chromatogram of enantiopure (*R*)-(*M*)-Z_s-**1** (Chiralpak IB, *n*-heptane/*i*-PrOH 90:10, 1.0 mL/min, 40 °C) after chromatographic enantiomeric separation.

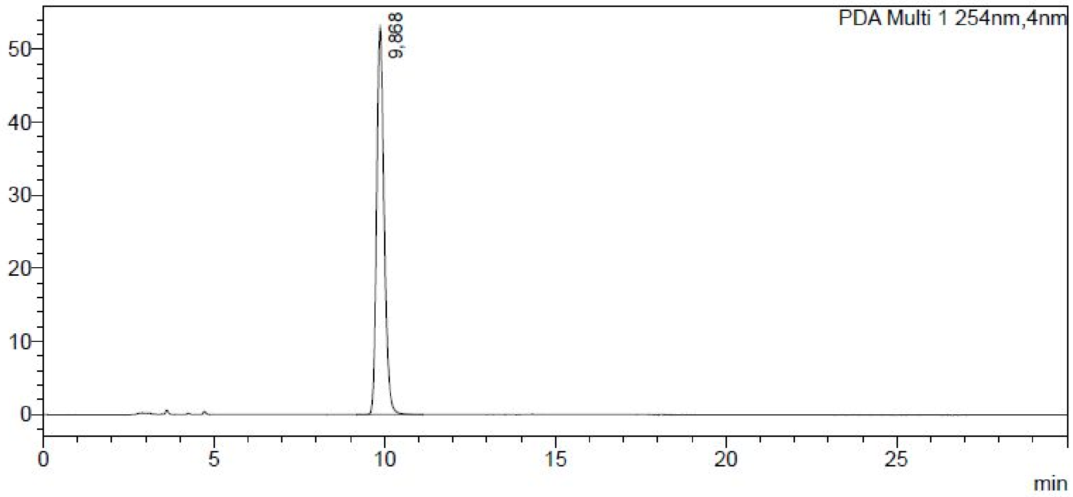

<Peak Table>

PDA Ch1 254nm

Peak#	Ret. Time	Area	Area%
1	9,868	814002	100,000
Total		814002	100,000

Figure S46. HPLC Chromatogram of enantiopure (*S*)-(*P*)-Z_s-**1** (Chiralpak IB, *n*-heptane/*i*-PrOH 90:10, 1.0 mL/min, 40 °C) after chromatographic enantiomeric separation.

X. Computational assignment of stereochemistry

Series of low-lying conformers of (R)- and (S)- **1** were generated using CREST[15–17] (Conformer–Rotamer Ensemble Sampling Tool) at the GFN2-xTB[18] level of theory. The geometry of the most stable conformer of each enantiomer was then optimized with the composite method r^{2}SCAN-3c in the Orca 5.0.4 package,[19] using the conductor-like polarizable continuum CPCM(Acetonitrile) solvent model.[20] Calculations at the time-dependent DFT level of theory (TD-DFT) were then performed, using the previously optimized geometries, the same solvent model and the ωB97X-D3[21,22]/def2-TZVPP[23] without using the Tamm-Dancoff Approximation (no TDA).

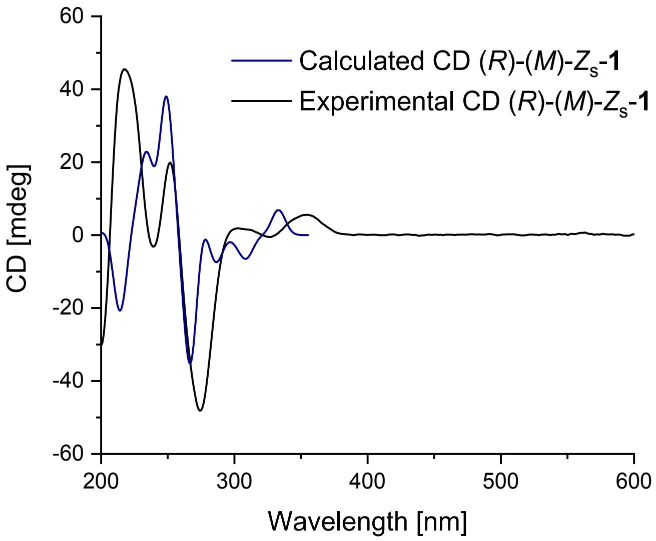

Figure S47. Calculated CD spectrum [ωB97X-D3/Def2-TZVP/CPCM(acetonitrile) // r^{2}SCAN-3c/CPCM(acetonitrile)] of the lowest energy conformer of (*R*)-(*M*)-Z_s-**1** in blue and experimental CD spectrum (black). The intensity of the theoretical spectrum is arbitrary and the calculated wavelength was shifted of +20 nm to allow for a better comparison of the sign inversion induced by excitonic coupling.

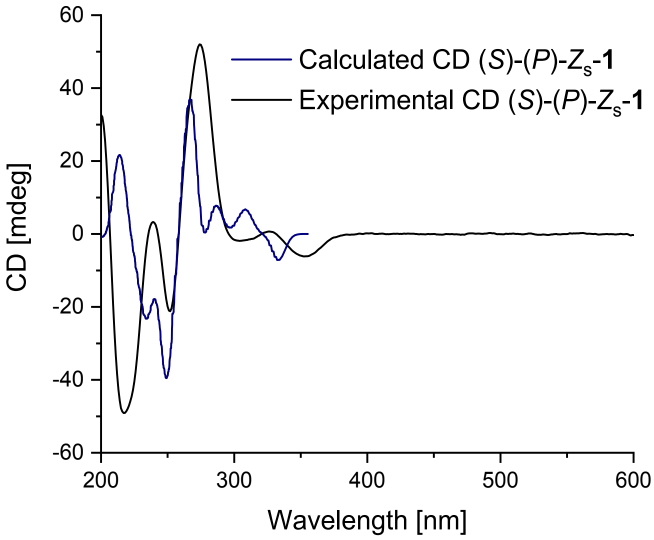

Figure S48. Calculated CD spectrum [ωB97X-D3/Def2-TZVP/CPCM(acetonitrile) // r^{2}SCAN-3c/CPCM(acetonitrile)] of the lowest energy conformer of (*S*)-(*P*)-*Z*s-**1** in blue and experimental CD spectrum (black). The intensity of the theoretical spectrum is arbitrary and the calculated wavelength was shifted of +20 nm to allow for a better comparison of the sign inversion induced by excitonic coupling.

Optimized geometries

Optimized geometry of (*R*)-(*M*)-Z_S-**1**

Final Gibbs free energy: -2296.85770114 Eh

C	-1.18780786012761	-0.04372427882481	-3.96429591484435	C	-2.36113738562294	-1.58617995726558	-0.16695013108827
C	-0.87544660468154	-0.95356078204539	-2.77048198329352	C	-2.15852548980023	-2.89407384599995	-0.62772012142336
H	-1.69531658943217	-1.67381303776854	-2.67691788721445	C	-3.15130567541156	-3.86069327009459	-0.52021173618727
C	0.39248907082485	-1.78555573341142	-2.98966122534793	C	-4.37090818497083	-3.54351304150801	0.07457718731757
S	1.97194225683292	-0.83302251333026	-2.80080910239543	C	-4.57076247556061	-2.27570353183468	0.60749835855940
C	1.45914176698770	0.72953401176076	-2.14236484880300	H	-0.36636915783359	0.64612512773193	-4.17989690603969
C	0.20510683237564	0.95753892545262	-1.58905274003752	H	-2.08693704356727	0.54849041164209	-3.76536589502283
C	-0.14485889092424	2.29629737588086	-1.21595601189891	H	-1.36615724365925	-0.65383357761422	-4.85576098544931
C	0.85614685895586	3.31784895049518	-1.23688032352823	H	0.40797347914446	-2.19432727214872	-4.00588165829162
C	0.52811483804049	4.62749690770361	-0.81263850929146	H	0.44599629653308	-2.61324522283303	-2.27791322104309
C	-0.74903650165736	4.93889966264844	-0.40939106575422	H	1.30676497930435	5.38664835261813	-0.82325083741838
C	-1.75189724270618	3.94755851643570	-0.43962431475081	H	-0.99224649863340	5.94722489195513	-0.08663247246924
C	-1.45842273961769	2.66204037006082	-0.83232445692898	H	-2.76906692462236	4.20168200071357	-0.15325998727922
C	2.15563039805814	3.00217925542872	-1.70621382673614	H	-2.24426387009599	1.91437351975229	-0.85631838838350
C	2.43981753974911	1.75223141767012	-2.18374551869458	H	2.91826549980702	3.77697280878946	-1.71361016579304
C	-0.73586107664601	-0.17929701686116	-1.47585357376860	H	3.42446235228409	1.52951225169654	-2.58739558183893
C	-1.35433423611081	-0.50717408717540	-0.31275975837419	H	-2.76194946086385	1.71214144672360	3.52311784578708
C	-1.09754107503780	0.23080127630750	0.94452190358423	H	-0.44169109307964	2.29125034856250	4.16963755026224
C	-2.16631916951151	0.64301283507151	1.74637846595284	H	2.58058983615188	1.57809065363789	1.05178680290678
C	-1.92941992567488	1.38540097611194	2.90609503344260	H	3.60760959390791	2.00296637872890	2.43441891876773
C	-0.63393080611662	1.70934244822829	3.27317621339275	H	2.39436998452413	-0.77828653213103	2.10379789297709
C	0.44679375898339	1.27055309067618	2.49483653022383	H	3.65133752713865	-0.29938596108533	3.23677182962076
O	1.68499521188259	1.63917072488511	2.93332900796954	H	5.21124441074135	0.18724461620697	1.36778941875712
C	2.81720205775415	1.32570729796641	2.09582052878428	H	3.96585821470791	-0.19554533523399	0.18344609645014
C	3.26381572822718	-0.12423189532763	2.22517297079606	H	4.98502631472155	-2.25658773305203	2.21083392259451
C	4.33835479138701	-0.45390574892601	1.18645759747666	H	5.74019558404456	-1.99484849071419	0.64373909581894
C	4.79094327212491	-1.91822254823954	1.18584438916322	H	4.27333459960268	-3.86978180030748	0.41871932785013
C	3.80845277719637	-2.87722943097424	0.49113915781683	H	3.59383183629731	-2.53127529545728	-0.52436474694565
C	2.48895797363474	-3.07640125060976	1.18256258832376	H	1.74780506728457	-3.60629886122378	2.84302322885770
O	2.63432836466225	-3.48925455128957	2.45953641988672	H	1.01906224621937	0.16042717938249	0.72403780766879
O	1.39581245404718	-2.92054301957303	0.66865725036855	H	-1.20165801010932	-3.16018576216247	-1.06323617002901
C	0.21039203478019	0.51651178553396	1.34885372964529	H	-2.96878142727962	-4.86323824876315	-0.89562988512415
S	-3.83879094054289	0.28098176557603	1.25519562757867	H	-5.15367040298182	-4.29174032909725	0.15859803682274
C	-3.56615058984010	-1.31240865890952	0.50643464782874	H	-5.49725121619988	-2.03693896024223	1.12260456823559

Optimized geometry of (*S*)-(*P*)-Z_S-**1**

Final Gibbs free energy: -2296.85767281 Eh

C	1.18655769014083	0.07617795690117	3.96537882818923	C	2.36733469660474	1.57215798747159	0.15063506546833
C	0.87869572387541	0.97436002991295	2.76161833871890	C	2.17185206309321	2.88599370965972	0.59741670938284
H	1.70177349943168	1.68998627227509	2.66075350173160	C	3.16949871977131	3.84624447200036	0.47877576111856
C	-0.38575521310804	1.81423554330028	2.97061947250845	C	4.38692932470272	3.51639856017261	-0.11347942369737
S	-1.96936860243051	0.86649477864539	2.79118581548186	C	4.57992016475191	2.24184605785820	-0.63264918435943
C	-1.46251438019560	-0.70579739199581	2.15145867633534	H	2.08260445164384	-0.52272764936628	3.77273827545990
C	-0.20940146748468	-0.94486110807660	1.60091832966586	H	0.36166544286754	-0.60708547362554	4.18855746163954
C	0.13561178236476	-2.28906528167253	1.24316386156554	H	1.36816567203719	0.69506041660562	4.85009889776841
C	-0.86896436679287	-3.30675955443549	1.27627349677521	H	-0.43500692183780	2.63384710061195	2.24934919486135
C	-0.54573348363359	-4.62238118106955	0.86713203950297	H	-0.40029002163355	2.23461701176879	3.98210311944848
C	0.73006606974352	-4.94287631225824	0.46677855864337	H	-1.32705186183476	-5.37860484075758	0.88682660655719
C	1.73637060937494	-3.95476473952512	0.48497036846971	H	0.96957236900308	-5.95570015764062	0.15556182603203
C	1.44768097089639	-2.66384629492820	0.86312235604467	H	2.75246146432739	-4.21569618754768	0.20093636333365
C	-2.16722603663010	-2.98088555536964	1.74200808319097	H	2.23622076649815	-1.91883275497032	0.87834812159931
C	-2.44684625121442	-1.72437644874440	2.20479845035927	H	-2.93265771149379	-3.75276223662933	1.75859012264421
C	0.73590688193375	0.18694716530838	1.47526087514936	H	-3.43072460712812	-1.49333499277011	2.60562574690012
C	1.35508454265098	0.49984001180746	0.30848164776506	H	2.74958860280235	-1.76783965671522	-3.50369445171979
C	1.09386119307429	-0.25036754157711	-0.94066552345547	H	0.42603515930011	-2.34105659029034	-4.14364802969109
C	2.16016816944119	-0.67676084239574	-1.73835301744825	H	-3.62250976757631	-2.00868259647528	-2.41485205902581
C	1.91897864971616	-1.43013777941584	-2.88999258734588	H	-2.59539928284018	-1.57436927293608	-1.03532904764782
C	0.62170018163273	-1.75087802608858	-3.25336710852762	H	-3.64205275626637	0.28495265855595	-3.24761962714403
C	-0.45647053520889	-1.29805058174939	-2.47953322180455	H	-2.39117287527232	0.76785484617472	-2.10957925845157
O	-1.69650811458988	-1.66481779062932	-2.91426158198969	H	-3.98302798798550	0.21434760467934	-0.19610363308259
C	-2.82789257991879	-1.33304244350431	-2.08290688516890	H	-5.22142530151182	-0.16813651351399	-1.38793762202897
C	-3.26386480282752	0.11823862771710	-2.23106299637837	H	-5.73463588263203	2.02568185429008	-0.68526067583521
C	-4.34427976814765	0.46661878555065	-1.20475361371062	H	-4.96885076785760	2.26434157085214	-2.25097387537238
C	-4.78332300864973	1.93491512015645	-1.22149741423456	H	-4.25279262280054	3.88891915574959	-0.46982861353837
C	-3.79564309419312	2.89203047763539	-0.53153099423028	H	-3.58796425025167	2.55391108728581	0.48806049186448
C	-2.47174276250667	3.07539511448784	-1.21904342220203	H	-1.71991371318432	3.58250325072781	-2.88199197715724
O	-2.60874931671268	3.47401263703437	-2.50137182560350	H	-1.02229430475898	-0.16660877784718	-0.71999613976265
O	-1.38201804572907	2.91985474897424	-0.69785531376482	H	1.21682301155381	3.16157387623487	1.03096012935384
C	-0.21579205528450	-0.53347180016984	-1.34143509403419	H	2.99242561095874	4.85370689061930	0.84347474426912
S	3.83464697853495	-0.31813627801882	-1.25155849771305	H	5.17343771642812	4.25963821930079	-0.20629692790781
C	3.57050461828088	1.28486376931123	-0.52040557525713	H	5.50485172468732	1.99273728307282	-1.14566611850769

XI. NMR spectra of new compounds

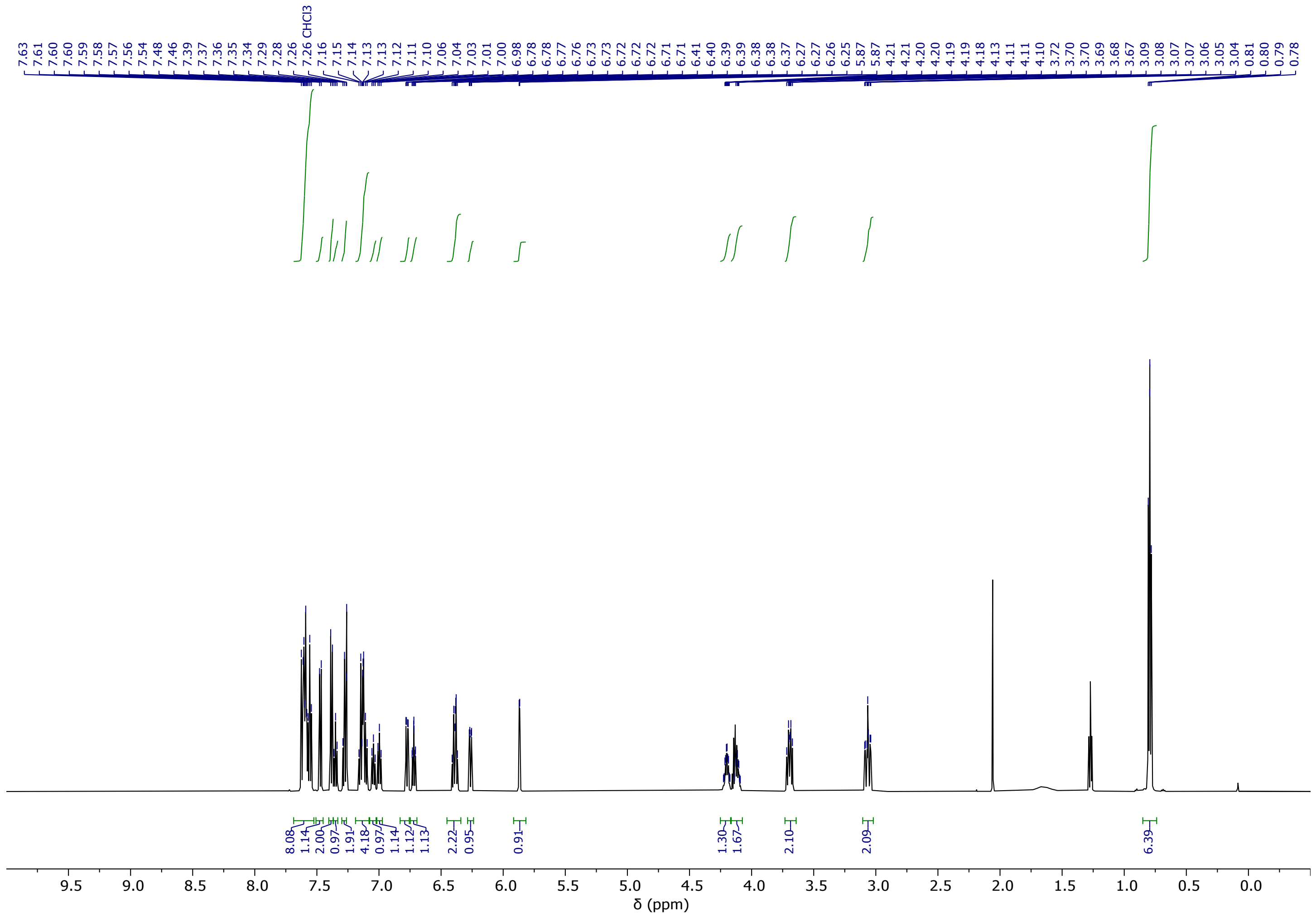

Figure S49. ^{1}H-NMR of **3** (600 MHz, $CDCl_3$, 25 °C).

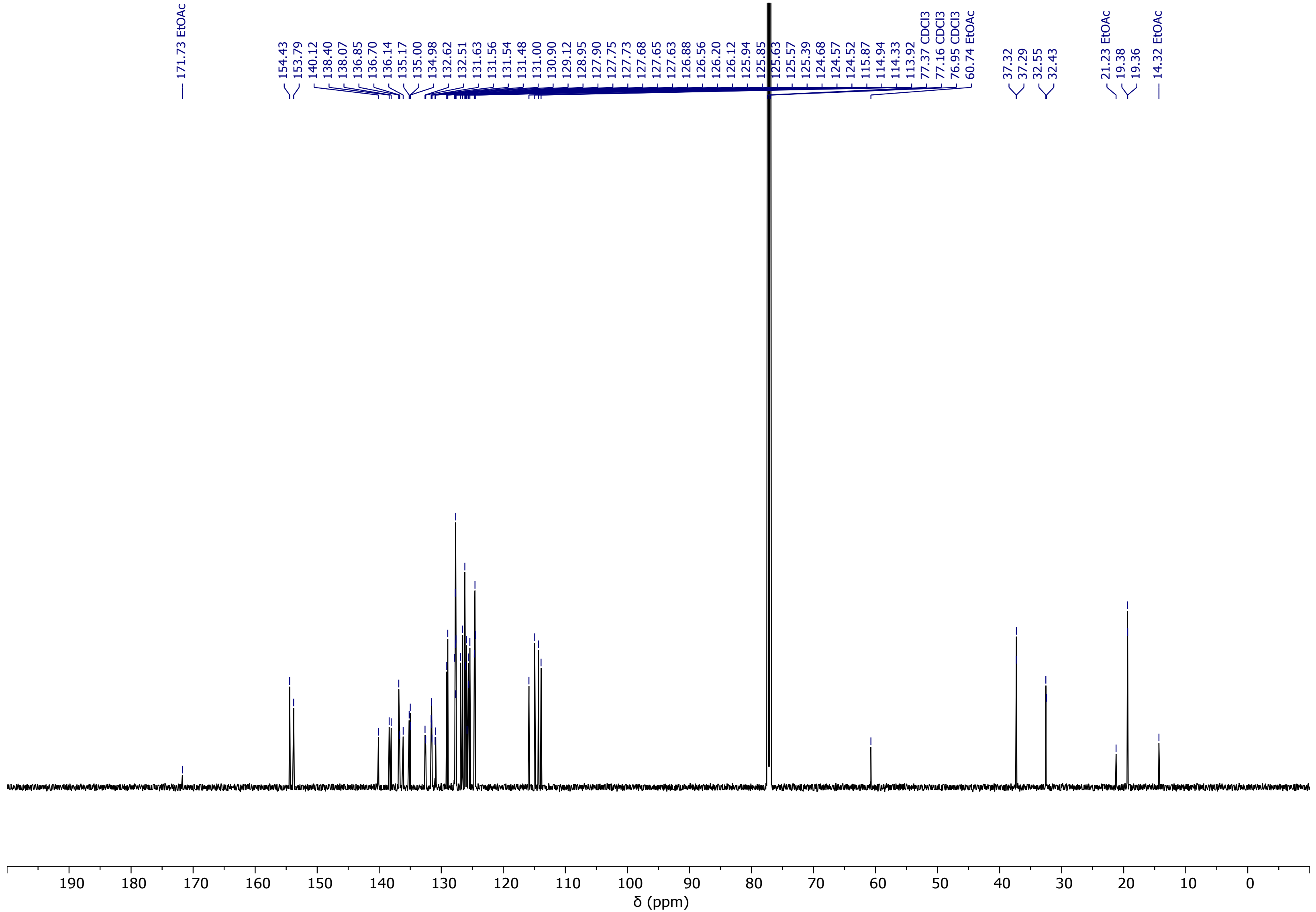

Figure S50. $^{13}C\{^{1}H\}$-NMR of **3** (151 MHz, $CDCl_3$, 25 °C).

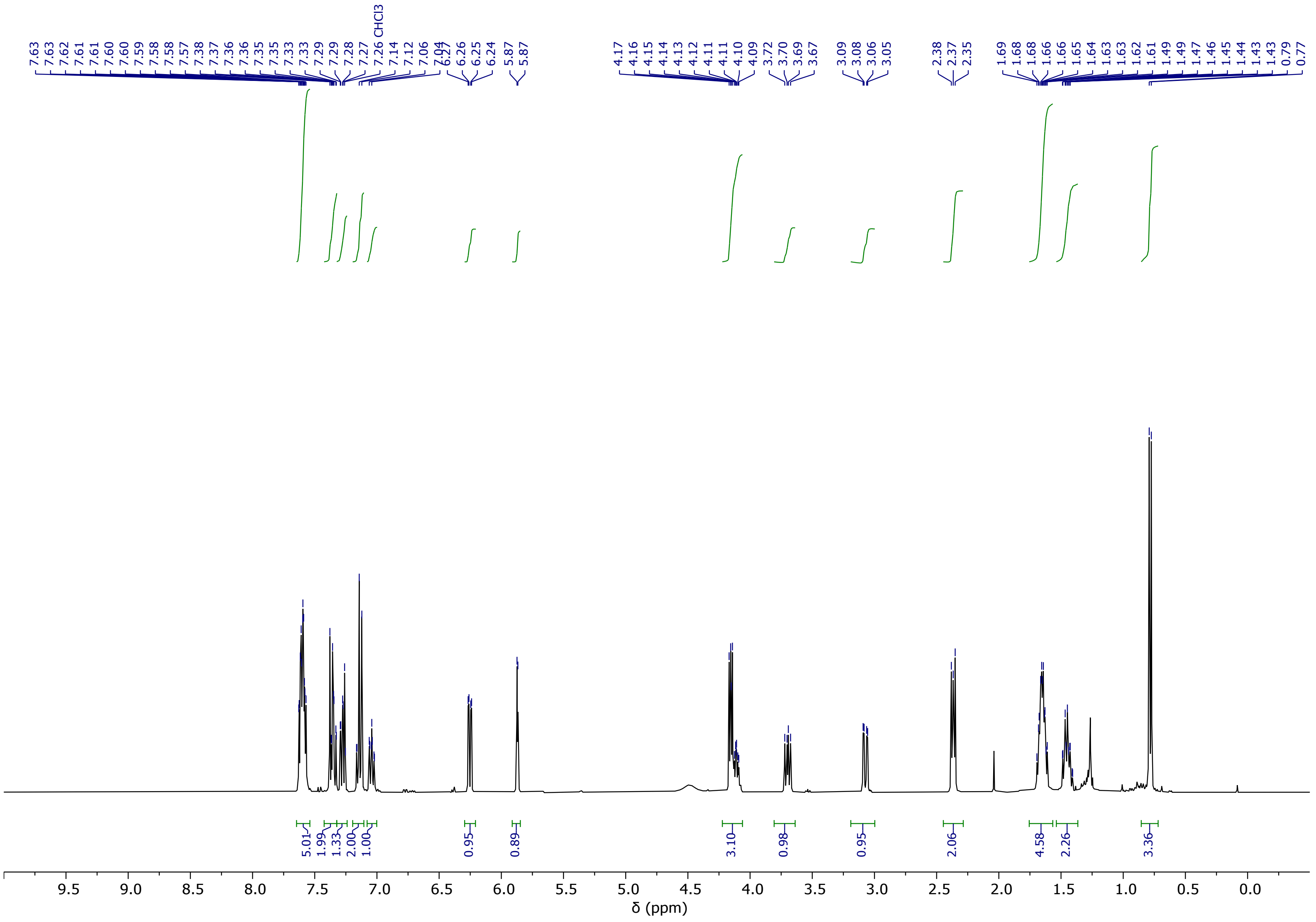

Figure S51. ^{1}H-NMR of Z_s-**1** (400 MHz, $CDCl_3$, 25 °C).

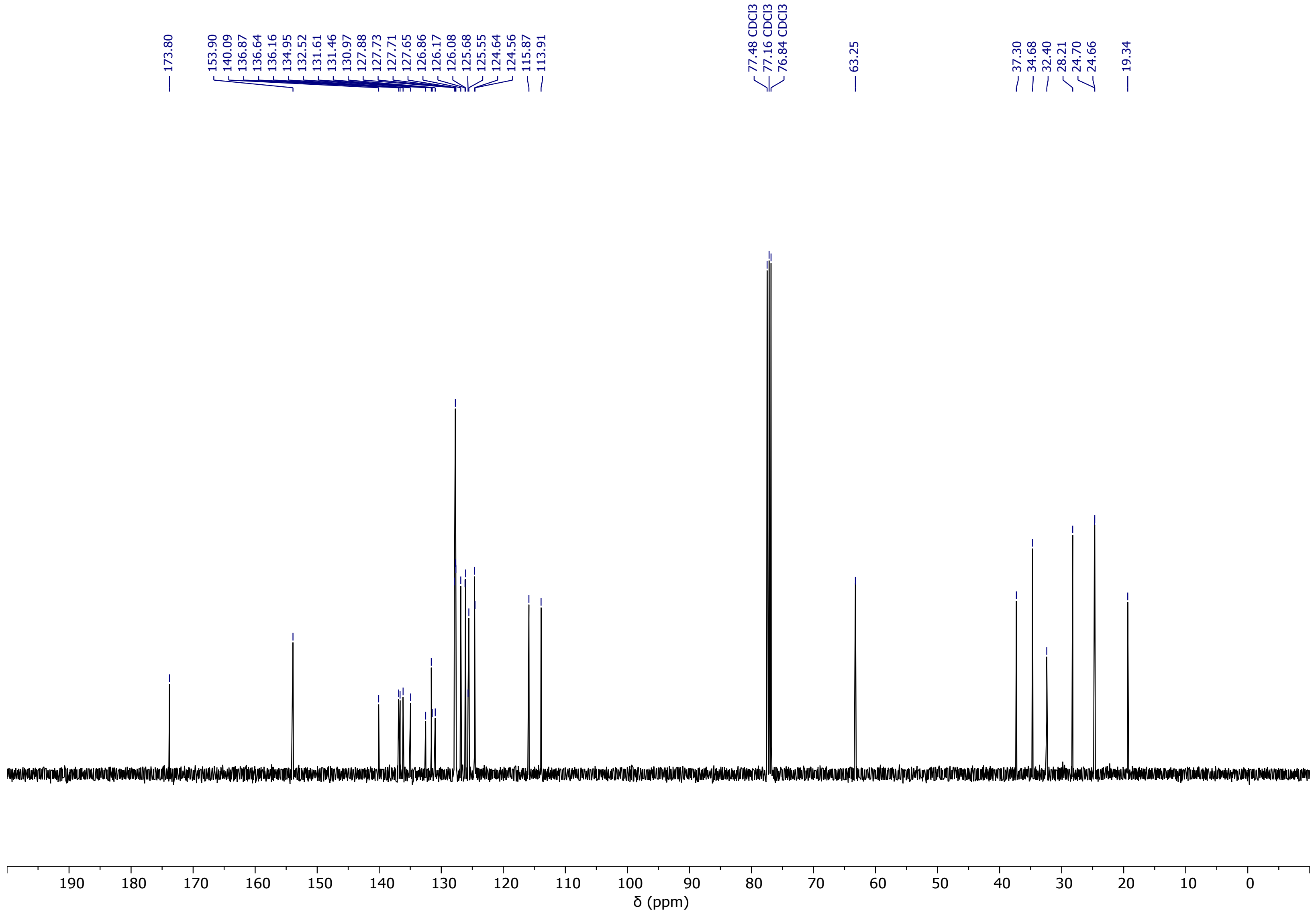

Figure S52. $^{13}C\{^{1}H\}$-NMR of *Z*$_{s}$-**1** (101 MHz, $CDCl_3$, 25 °C).

XII. References

(1) Koumura, N.; Geertsema, E. M.; Meetsma, A.; Feringa, B. L. Light-Driven Molecular Rotor: Unidirectional Rotation Controlled by a Single Stereogenic Center. *J. Am. Chem. Soc.* **2000**, *122* (48), 12005–12006.

(2) Koumura, N.; Geertsema, E. M.; van Gelder, M. B.; Meetsma, A.; Feringa, B. L. Second Generation Light-Driven Molecular Motors. Unidirectional Rotation Controlled by a Single Stereogenic Center with Near-Perfect Photoequilibria and Acceleration of the Speed of Rotation by Structural Modification. *J. Am. Chem. Soc.* **2002**, *124* (18), 5037–5051.

(3) García-Simón, C.; Garcia-Borràs, M.; Gómez, L.; Parella, T.; Osuna, S.; Juanhuix, J.; Imaz, I.; Maspoch, D.; Costas, M.; Ribas, X. Sponge-like Molecular Cage for Purification of Fullerenes. *Nat. Commun.* **2014**, *5* (1), 5557.

(4) Crowley, J. D.; Goshe, A. J.; Bosnich, B. Molecular Recognition. Self-Assembly of Molecular Trigonal Prisms and Their Host–Guest Adducts. *Chem. Commun.* **2003**, No. 22, 2824–2825.

(5) García-Simón, C.; Colomban, C.; Çetin, Y. A.; Gimeno, A.; Pujals, M.; Ubasart, E.; Fuertes-Espinosa, C.; Asad, K.; Chronakis, N.; Costas, M.; Jiménez-Barbero, J.; Feixas, F.; Ribas, X. Complete Dynamic Reconstruction of C60, C70, and (C59N)2 Encapsulation into an Adaptable Supramolecular Nanocapsule. *J. Am. Chem. Soc.* **2020**, *142* (37), 16051–16063.

(6) Wang, J.; Wolf, R. M.; Caldwell, J. W.; Kollman, P. A.; Case, D. A. Development and Testing of a General Amber Force Field. *J. Comput. Chem.* **2004**, *25* (9), 1157–1174.

(7) Li, P.; Merz, K. M. Jr. MCPB.Py: A Python Based Metal Center Parameter Builder. *J. Chem. Inf. Model.* **2016**, *56* (4), 599–604.

(8) Bayly, C. I.; Cieplak, P.; Cornell, W.; Kollman, P. A. A Well-Behaved Electrostatic Potential Based Method Using Charge Restraints for Deriving Atomic Charges: The RESP Model. *J. Phys. Chem.* **1993**, *97* (40), 10269–10280.

(9) Frisch, M. J.; Trucks, G. W.; Schlegel, H. B.; Scuseria, G. E.; Robb, M. A.; Cheeseman, J. R.; Scalmani, G.; Barone, V.; Petersson, G. A.; Nakatsuji, H.; Li, X.; Caricato, M.; Marenich, A. V.; Bloino, J.; Janesko, B. G.; Gomperts, R.; Mennucci, B.; Hratchian, H. P.; Ortiz, J. V.; Izmaylov, A. F.; Sonnenberg, J. L.; Williams; Ding, F.; Lipparini, F.; Egidi, F.; Goings, J.; Peng, B.; Petrone, A.; Henderson, T.; Ranasinghe, D.; Zakrzewski, V. G.; Gao, J.; Rega, N.; Zheng, G.; Liang, W.; Hada, M.; Ehara, M.; Toyota, K.; Fukuda, R.; Hasegawa, J.; Ishida, M.; Nakajima, T.; Honda, Y.; Kitao, O.; Nakai, H.; Vreven, T.; Throssell, K.; Montgomery Jr., J. A.; Peralta, J. E.; Ogliaro, F.; Bearpark, M. J.; Heyd, J. J.; Brothers, E. N.; Kudin, K. N.; Staroverov, V. N.; Keith, T. A.; Kobayashi, R.; Normand, J.; Raghavachari, K.; Rendell, A. P.; Burant, J. C.; Iyengar, S. S.; Tomasi, J.; Cossi, M.; Millam, J. M.; Klene, M.; Adamo, C.; Cammi, R.; Ochterski, J. W.; Martin, R. L.; Morokuma, K.; Farkas, O.; Foresman, J. B.; Fox, D. J.; Gaussian Inc.; Wallingford CT. Gaussian 16 Rev. C.01, 2016.

(10) Case, D. A.; Aktulga, H. M.; Belfon, K.; Ben-Shalom, I. Y.; Berryman, J. T.; Brozell, S. R.; Cerutti, D. S.; Cheatham, T. E.; Cisneros, G. A.; Cruzeiro, V. W. D.; Darden, T. A.; Duke, R. E.; Giambasu, G.; Gilson, M. K.; Gohlke, H.; Goetz, A. W.; Harris, R.; Izadi, S.; Izmailov, S. A.; Kasavajhala, K.; Kaymak, M. C.; King, E.; Kovalenko, A.; Kurtzman, T.; Lee, T. S.; LeGrand, S.; Li, P.; Lin, C.; Liu, J.; Luchko, T.; Luo, R.; Machado, M.; Man, V.; Manathunga, M.; Merz, K. M.; Miao, Y.; Mikhailovskii, O.; Monard, G.; Nguyen, H.; O'Hearn, K. A.; Onufriev, A.; Pan, F.; Pantano, S.; Qi, R.; Rahnamoun, A.; Roe, D. R.; Roitberg, A.; Sagui, C.; Schott-Verdugo, S.; Shajan, A.; Shen, J.; Simmerling, C. L.; Skrynnikov, N. R.; Smith, J.; Swails, J.; Walker, R. C.; Wang, J.; Wang, J.; Wei, H.; Wolf, R. M.; Wu, X.; Xiong, Y.; Xue, Y.; York, D. M.; Zhao, S.; Kollman, P. A.; Amber 2022; University of California; San Francisco. Amber 2022, University of California, San Francisco, 2022.

(11) Salomon-Ferrer, R.; Götz, A. W.; Poole, D.; Le Grand, S.; Walker, R. C. Routine Microsecond Molecular Dynamics Simulations with AMBER on GPUs. 2. Explicit Solvent Particle Mesh Ewald. *J. Chem. Theory Comput.* **2013**, *9* (9), 3878–3888.

(12) Essmann, U.; Perera, L.; Berkowitz, M. L.; Darden, T.; Lee, H.; Pedersen, L. G. A Smooth Particle Mesh Ewald Method. *J. Chem. Phys.* **1995**, *103* (19), 8577–8593.

(13) Maglic, J. B.; Lavendomme, R. MoloVol: An Easy-to-Use Program for Analyzing Cavities, Volumes and Surface Areas of Chemical Structures. *J. Appl. Crystallogr.* **2022**, *55* (4), 1033–1044.

(14) Boto, R. A.; Peccati, F.; Laplaza, R.; Quan, C.; Carbone, A.; Piquemal, J.-P.; Maday, Y.; Contreras-García, J. NCIPLOT4: Fast, Robust, and Quantitative Analysis of Noncovalent Interactions. *J. Chem. Theory Comput.* **2020**, *16* (7), 4150–4158.

(15) Grimme, S. Exploration of Chemical Compound, Conformer, and Reaction Space with Meta-Dynamics Simulations Based on Tight-Binding Quantum Chemical Calculations. *J. Chem. Theory Comput.* **2019**, *15* (5), 2847–2862.

(16) Pracht, P.; Bohle, F.; Grimme, S. Automated Exploration of the Low-Energy Chemical Space with Fast Quantum Chemical Methods. *Phys. Chem. Chem. Phys.* **2020**, *22* (14), 7169–7192.

(17) Pracht, P.; Grimme, S.; Bannwarth, C.; Bohle, F.; Ehlert, S.; Feldmann, G.; Gorges, J.; Müller, M.; Neudecker, T.; Plett, C.; Spicher, S.; Steinbach, P.; Wesołowski, P. A.; Zeller, F. CREST—A Program for the Exploration of Low-Energy Molecular Chemical Space. *J. Chem. Phys.* **2024**, *160* (11), 114110.

(18) Bannwarth, C.; Ehlert, S.; Grimme, S. GFN2-xTB—An Accurate and Broadly Parametrized Self-Consistent Tight-Binding Quantum Chemical Method with Multipole Electrostatics and Density-Dependent Dispersion Contributions. *J. Chem. Theory Comput.* **2019**, *15* (3), 1652–1671.

(19) Neese, F.; Wennmohs, F.; Becker, U.; Riplinger, C. The ORCA Quantum Chemistry Program Package. *J. Chem. Phys.* **2020**, *152* (22), 224108.

(20) Barone, V.; Cossi, M. Quantum Calculation of Molecular Energies and Energy Gradients in Solution by a Conductor Solvent Model. *J. Phys. Chem. A* **1998**, *102* (11), 1995–2001.

(21) Grimme, S.; Antony, J.; Ehrlich, S.; Krieg, H. A Consistent and Accurate Ab Initio Parametrization of Density Functional Dispersion Correction (DFT-D) for the 94 Elements H-Pu. *J. Chem. Phys.* **2010**, *132* (15), 154104.

(22) Lin, Y.-S.; Li, G.-D.; Mao, S.-P.; Chai, J.-D. Long-Range Corrected Hybrid Density Functionals with Improved Dispersion Corrections. *J. Chem. Theory Comput.* **2013**, *9* (1), 263–272.

(23) Weigend, F.; Ahlrichs, R. Balanced Basis Sets of Split Valence, Triple Zeta Valence and Quadruple Zeta Valence Quality for H to Rn: Design and Assessment of Accuracy. *Phys. Chem. Chem. Phys.* **2005**, *7* (18), 3297–3305.
